# Intermittent fasting from dawn to sunset for four consecutive weeks induces anticancer serum proteome response and improves metabolic syndrome

**DOI:** 10.1038/s41598-020-73767-w

**Published:** 2020-10-27

**Authors:** Ayse L. Mindikoglu, Mustafa M. Abdulsada, Antrix Jain, Prasun K. Jalal, Sridevi Devaraj, Zoe R. Wilhelm, Antone R. Opekun, Sung Yun Jung

**Affiliations:** 1 Margaret M. and Albert B. Alkek Department of Medicine, Section of Gastroenterology and Hepatology, Baylor College of Medicine, Houston, TX USA; 2 Michael E. DeBakey Department of Surgery, Division of Abdominal Transplantation, Baylor College of Medicine, Houston, TX USA; 3 Advanced Technology Core, Mass Spectrometry Proteomics Core, Baylor College of Medicine, Houston, TX USA; 4 Clinical Chemistry and Point of Care Technology, Department of Pathology and Immunology, Texas Children’s Hospital and Health Centers, Baylor College of Medicine, Houston, TX USA; 5 Department of Pediatrics, Section of Gastroenterology, Nutrition and Hepatology, Baylor College of Medicine, Houston, TX USA; 6 Department of Molecular and Cellular Biology, Baylor College of Medicine, Houston, TX USA

**Keywords:** Diabetes, Metabolic syndrome, Obesity, Cancer, Proteomic analysis

## Abstract

Metabolic syndrome is characterized by central obesity, insulin resistance, elevated blood pressure, and dyslipidemia. Metabolic syndrome is a significant risk factor for several common cancers (e.g., liver, colorectal, breast, pancreas). Pharmacologic treatments used for the components of the metabolic syndrome appear to be insufficient to control cancer development in subjects with metabolic syndrome. Murine models showed that cancer has the slowest progression when there is no food consumption during the daily activity phase. Intermittent fasting from dawn to sunset is a form of fasting practiced during human activity hours. To test the anticancer effect of intermittent fasting from dawn to sunset in metabolic syndrome, we conducted a pilot study in 14 subjects with metabolic syndrome who fasted (no eating or drinking) from dawn to sunset for more than 14 h daily for four consecutive weeks. We collected serum samples before 4-week intermittent fasting, at the end of 4th week during 4-week intermittent fasting and 1 week after 4-week intermittent fasting. We performed serum proteomic analysis using nano ultra-high performance liquid chromatography-tandem mass spectrometry. We found a significant fold increase in the levels of several tumor suppressor and DNA repair gene protein products (GP)s at the end of 4th week during 4-week intermittent fasting (CALU, INTS6, KIT, CROCC, PIGR), and 1 week after 4-week intermittent fasting (CALU, CALR, IGFBP4, SEMA4B) compared with the levels before 4-week intermittent fasting. We also found a significant reduction in the levels of tumor promoter GPs at the end of 4th week during 4-week intermittent fasting (POLK, CD109, CAMP, NIFK, SRGN), and 1 week after 4-week intermittent fasting (CAMP, PLAC1) compared with the levels before 4-week intermittent fasting. Fasting from dawn to sunset for four weeks also induced an anti-diabetes proteome response by upregulating the key regulatory proteins of insulin signaling at the end of 4th week during 4-week intermittent fasting (VPS8, POLRMT, IGFBP-5) and 1 week after 4-week intermittent fasting (PRKCSH), and an anti-aging proteome response by upregulating H2B histone proteins 1 week after 4-week intermittent fasting. Subjects had a significant reduction in body mass index, waist circumference, and improvement in blood pressure that co-occurred with the anticancer, anti-diabetes, and anti-aging serum proteome response. These findings suggest that intermittent fasting from dawn to sunset actively modulates the respective genes and can be an adjunct treatment in metabolic syndrome. Further studies are needed to test the intermittent fasting from dawn to sunset in the prevention and treatment of metabolic syndrome-induced cancers.

## Introduction

One of the grand challenges of our times is the rising prevalence of metabolic syndrome^1^. Metabolic syndrome is characterized by central obesity, insulin resistance, elevated blood pressure, high triglyceride, and low high-density lipoprotein levels^2^. Metabolic syndrome has adversely impacted many aspects of society^3^. Importantly, metabolic syndrome is a significant risk factor for several common cancers (e.g., liver, colorectal, breast, endometrium, pancreas)^4,5^.

Disrupted circadian clock rhythm has been recognized as one of the causes of metabolic syndrome and metabolic syndrome-induced cancers^6–8^. Pharmacologic treatments used for the components of the metabolic syndrome appear to be insufficient to reduce the risk of developing metabolic syndrome-induced cancers^9^. Moreover, pharmacologic treatments cannot reset the circadian clock rhythm; thus, there is an urgent need for an effective intervention to reset the circadian clock and prevent metabolic syndrome and metabolic syndrome-induced cancers. Animal studies showed that resetting the circadian clock by time-restricted feeding improves metabolic syndrome and inhibits the development of cancer^10,11^. Therefore, resetting the disrupted circadian clock in humans by consecutive daily intermittent fasting could provide a primary strategy to improve metabolic syndrome and reduce the incidence of metabolic syndrome-induced cancer^10–12^. Fasting during activity hours in contrast to the inactivity hours of the day appears to be important for the optimization of anticancer effect and gene expression. Mice with no access to food during the activity phase (dark phase) of a 12-h light/12-h dark cycle had a significantly slower tumor progression and higher survival compared with mice that had no access to food during the inactivity phase (light phase), and mice that had access to food ad libitum^13^. Consistent with these findings, an earlier murine study showed that the uncoupling of the peripheral clocks from the control of the central clock only occurred when mice had no access to food during the active phase^14^. In contrast, a minimal change in the phase of gene expression was observed when mice had no access to food during the inactive phase^14^, corresponding to night time in humans. The findings of murine studies should be interpreted in the context of the fact that humans are diurnal (activity occurs during daytime), and mice are nocturnal (activity occurs during nighttime). The findings of these murine studies are in accord with the findings of our preliminary studies conducted in healthy subjects^15^. Our results showed that 30-day intermittent fasting from dawn to sunset, the human activity phase, was associated with an anticancer serum proteome response and upregulated several key regulatory proteins that play a key role in tumor suppression, DNA repair, insulin signaling, glucose, and lipid metabolism, circadian clock, cytoskeletal remodeling, immune system, and cognitive function^15^. Importantly, the increase in the levels of these critical regulatory proteins occurred in the absence of any significant weight loss and calorie restriction^15^. Several human studies showed beneficial effects of intermittent fasting (e.g., Ramadan fasting^16–18^), and time-restricted eating^19^ in subjects with metabolic syndrome. However, in none of these studies, proteomic profiling was performed to understand the mechanism behind the anticancer effect of intermittent fasting and time-restricted eating in subjects with metabolic syndrome.

To this end, we hypothesized that intermittent fasting from dawn to sunset practiced exclusively during the human activity hours for four weeks would be associated with an anticancer serum proteome response, upregulate anticancer proteins and regulatory proteins of DNA repair and insulin signaling, and downregulate pro-cancer proteins.

## Methods

### Study subjects

This study was approved by the Institutional Review Board of the Baylor College of Medicine Biomedical Research and Assurance Information Network (BRAIN) under protocol number H-31612. All research was conducted in accordance with relevant guidelines and regulations after written informed consent was obtained from all subjects. The study did not qualify for prospective registration as a clinical trial because there was no study directed intervention. The religious fast was habitual personal conduct, not study directed, and as such, the study was observational in study design. Inclusion criteria were as follows: (1) Subjects who are 18 years old or older; (2) Subjects who plan to fast during the religious month of Ramadan^16^; (3) Subjects should meet any three of the following five criteria for metabolic syndrome as described by Grundy et al.^2^ (a) central obesity assessed by waist circumference equal to or greater than 102 cm (40 inches) in men, and equal or greater than 88 cm (35 inches) in women; (b) fasting serum triglyceride level equal to or greater than 150 mg/dL or on drug therapy for hypertriglyceridemia (e.g., fibrates, nicotinic acid); (c) low high-density lipoprotein level less than 40 mg/dL in men and less than 50 mg/dL in women or on drug therapy for low high-density lipoprotein level (fibrates, nicotinic acid); (d) elevated systolic blood pressure equal to or greater than 130 or elevated diastolic blood pressure equal to or greater than 85 or on drug therapy for hypertension, (e) elevated fasting glucose level equal to or greater than 100 mg/dL or on drug therapy for hyperglycemia/diabetes); (4) Subjects who agreed to undergo FibroScan^20^ testing for evaluation of hepatic steatosis and fibrosis^20^. Subjects were excluded if they had any of the following: (1) inability to provide informed consent; (2) women who are pregnant or breastfeeding; (3) active cancer; (4) active infection requiring antibiotic use; (5) seizure disorder; (6) cardiovascular event during the last 6 months; (7) use of alcohol or recreational substances.

The primary outcome of this pilot study was the induction of an anticancer proteome response at the end of 4th week during 4-week intermittent fasting and 1 week after 4-week intermittent fasting. The secondary outcomes were improvement in the components of metabolic syndrome, lipid panel (total cholesterol, triglyceride, high-density lipoprotein, and low-density lipoprotein), hepatic panel (albumin, total protein, alanine aminotransferase, aspartate aminotransferase, alkaline phosphatase), and adiposity, oxidative stress, and inflammation biomarkers.

### Study procedures

Subjects were scheduled for a screening visit within three weeks of initiation of 4-week intermittent fasting at Baylor College of Medicine in the Texas Medical Center Digestive Diseases Center Clinical Research Core E Laboratory. During this visit, their eligibility was assessed based on inclusion and exclusion criteria, and written informed consent was taken. Medical history and physical examination were performed. Blood pressure measurements were performed at rest and sitting position. A urine pregnancy test for the female subjects at childbearing age was performed.

Hepatic steatosis and fibrosis were assessed using FibroScan with a controlled attenuation parameter (CAP)^20^.

Subjects started fasting at dawn after a pre-dawn breakfast and ended fasting at sunset (dusk) with a dinner for 29 consecutive days. Strict fasting occurred without eating or drinking between dawn and sunset (dusk), which are symmetrical transition time zones of the day. There was no interventional calorie or energy restriction otherwise. Subjects had their main meals at the transition time zones of the day, including pre-dawn breakfast (at the first transition time of the day) and dinner at sunset (at the second transition time of the day) and were allowed to eat (e.g., snacks) or drink if they needed between sunset and dawn in addition to the pre-dawn breakfast and dinner at sunset.

Data on weight, waist circumference and blood pressure and blood specimens were collected within three weeks before the initiation of 4-week intermittent fasting to assess the effect of ad libitum eating, at the end of 4th week during 4-week intermittent fasting to assess the effect of intermittent fasting and 1 week after 4-week intermittent fasting to assess the carryover effect of intermittent fasting on serum proteome, components of metabolic syndrome, lipid and hepatic panels, and adiposity, oxidative stress, and inflammation biomarkers. At each time point, data on weight, waist circumference, and blood pressure and blood specimens were collected after at least 8 h of fasting.

The compliance with fasting was monitored by a 13C-isotopic breath enrichment test, as previously described^15,21^.

### Serum proteomics

We previously described a robust, streamlined proteomic approach to perform quantitative analysis of human serum samples using nano ultra-highperformance liquid chromatography-tandem mass spectrometry (nano UHPLC-MS/MS)^15^. Briefly, 10 μl of serum was incubated with the top 12 abundant serum protein depletion kit (Thermo Scientific Pierce, Cat# 85164) and digested with trypsin on S-Trap column (ProtiFi, NY). The digested peptide was eluted, vacuum dried, and fractionated using high pH STAGE (Stop-and-go extraction) method into two pools, then subjected to nano-HPLC–MS/MS analysis. The parameters for mass spectrometry analysis and the process for mass analysis is maintained the same as previous publication^15^. We also explained the details of the gene protein product (GP)s quantification in our previous publication^15^. Briefly, we quantified GPs using the label-free, intensity-based absolute quantification (iBAQ) method and then normalized to final quantificational value (FOT) defined as the iBAQ value of an individual protein divided by the total iBAQ values of all identified proteins within one experiment. The FOT represents the relative abundance of each GP. The FOT values of a particular GP in different conditions (e.g., FOT value of a GP before 4-week intermittent fasting and at the end of 4th week during 4-week intermittent fasting) can be divided to get a fold change. FOT value also provides the relative quantification of different GPs in the same condition (e.g., FOT value of two different GPs at the end of 4th week during 4-week intermittent fasting).

### Statistical analysis

For statistical analysis of serum proteomics, we used Excel application (Microsoft, Redmond, WA, USA). To determine statistically significantly regulated protein levels at the end of 4th week during 4-week intermittent fasting and 1 week after 4-week intermittent fasting, we performed paired two-tailed student’s t-test using log converted iFOT values^15^. We considered protein levels that showed an equal to or greater than fourfold average paired change and a P value of < 0.05 as significant^15^. We performed a volcano plot analysis to display the GPs that had an equal to or greater than fourfold significant change at the end of 4-week intermittent fasting during 4-week intermittent fasting and 1 week after 4-week intermittent fasting compared with the levels before 4-week intermittent fasting^15^.

### Components of metabolic syndrome, lipid and hepatic panels, adiposity, oxidative stress and inflammation biomarkers

We measured the components of metabolic syndrome, lipid panel, hepatic panel and adiposity, oxidative stress, and inflammation biomarkers within three weeks before 4-week intermittent fasting, at the end of 4-week intermittent fasting during 4-week intermittent fasting, and 1 week after 4-week intermittent fasting. We estimated the insulin resistance by using Homeostatic Model Assessment for Insulin Resistance (HOMA-IR) equation as described by Matthews et al.^22^ We calculated the mean arterial blood pressure using the following formula: (diastolic blood pressure) + [(systolic blood pressure-diastolic blood pressure)/3]^23^.

### Statistical analysis

We used SAS Version 9.4 TS Level 1M5 X64_10PRO platform (SAS, Cary, NC, USA)^24^ to perform statistical analysis of the components of metabolic syndrome, lipid panel, hepatic panel, and adiposity, oxidative stress, and inflammation biomarkers. We performed a student’s paired t-test to determine statistically significant changes in the levels of the components of metabolic syndrome, lipid panel, hepatic panel, and adiposity, oxidative stress, and inflammation biomarkers measured at the end of 4th week during 4-week intermittent fasting, and 1 week after 4-week intermittent fasting. We calculated Pearson’s correlation coefficient to assess correlations between significant GPs (i.e., GPs that showed significant fold changes at the end of 4th week during 4-week intermittent fasting and 1 week after 4-week intermittent fasting) and the components of metabolic syndrome, lipid panel, hepatic panel, and adiposity, oxidative stress, and inflammation biomarkers. In these analyses, we considered a two-tailed *P* value of ˂ 0.05 statistically significant.

## Results

### Subjects

**Figure 1:**
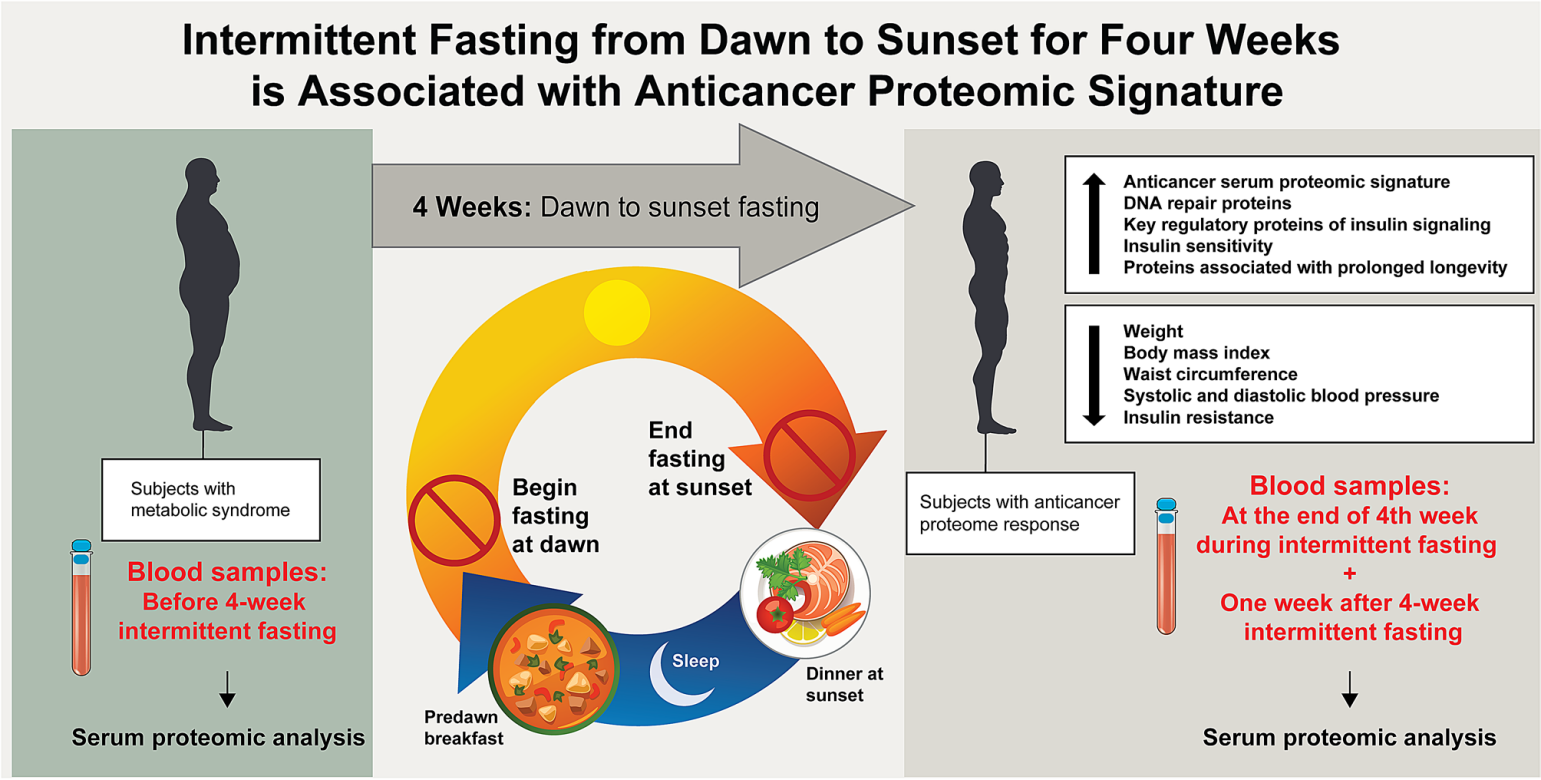
Intermittent fasting from dawn to sunset (dusk) for four weeks. Subjects fasted (no eating or drinking) for more than 14 h. daily for 29 days, from May 06, 2019, until June 03, 2019. The minimum required duration of daily fasting from dawn to sunset was 14 h., 8 min for the shortest day (May 6, 2019), and 14 h., 42 min for the longest day (June 03, 2019). Ramadan fasting is a unique form of intermittent fasting from dawn to sunset (dusk) without eating or drinking during the month of Ramadan based on the lunar calendar^16^ and has several major unique features: (1) Fasting is exclusively practiced during the human activity hours from dawn to sunset and is for both eating and drinking, which differentiates the dawn to sunset intermittent fasting from the other forms of intermittent fasting where eating light meals and/or drinking are allowed during the fasting window; (2) Although the main meals are at transition time zones of the day (pre-dawn breakfast and dinner at sunset), eating and drinking outside these transition time zones is allowed as long as it is within the non-fasting window; (3) There is no interventional calorie or energy restriction; (4) Daily fasting window is in synchrony with circadian rhythm and earth’s rotation on its axis because the daily fast starts at dawn (the first transition time zone of the day) after a pre-dawn breakfast and ends at sunset (dusk) (the second transition time zone of the day) with dinner; (5) Monthly fasting window is in synchrony with the moon’s rotation around the earth and lunar phases because the monthly fasting starts and ends when the new moon is sighted (with permission from Baylor College of Medicine).

We enrolled 14 subjects with metabolic syndrome (8 males:6 females) with a mean age of 59 years (SD = 16). All subjects fasted for more than 14 h daily for 29 days beginning from May 06, 2019, until June 03, 2019 (Fig. 1). Mean FibroScan CAP was 286 (SD = 77) dB/m, and the mean elastic modulus was 9.7 (SD = 8.4) kPa. Ten subjects had moderate to severe hepatic steatosis (S2–S3), two had mild hepatic steatosis (S1), and two had no hepatic steatosis (S0). Two subjects had F4 hepatic fibrosis, two had F3 hepatic fibrosis, one had F2 hepatic fibrosis, and nine had F0–F1 hepatic fibrosis. Nine subjects were on anti-hypertensive medications; seven subjects were on antidiabetic medications, and six subjects were on statins.

The minimum required duration of daily fasting from dawn to sunset (dusk) was 14 h, 8 min for the shortest day (May 6, 2019), and 14 h, 42 min for the longest day (June 03, 2019). All subjects tolerated intermittent fasting well without any complications.

### The components of metabolic syndrome, lipid and hepatic panels, and adiposity, oxidative stress and inflammation biomarkers

Table 1 shows the mean levels of the components of metabolic syndrome, lipid panel, hepatic panel, and adiposity, oxidative stress, and inflammation biomarkers before 4-week intermittent fasting and their mean paired changes at the end of 4th week during 4-week intermittent fasting and 1 week after 4-week intermittent fasting. There was a significant reduction in weight (P < 0.0001), body mass index (P < 0.0001), waist circumference (P = 0.006), systolic (P = 0.023), diastolic (P = 0.002) and mean (P = 0.002) arterial blood pressures at the end of 4th week during 4-week intermittent fasting and a significant reduction in weight (P < 0.0001), body mass index (P < 0.0001), waist circumference (P = 0.021) and HOMA-IR (P = 0.035) 1 week after 4-week intermittent fasting compared with the levels before 4-week intermittent fasting. We observed a reduction in insulin, glucose, HOMA-IR, triglyceride, leptin, and several oxidative stress and inflammation biomarker levels and an increase in high-density lipoprotein and adiponectin levels at the end of 4^th^ week during 4-week intermittent fasting, however, these parameters did not reach statistical significance.

**Table 1:** The effect of 4-week intermittent fasting from dawn to sunset on the components of the metabolic syndrome, lipid panel, hepatic panel and adiposity, oxidative stress and inflammation biomarkers.

The components of the metabolic syndrome, lipid panel, hepatic panel and adiposity, oxidative stress and inflammation biomarkers	The levels before 4-week intermittent fasting		The levels at the end of 4th week during 4-week intermittent fasting compared with the levels before 4-week intermittent fasting			The levels one week after 4-week intermittent fasting compared with the levels at the end of 4th week during 4-week intermittent fasting			The levels one week after 4-week intermittent fasting compared with the levels before 4-week intermittent fasting		
	Mean	SD	Mean paired change^a^	SD	Paired P value	Mean paired change^b^	SD	Paired P value	Mean paired change^c^	SD	Paired P value
Weight (kg)	91.6	16.2	− 3.3	1.9	< 0.0001	0.7	1.8	0.156	− 2.5	1.4	< 0.0001
Body mass index (kg/m^2^)	31.9	3.4	− 1.1	0.6	< 0.0001	0.2	0.7	0.200	− 0.9	0.5	< 0.0001
Waist circumference (inch)	43.9	3.6	− 2.2	2.5	0.006	0.3	0.9	0.303	− 2.0	2.8	0.021
Systolic blood pressure (mmHg)	139	17	− 8	12	0.023	4	14	0.257	− 4	17	0.431
Diastolic blood pressure (mmHg)	81	10	− 8	8	0.002	6	9	0.028	− 2	7	0.275
Mean arterial pressure (mmHg)	100	11	− 8	8	0.002	5	10	0.057	− 3	10	0.333
HOMA-IR	5.0	2.7	− 0.7	1.5	0.130	0.03	1.6	0.949	− 0.6	1.0	0.035
Glucose (mg/dl)	125	28	− 6	16	0.176	3	19	0.600	− 4	15	0.396
Insulin (µU/ml)	15.9	6.6	− 2.0	5.1	0.164	0.3	4.7	0.836	− 1.7	3.5	0.084
Triglyceride (mg/dl)	165	86	− 16	73	0.413	21	51	0.146	5	43	0.699
High density lipoprotein (mg/dl)	40	13	2	6	0.277	− 1	3	0.168	1	4	0.678
Total cholesterol (mg/dl)	177	53	7	36	0.482	− 2	25	0.817	5	29	0.503
Low density lipoprotein (mg/dl)	104	42	8	32	0.376	− 3	18	0.551	4	27	0.607
Alanine aminotransferase (U/L)	21	11	– 0.1	8	0.950	− 1	4	0.583	− 1	5	0.583
Aspartate aminotransferase (U/L)	27	11	− 1	9	0.788	– 0.07	4	0.949	− 1	8	0.743
Alkaline phosphatase (U/L)	71	26	− 3	9	0.254	1	8	0.667	− 2	11	0.532
Gamma-glutamyl transferase (U/L)	29	16	− 1	7	0.466	0.4	3	0.649	− 1	6	0.526
Total bilirubin (mg/dl)	0.5	0.2	0.1	0.2	0.158	– 0.007	0.2	0.904	0.1	0.1	0.028
Albumin (g/dl)	4.2	0.4	0.2	0.3	0.092	− 0.1	0.2	0.084	0.1	0.3	0.502
Total protein (g/dl)	7.3	0.9	0.3	0.7	0.192	− 0.3	0.4	0.032	0	0.5	1.000
Leptin (pg/ml)	14,271	6821	− 1558	5429	0.303	437	2989	0.594	− 1121	4096	0.324
Adiponectin (µg/ml)	25.47	31.89	4.39	18.16	0.383	5.58	19.28	0.298	9.97	18.37	0.063
C-reactive protein (mg/L)	2.17	1.20	− 0.02	1.43	0.965	0.02	0.89	0.930	0.004	1.11	0.989
Homocysteine (µmol/l)	12.22	4.39	1.97	1.57	0.0004	− 1.34	1.17	0.001	0.63	0.97	0.029
Interleukin-1 beta (pg/ml)	3.00	1.10	− 0.07	0.62	0.684	0.08	0.33	0.395	0.01	0.46	0.946
Interleukin-6 (pg/ml)	3.89	1.48	− 0.07	0.52	0.600	0.03	0.55	0.820	− 0.04	0.47	0.757
Interleukin-8 (pg/ml)	11.88	6.98	− 0.18	2.26	0.772	2.77	6.93	0.159	2.59	7.09	0.195
Tumor necrosis factor-alpha (pg/ml)	17.25	4.59	− 1.01	3.25	0.266	1.32	2.01	0.030	0.31	2.57	0.662

^a^A positive mean paired change indicates an increase and negative mean paired change indicates a decrease in the levels measured at the end of 4th week during 4-week intermittent fasting compared with the levels measured before 4-week intermittent fasting.

^b^A positive mean paired change indicates an increase and negative mean paired change indicates a decrease in the levels measured 1 week after 4-week intermittent fasting compared with the levels measured at the end of 4th week during 4-week intermittent fasting.

^c^A positive mean paired change indicates an increase and negative mean paired change indicates a decrease in the levels measured 1 week after 4-week intermittent fasting compared with the levels measured before 4-week intermittent fasting.

### Serum proteomics

**Figure 2:**
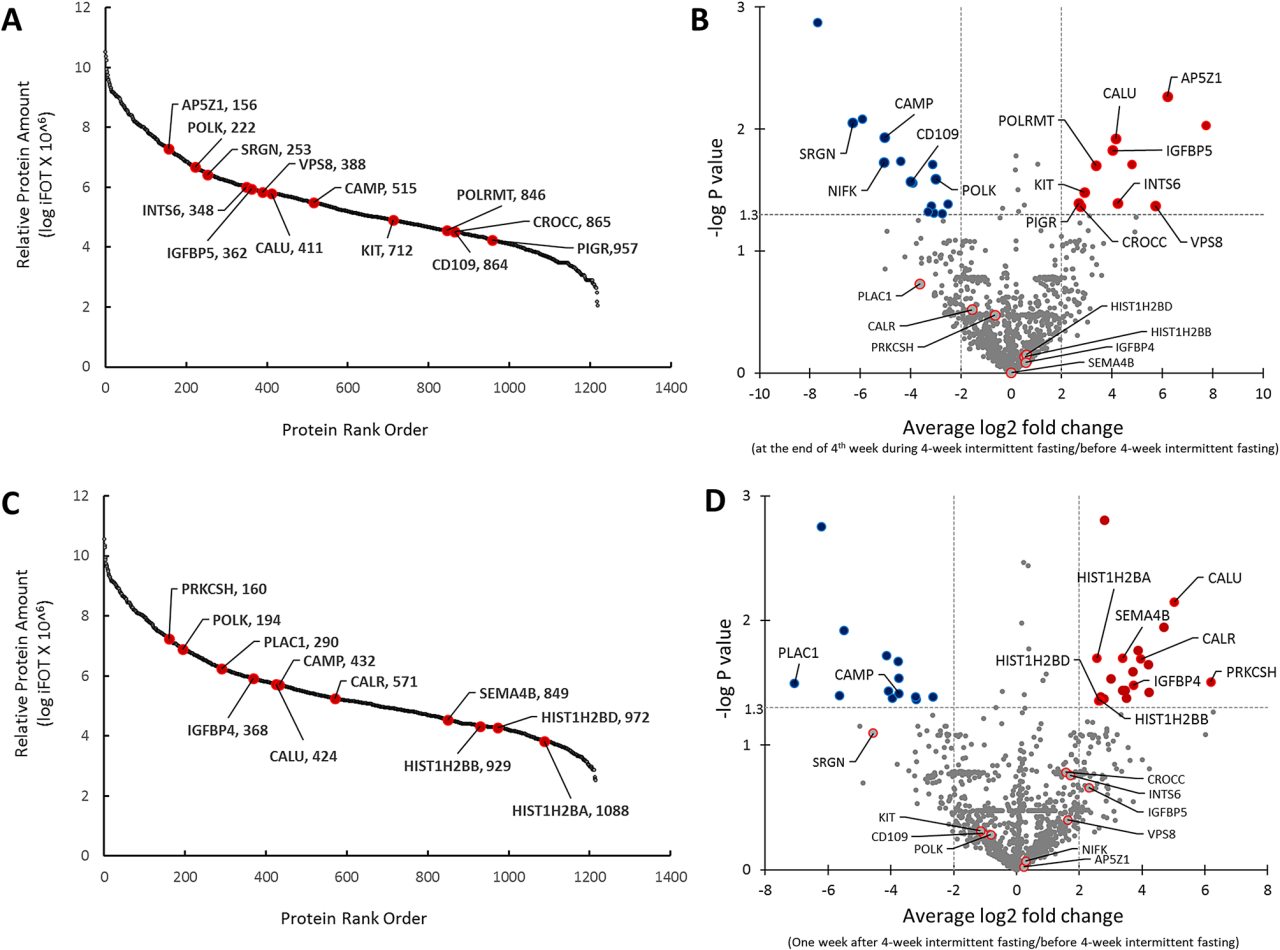
Gene protein products (GP) recovered at the end of 4th week during 4-week intermittent fasting and one week after 4-week intermittent fasting. (**A**) Distribution of normalized relative GP amount and location of focused significantly increased or decreased proteins in serum samples taken at the end of 4th week during 4-week intermittent fasting shown in GP name and rank order. (**B**) Volcano plot shows selected GPs that had an equal to or greater than fourfold significant change (blue and red colors represent a significant decrease and increase in the levels of GPs, respectively) at the end of 4th week during 4-week intermittent fasting compared with the levels before 4-week intermittent fasting. (**C**) Distribution of normalized relative GP amount and location of focused significantly increased or decreased proteins in serum samples taken 1 week after 4-week intermittent fasting shown in GP name and rank order. (**D**) Volcano plot shows selected GPs that had an equal to or greater than fourfold significant change (blue and red colors represent a significant decrease and increase in the levels of GPs, respectively) 1 week after 4-week intermittent fasting compared with the levels before 4-week intermittent fasting.

The proteome coverage and its dynamic order of average iFOT values from the samples taken at the end of 4th week during 4-week intermittent fasting are shown in Fig. 2A. There were 1219 GPs recovered with over eight orders of magnitude of dynamic range. There was a significant average paired fold change in the levels of several GPs at the end of 4th week during 4-week intermittent fasting compared with the levels before 4-week intermittent fasting (Supplementary Table S1). Figure 2A,B, and Table 2 show the selected ones from these GPs associated with tumor suppression, carcinogenesis, DNA repair, and insulin signaling. There was an average 74-fold increase in adaptor related protein complex 5 subunit zeta 1 (AP5Z1) (log2 fold = 6.201, P = 0.005), 53 fold increase in VPS8 subunit of CORVET complex (VPS8) (log2 fold = 5.730, P = 0.043), 19 fold increase in integrator complex subunit 6 INTS6 (log2 fold = 4.234, P = 0.041), 18 fold increase in calumenin (CALU) (log2 fold = 4.135, P = 0.012), 16 fold increase in insulin-like growth factor binding protein 5 (IGFBP5) (log2 fold = 4.008, P = 0.015), tenfold increase in RNA polymerase mitochondrial (POLRMT) (log2 fold = 3.355, P = 0.020), sevenfold increase in KIT proto-oncogene receptor tyrosine kinase (KIT) (log2 fold = 2.891, P = 0.033), sevenfold increase in ciliary rootlet coiled-coil, rootletin (CROCC) (log2 fold = 2.742, P = 0.043), sixfold increase in polymeric immunoglobulin receptor (PIGR) (log2 fold = 2.684, P = 0.041) GP levels at the end of 4th week during 4-week intermittent fasting compared with the levels before 4-week intermittent fasting. We found significant decrease in DNA polymerase kappa (POLK) (log2 fold = − 2.987, P = 0.026), CD109 molecule (CD109) (log2 fold = − 3.977, P = 0.027), cathelicidin antimicrobial peptide (CAMP) (log2 fold = − 5.020, P = 0.012), nucleolar protein interacting with the FHA domain of MKI67 (NIFK) (log2 fold = − 5.048, P = 0.019) and serglycin (SRGN) (log2 fold = − 6.286, P = 0.009) GP levels compared with the levels before 4-week intermittent fasting.

**Table 2:** The effect of 4-week intermittent fasting from dawn to sunset on selected gene protein products (GPs) in subjects with metabolic syndrome.

Gene symbol	Gene ID	Gene name	GP levels at the end of 4th week during 4-week intermittent fasting compared with the levels before 4-week intermittent fasting		GP levels one week after 4-week intermittent fasting compared with the levels at the end of 4th week during 4-week intermittent fasting		GP levels one week after 4-week intermittent fasting compared with the levels before 4-week intermittent fasting	
			Average paired log2 fold change^a^	Paired P value	Average paired log2 fold change^b^	Paired P value	Average paired log2 fold change^c^	Paired P value
**A. The levels of the selected GPs that are significantly upregulated at the end of 4th week during 4-week intermittent fasting compared with the levels before 4-week intermittent fasting**								
AP5Z1	9907	Adaptor related protein complex 5 subunit zeta 1	6.201	0.005	− 5.971	0.073	0.230	0.945
VPS8	23355	VPS8 subunit of CORVET complex	5.730	0.043	− 4.106	0.107	1.624	0.396
INTS6	26512	Integrator complex subunit 6	4.234	0.041	− 2.529	0.247	1.705	0.174
IGFBP5	3488	Insulin like growth factor binding protein 5	4.008	0.015	− 1.704	0.183	2.305	0.218
POLRMT	5442	RNA polymerase mitochondrial	3.355	0.020	− 3.355	0.020	ND	
KIT	3815	KIT proto-oncogene, receptor tyrosine kinase	2.891	0.033	− 4.040	0.011	− 1.149	0.484
CROCC	9696	Ciliary rootlet coiled-coil, rootletin	2.742	0.043	− 1.170	0.467	1.571	0.165
PIGR	5284	Polymeric immunoglobulin receptor	2.684	0.041	− 3.432	0.007	− 0.749	0.190
**B. The levels of the selected GPs that are significantly downregulated at the end of the 4th week during 4-week intermittent fasting compared with the levels before 4-week intermittent fasting**								
POLK	51426	DNA polymerase kappa	− 2.987	0.026	2.156	0.302	− 0.831	0.520
CD109	135228	CD109 molecule	− 3.977	0.027	2.900	0.028	− 1.076	0.512
NIFK	84365	Nucleolar protein interacting with the FHA domain of MKI67	− 5.048	0.019	5.334	0.019	0.286	0.848
SRGN	5552	serglycin	− 6.286	0.009	1.725	0.619	− 4.560	0.080
**C. The levels of the selected GPs that are significantly upregulated 1 week after 4-week intermittent fasting compared with the levels before 4-week intermittent fasting**								
PRKCSH	5589	Protein kinase C substrate 80K-H	− 0.642	0.336	6.834	0.012	6.191	0.031
CALR	811	Calreticulin	− 1.557	0.304	5.515	0.010	3.958	0.020
IGFBP4	3487	Insulin like growth factor binding protein 4	0.580	0.821	3.148	0.096	3.728	0.033
SEMA4B	10509	Semaphorin 4B	0.005	0.996	3.381	0.019	3.386	0.020
HIST1H2BB	3018	H2B clustered histone 3	0.532	0.734	2.124	0.163	2.656	0.044
HIST1H2BD	3017	H2B clustered histone 5	0.602	0.710	2.019	0.193	2.620	0.044
HIST1H2BA	255626	H2B clustered histone 1	ND		2.565	0.020	2.565	0.020
**D. The levels of the selected GP that is significantly downregulated 1 week after 4-week intermittent fasting compared with the levels before 4-week intermittent fasting**								
PLAC1	10761	Placenta enriched 1	− 3.632	0.186	− 3.439	0.082	− 7.071	0.032
**E. The levels of the selected GP that is significantly upregulated both at the end of 4th week during 4-week intermittent fasting and 1 week after 4-week intermittent fasting compared with the levels before 4-week intermittent fasting**								
CALU	813	Calumenin	4.135	0.012	0.892	0.540	5.026	0.007
**F. The levels of the selected GP that is significantly downregulated both at the end of 4th week during 4-week intermittent fasting and 1 week after 4-week intermittent fasting compared with the levels before 4-week intermittent fasting**								
CAMP	820	Cathelicidin antimicrobial peptide	− 5.020	0.012	1.277	0.559	− 3.743	0.038

*ND* Not detected.

^a^A positive average paired log2 fold change indicates an increase and negative average paired log2 fold change indicates a decrease in the levels measured at the end of 4th week during 4-week intermittent fasting compared with the levels measured before 4-week intermittent fasting.

^b^A positive average paired log2 fold change indicates an increase and negative average paired log2 fold change indicates a decrease in the levels measured 1 week after 4-week intermittent fasting compared with the levels measured at the end of 4th week during 4-week intermittent fasting.

^c^A positive average paired log2 fold change indicates an increase and negative average paired log2 fold change indicates a decrease in the levels measured 1 week after 4-week intermittent fasting compared with the levels measured before 4-week intermittent fasting.

The proteome coverage and its dynamic order from 14 samples collected 1 week after 4-week intermittent fasting are shown in Fig. 2C. There were 1216 GPs recovered with over eight orders of magnitude of dynamic range. There was a significant average paired fold change in the levels of several GPs 1 week after 4-week intermittent fasting compared with the levels before 4-week intermittent fasting (Supplementary Table S2). Figure 2C,D, and Table 2 show selected ones from these GPs associated with tumor suppression, carcinogenesis, insulin signaling, and prolonged lifespan. There was an average 73 fold increase in PRKCSH protein kinase C substrate 80 K–H (PRKCSH) (log2 fold = 6.191, P = 0.031), 33 fold increase CALU (log2 fold = 5.026, P = 0.007), 16 fold increase in calreticulin (CALR) (log2 fold = 3.958, P = 0.020), 13 fold increase in insulin-like growth factor-binding protein 4 (IGFBP4) (log2 fold = 3.728, P = 0.033), tenfold increase in semaphorin 4B (SEMA4B) (log2 fold = 3.386, P = 0.020), sixfold increase in H2B clustered histone 3 (HIST1H2BB) (log2 fold = 2.656, P = 0.044), H2B clustered histone 5 (HIST1H2BD) (log2 fold = 2.620, P = 0.044), and H2B clustered histone 1 (HIST1H2BA) (log2 fold = 2.565, P = 0.020). We found a significant reduction in cathelicidin antimicrobial peptide (CAMP) (log2 fold = − 3.743, P = 0.038) and placenta enriched 1 (PLAC1) (log2 fold = − 7.071, P = 0.032) GP levels 1 week after 4-week intermittent fasting compared with the levels before 4-week intermittent fasting.

There was a significant average paired fold change in the levels of several GPs 1 week after 4-week intermittent fasting compared with the levels at the end of 4th week during 4-week intermittent fasting (Supplementary Table S3). Supplementary Fig. 1 and Table 2 show the average paired fold changes in the selected GPs 1 week after 4-week intermittent fasting compared with the levels at the end of 4th week during 4-week intermittent fasting.

### Correlations between GPs and metabolic syndrome components, lipid and hepatic panels, adiposity, oxidative stress, and inflammation biomarkers

Several GPs that showed significant average paired fold changes at the end of 4th week during 4-week intermittent fasting and 1 week after 4-week intermittent fasting were correlated with the components of metabolic syndrome, lipid and hepatic panels and adiposity, oxidative stress and inflammation biomarkers at the end of 4th week during 4-week intermittent fasting (Supplementary Table 4) and 1 week after 4-week intermittent fasting (Supplementary Table 5). There was no significant correlation between log2 fold changes in the selected proteins (gene names are displayed in Table 2) and changes in weight, waist circumference and body mass index at the end of 4th week during 4-week intermittent fasting and 1 week after 4-week intermittent fasting compared with baseline. 

## Discussion

We reported the results of the first human study of serum proteomics of 4-week intermittent fasting from dawn to sunset conducted in subjects with metabolic syndrome. The results showed that intermittent fasting from dawn to sunset for more than 14 h daily for four consecutive weeks induced a unique anticancer, anti-diabetes and anti-aging proteomic response (Table 2, Figs. 1 and 2), upregulated several regulatory proteins that play a key role in tumor suppression, DNA repair, humoral defense, insulin signaling, and downregulated several tumor promotor proteins. These changes in protein expression are likely related to the reset of the circadian clock rhythm by intermittent fasting from dawn to sunset and in line with the results of previous murine studies^13,14^.

### Intermittent fasting from dawn to sunset for 4 weeks is associated with an anticancer serum proteomic signature

This pilot study has important clinical implications, specifically from the standpoint of type of intermittent fasting on cancer prevention in subjects with metabolic syndrome. There are two major forms of daily intermittent fasting based on the time to start and end fasting: (1) Fasting that starts at dawn and ends at sunset (dusk). The fasting window is between two symmetrical transition time zones of the day (dawn and dusk) which is the human activity period (Fig. 1); (2) Fasting that starts at a self-determined time of the day and lasts for a fixed number of hours consisting of both human activity (daytime) and inactivity (nighttime) periods (e.g., 16:8 intermittent fasting that starts at 8 pm and ends at noon).

Ramadan fasting is a unique form of intermittent fasting from dawn to sunset (dusk) without eating or drinking during the month of Ramadan based on the lunar calendar^16^ and has several major unique features: (1) Fasting is exclusively practiced during the human activity hours from dawn to sunset (dusk) and is for both eating and drinking, which differentiates the dawn to sunset intermittent fasting from the other forms of intermittent fasting where drinking and/or eating light meals are allowed during the fasting window; (2) Although the main meals are at the transition time zones of the day (pre-dawn breakfast and dinner at sunset), eating (e.g., snacks) and drinking outside these transition time zones is allowed as long as it is within the non-fasting window; (3) There is no interventional calorie or energy restriction; (4) Daily fasting window is in synchrony with circadian rhythm and earth’s rotation on its axis because the daily fast starts at dawn (the first transition time zone of the day) after a pre-dawn breakfast and ends at sunset (dusk) (the second transition time zone of the day) with dinner; (5) Monthly fasting window is in synchrony with the moon’s rotation around the earth and lunar phases because the monthly fasting starts and ends when the new moon is sighted. It is surmised that this fasting pattern results in energy reserves being accessed without leading to micronutrient deficiencies due to replenishment after sunset.

Fasting during activity hours of the day appears to be of paramount importance in cancer prevention and treatment. A study showed that mice with no access to food during the activity phase of a 12-h light/12-h dark cycle had a significantly slower tumor progression and higher survival compared with mice that had no access to food during the inactivity phase, and mice that had access to food ad libitum^13^. The highest anticancer response occurred in the mice with no access to food during the activity phase, and the worse outcome was in the mice that had access to food ad libitum^13^. There was a minor anticancer effect and no survival benefit in the mice with no access to food during the inactivity phase^13^. In regards to humans, a study conducted among 2413 women with breast cancer without diabetes mellitus showed that fasting equal to or longer than 13 h at night (the combination of inactivity and activity hours) was associated with a reduced risk of breast cancer recurrence^25^. Of note, this study did not have control subjects who fasted exclusively during the activity hours (daytime)^25^. Altogether, these animal and human studies show that intermittent fasting either during daily activity or inactivity hours (with or without extending to activity hours) have an anticancer effect compared with ad libitum eating; however, the most robust anticancer response appears to occur when prolonged fasting is practiced exclusively during the activity hours^13^.

In accord with the findings of these murine and human studies^13,25^, we found a significant fold increase in the levels of specific tumor suppressor/anticancer proteins at the end of 4th week during 4-week intermittent fasting from dawn to sunset and/or 1 week after 4-week intermittent fasting from dawn to sunset, including CALR, CALU, INTS6, KIT, CROCC, PIGR, IGFBP4, and SEMA4B that are downregulated in several cancers resulting in cancer metastasis and poor prognosis (Table 2, Fig. 2). The CALR gene encodes for calreticulin, which is a calcium-binding protein located in the endoplasmic reticulum and nucleus^26^. Cancer cells tagged by calreticulin on their surface stimulate an immunogenic cancer cell death by enabling their phagocytosis by the dendritic cells of the immune system which in turn triggers T-cell-mediated immune response^27–29^. Obeid et al*.*^28^ showed that anthracyclines translocate calreticulin to the tumor cell surface, and trigger immunogenic tumor cell death (“eat me” signal).

CALU gene encodes for calumenin, a calcium-binding protein that plays a significant role in the endoplasmic reticulum functions, including folding and sorting of proteins^26^. Calumenin is an inhibitor of cell migration and metastasis in several cancers^30^, e.g., hepatocellular^30,31^, pancreatic^30^, head and neck squamous cell^32^, and lung squamous cell^33^ carcinomas. INTS6 gene encodes for a DEAD box RNA helicase that has a motif of Asp-Glu-Ala-Asp^26^. It is a tumor suppressor gene that plays a significant tumor-suppressive role in hepatocellular carcinoma and prostate ca^34,35^. The induction of INTS6 gene was shown to suppress castration-resistant prostate cancer^35^. Mutations in KIT are linked to several cancers^26^, and the loss of proto-oncogene c-KIT expression has been associated with poor prognosis in breast cancer^36,37^.

Several other anticancer GPs require elaboration. CROCC, also known as TAX1BP2, is a tumor-suppressor gene that was shown to suppress hepatocellular carcinoma via p38/p53/p21 pathway activation^38^. The downregulation of CROCC was associated with poor survival in patients with hepatocellular carcinoma after surgical resection^38^. PIGR, that encodes for polymeric immunoglobulin receptor^26^, was found to be downregulated in pancreatic^39^ and periampullary adenocarcinoma^39^ and lung cancer^40^. The upregulation of IGFBP4 (encodes for a protein that binds insulin-like growth factors I and II^26^), was shown to function as a potent tumor suppressor in hepatocellular carcinoma and delay tumor formation in prostate cancer cells^41,42^. SEMA4B is involved in protein-coding^26^ and encodes for a protein that inhibits tumor growth in non-small cell lung cancer^43^.

A significant fold-reduction in the levels of several tumor promoter/pro-cancer GPs was observed at the end of 4th week during 4-week intermittent fasting and/or 1 week after completion of 4-week intermittent fasting. These include POLK, NIFK, SRGN, CAMP, CD109, and PLAC1 that are upregulated in several cancers resulting in metastasis and poor prognosis (Table 2, Fig. 2).

POLK gene (POLQ) encodes for a specialized DNA polymerase^26^. Specialized DNA polymerases are ectopically overexpressed in several cancers, can function as an oncogene and enhance mutations induced by DNA damage^44–46^. Overexpression of POLK has been reported in lung cancer^45,47^. POLK overexpression contributes to cancer development by inactivating wild-type p53, and this was shown in lung cancer^47^. POLK gene also plays a role in breast cancer^48^. A case–control study showed a higher risk of developing breast cancer in women with two specific single nucleotide polymorphisms in the POLK gene compared with controls^48^. NIFK that encodes for a protein that functions in mitosis and progression of cell cycle^26^ was found to be upregulated and associated with poor prognosis in lung cancer^49^. SRGN encodes for a proteoglycan in hematopoietic cells^26^ and its overexpression is associated with poor prognosis in hepatocellular^50^, colorectal cancer^51^, non-small cell lung cancer^52^, and nasopharyngeal carcinoma^53^. CAMP that is also known as LL37 and CAP18, encodes for cathelicidin antimicrobial peptide^26^. CAMP was shown to increase the growth of  colon cancer via Wnt/β-catenin signaling pathway activation^54 ^ and function as a tumor promoter for lung^55^ and ovarian^56^ cancers. CD109 encodes for a glycosyl phosphatidylinositol-linked glycoprotein^26^ that was found to be overexpressed in several tumors, e.g., malignant melanoma^57^, squamous cell carcinoma of the lung^58^ and oral cavity^59^. PLAC1, which has a biased expression in placenta^26^, was shown to be expressed in hepatocellular carcinoma^60^, and overexpressed in breast cancer^61^, non-small cell lung cancer^62^, and pancreatic ductal adenocarcinoma^63^.

### Intermittent fasting from dawn to sunset for 4 weeks can play an important role in humoral defense against severe acute respiratory syndrome‐associated coronavirus (SARS-CoV)

Calreticulin was shown to enhance IgG-mediated immune response when it is fused with spike (S) protein of SARS-CoV^64^. Recombinant fusion protein that combines calreticulin and SARS-CoV S protein 450–650 fragment had much higher immunogenicity when compared with SARS-CoV S protein alone^64^. We found an average 16-fold increase in the CALR GP level 1 week after completion of 4-week intermittent fasting compared with the level before 4-week intermittent fasting. Our findings, combined with the prior report from Qiu et al.^64^, suggest that 4-week IF can play an important role in humoral defense against SARS-CoV, and further studies are needed.

### Intermittent fasting from dawn to sunset for 4 weeks induces key regulatory proteins of insulin signaling and improves insulin resistance

Four-week intermittent fasting induced key regulatory proteins of insulin signaling, including VPS8, POLRMT and IGFBP5 at the end of 4th week during 4-week intermittent fasting and PRKCSH 1 week after 4-week intermittent fasting. The induction of VPS8, POLRMT, and IGFBP5 GPs at the end of 4th week during 4-week intermittent fasting preceded the significant reduction in insulin resistance estimated by HOMA-IR that occurred 1 week after 4-week intermittent fasting. VPS8, a subunit of CORVET complex^26^, plays a critical role in integrin requiring cell adhesion and migration, and recycling of beta-1 integrins^65^. Integrins are transmembrane receptors connecting the extracellular matrix to the actin cytoskeleton of the cells and thereby acting as a sensor for cell adhesion^66^. An impaired integrin signaling in the extracellular matrix of skeletal muscle, adipose tissue and liver can lead to insulin resistance^67^. An intact skeletal muscle and beta-cell mitochondrial function is vital for insulin synthesis. Type 2 diabetes mellitus is associated with skeletal muscle mitochondrial dysfunction and reduced oxidative capacity^68^. POLRMT encodes for the mitochondrial RNA polymerase^26^ that plays a critical role in transcription in pancreatic beta-cells and insulin secretion in the pancreas^69^. As the loss of POLRMT results in severe mitochondrial dysfunction in cardiac muscle^70^, a similar mitochondrial dysfunction should occur in beta-cells with the loss or dysfunction of POLRMT, resulting in insulin resistance and diabetes mellitus. IGFBP5 plays an active role in myoblast differentiation by binding to insulin growth factor II and upregulating its expression^71^. PRKCSH encodes for a protein called hepatocystin or 80K-H, which is a beta subunit of glucosidase II^26 ^ and substrate for protein kinase C^26^, that plays a key role in GLUT4 vesicle trafficking (translocation of GLUT4 to the plasma membrane) in the insulin signaling pathway^ 72^ by forming a complex with 80K-H Overexpression of the hepatocystin 1 week after 4-week intermittent fasting is suggestive of improvement of insulin signaling through enhancement of GLUT4 vesicle trafficking. The upregulation of PRKCSH GPs coincided with the improvement in HOMA-IR 1 week after 4-week intermittent fasting.

### Association of 4-week intermittent fasting from dawn to sunset with autophagy and oxidative stress parameters

Autophagy appears to be one of the mechanisms of intermittent fasting in cancer prevention^73^. Downregulation of the hepatocystin encoded by PRKCSH results in dysfunctional glucosidase II, and thereby increase in autophagy via mTOR dependent pathway^74^. The fact that we found reduction at the end of 4th week during 4-week intermittent fasting and then 73-fold increase in PRKCSH GP level 1 week after 4-week intermittent fasting is suggestive of increased autophagy during 4-week intermittent fasting and decreased autophagy with the subjects’ return to ad libitum eating (Table 2). Although it did not reach statistical significance, we found a reduction in multiple oxidative stress and inflammation biomarkers as well as gamma-glutamyl transferase levels, suggesting glutathione repletion at the end of 4th week during 4-week intermittent fasting (Table 1).

### Intermittent fasting from dawn to sunset for 4 weeks upregulates proteins associated with prolonged longevity and DNA repair

We observed an average sixfold increase in H2B histone GP levels 1 week after 4-week intermittent fasting compared with the levels before 4-week intermittent fasting. Feser et al*.*^75^ demonstrated similar findings in yeast cells. They reported that histone proteins are lost in aging yeast cells, whereas histone protein overexpression and supplying extra histone proteins prolonged longevity^75^. Authors suggested that extra histone protein supply extends lifespan by providing a tighter chromatin packaging, and thereby restoring the transcriptional silencing that is lost in aging^75^. We also observed a significant positive correlation between H2B histone GP levels and high-density lipoprotein levels 1 week after 4-week intermittent fasting (Supplementary Table 5). The association between high-density lipoprotein levels and longevity was previously reported^76^. Our findings of a significant positive correlation between H2B histone GP and high-density lipoprotein levels 1 week after 4-week intermittent fasting shed light on the mechanistic understanding of the association between high-density lipoprotein levels and longevity. Besides the extended life span, the overexpression of histone proteins may also be associated with increased DNA expression and repair because irradiation-induced DNA damage was shown to downregulate histone gene transcription via the G1 checkpoint pathway^77^. Additionally, we observed an average 74-fold increase in the AP5Z1 GP level at the end of 4th week during 4-week intermittent fasting compared with the level before 4-week intermittent fasting. AP5Z1 is a helicase that likely plays a role in the repair of homologous recombination DNA double-strand break^26^. A variant in the AP5Z1 gene was associated with extreme longevity in a genome-wide association study conducted among 75,000 participants of the UK biobank^78^.

### Strengths and limitations

The strengths of our study are as follows: (1) We performed a robust, streamlined proteomics method to quantify proteins in the human serum using nano UHPLC-MS/MS^15^. With this unique proteomics method, we were able to recover more than 1000 GPs at the end of the 4th week during 4-week intermittent fasting (Fig. 2A) and 1 week after 4-week intermittent fasting (Fig. 2C); (2) We evaluated serum proteome simultaneously with the components of metabolic syndrome, lipid, and hepatic panels, and adiposity, oxidative stress, and inflammation biomarkers; (3) We assessed the carryover effect of 4-week intermittent fasting after switching to ad libitum eating. For this, we compared the GP levels measured 1 week after 4-week intermittent fasting with the levels measured at the end of 4th week during 4-week intermittent fasting. Several GPs that had a significant fold change at the end of 4th week during 4-week intermittent fasting compared with the levels before 4-week intermittent fasting, did not have any significant fold change 1 week after 4-week intermittent fasting compared with the levels at the end of 4th week during 4-week intermittent fasting (e.g., CALU, CAMP) (Table 2). These findings suggest that intermittent fasting has a carryover effect on these proteins. On the other hand, several GPs that had no significant fold change at the end of 4th week during 4-week intermittent fasting compared with the levels before 4-week intermittent fasting, had a significant fold change 1 week after 4-week intermittent fasting compared with the levels at the end of 4th week during 4-week intermittent fasting (e.g., PRKCSH, HIST1H2BA) (Table 2). These findings may suggest either decreased or delayed carryover effect of intermittent fasting on the proteome. There is also a possibility that intermittent fasting might have triggered a cascade of proteomic changes in a continuum that may not be explained by the assessment of the serum proteome at a single time point alone after intermittent fasting is stopped.

Several GPs that showed significant fold changes in subjects with metabolic syndrome had also shown similar changes (e.g., increase or decrease) at the end of 4th week during 30-day intermittent fasting (KIT, CROCC, DNTT, POLK, SRGN, CLSTN1) and 1 week after 30-day intermittent fasting (PRKCSH, CALU, SPECC1L, IGFBP4, MYH7, CDH6, H2B histone, PKP1, LRRC3, FGB, ENPP2) in our previous study conducted in healthy subjects although they had not reached statistical significance^15^.

The lack of caloric measurement by dietary assessment is one of the limitations of our study. The improvement in the components of metabolic syndrome could partially be explained by the significant weight reduction. Nonetheless, we did not find any significant correlation between log2 fold changes in the selected proteins (names are displayed in Table 2) and changes in weight, waist circumference and body mass index at the end of 4th week during 4-week intermittent fasting and 1 week after 4-week intermittent fasting compared with baseline suggesting that the effect of 4-week intermittent fasting from dawn to sunset on the proteomic changes was independent of weight reduction. Furthermore, our previous study conducted in healthy volunteers who fasted from dawn to sunset for 30 days showed the induction of an anticancer proteome in the absence of a significant weight change^15^. We estimated insulin resistance by HOMA-IR equation instead of performing an oral glucose tolerance test. A non-significant reduction in HOMA-IR at the end of 4th week during 4-week intermittent fasting might be related to insufficient accuracy of the HOMA-IR equation in our study population. HOMA-IR equation was found to have limited accuracy in adults between the ages 60 and 88 and subjects on insulin treatment (unless the glucose and insulin are in steady-state levels in subjects on insulin)^79,80^. Given the fact that 11 out of 14 study subjects were 60 years old or older and one subject was on insulin treatment, HOMA-IR might have underestimated the reduction in insulin resistance that occurred at the end of 4th week during 4-week intermittent fasting. Nonetheless, there was a reduction in glucose, insulin, and HOMA-IR levels at the end of 4th week during 4-week intermittent fasting. Although these findings did not reach statistical significance, they provide additional evidence for the antidiabetic effect of the 4-week intermittent fasting from dawn to sunset.

## Conclusions

Serum proteomic signature of 4-week intermittent fasting from dawn to sunset for more than 14 h a day contributed to the mechanistic understanding of the effect of intermittent fasting from dawn to sunset on anticarcinogenesis, DNA repair, insulin signaling, humoral immunity and increased longevity in subjects with metabolic syndrome. Our findings suggest that intermittent fasting from dawn to sunset for four consecutive weeks, which is in synchrony with circadian rhythm and earth’s rotation can be an adjunct treatment in metabolic syndrome and should be tested in the prevention and treatment of metabolic syndrome-induced cancers. Altogether, our findings are in line with the results of our earlier study of 30-day dawn to sunset intermittent fasting conducted in healthy subjects^15^ and lay the foundation data for a randomized, controlled clinical trial of dawn to sunset intermittent fasting in subjects with metabolic syndrome.

## Supplementary information

Supplementary Figure 1.

Supplementary Table 1.

Supplementary Table 2.

Supplementary Table 3.

Supplementary Table 4.

Supplementary Table 5.
