# Supplementary material for: Intermittent fasting from dawn to sunset for four consecutive weeks induces anticancer serum proteome response and improves metabolic syndrome
Source: Sci Rep. 2020 Oct 27;10:18341. doi: 10.1038/s41598-020-73767-w (PMC7592042; doi:10.1038/s41598-020-73767-w)
Supplement: Supplementary file 2 — Supplementary Table 1. [file 41598_2020_73767_MOESM2_ESM.docx]

**Intermittent Fasting from Dawn to Sunset for Four Consecutive Weeks Induces Anticancer Serum Proteome Response and Improves Metabolic Syndrome**

Ayse L. Mindikoglu, M.D., M.P.H.^1, 2^; Mustafa M. Abdulsada, M.B.Ch.B.^1^; Antrix Jain, M.S.^3^; Prasun K. Jalal, M.D.^1, 2^; Sridevi Devaraj, Ph.D.^4^; Zoe R. Wilhelm, B.S.^1^, Antone R. Opekun, M.S., P.A.-C ^1, 5^, Sung Yun Jung, Ph.D.^3, 6^

**Institutions:**

1. Margaret M. and Albert B. Alkek Department of Medicine, Section of Gastroenterology and Hepatology, Baylor College of Medicine, Houston, TX
2. Michael E. DeBakey Department of Surgery, Division of Abdominal Transplantation, Baylor College of Medicine, Houston, TX
3. Advanced Technology Core, Mass Spectrometry Proteomics Core, Baylor College of Medicine, Houston, TX
4. Clinical Chemistry and Point of Care Technology, Texas Children’s Hospital and Health Centers, Department of Pathology and Immunology, Baylor College of Medicine, Houston, TX
5. Department of Pediatrics, Section of Gastroenterology, Nutrition and Hepatology, Baylor College of Medicine, Houston, TX
6. Department of Molecular & Cellular Biology, Baylor College of Medicine, Houston, TX

| **Table S1. The Levels of the Gene Protein Products (GP) that Are Up- or Downregulated at the End of 4th Week During 4-Week Intermittent Fasting (V2) Compared with the Levels Before 4-Week Intermittent Fasting (V1)** | | | |
| --- | --- | --- | --- |
| **Gene Symbol** | **Gene ID** | **Average Paired Log2 Fold (V2/V1) Change** | **Paired P Value** |
| ODF2 | 4957 | -7.695 | 0.0013 |
| AP5Z1 | 9907 | 6.201 | 0.0054 |
| FAM217A | 222826 | -5.923 | 0.0082 |
| SRGN | 5552 | -6.286 | 0.0089 |
| KRT27 | 342574 | 7.715 | 0.0092 |
| CAMP | 820 | -5.020 | 0.0117 |
| CALU | 813 | 4.135 | 0.0120 |
| IGFBP5 | 3488 | 4.008 | 0.0149 |
| CFH | 3075 | 0.186 | 0.0166 |
| STBD1 | 8987 | -4.401 | 0.0181 |
| NIFK | 84365 | -5.048 | 0.0188 |
| CLSTN1 | 22883 | -3.128 | 0.0193 |
| FN1 | 2335 | 1.045 | 0.0193 |
| DNTT | 1791 | 4.772 | 0.0193 |
| POLRMT | 5442 | 3.355 | 0.0199 |
| ITIH4 | 3700 | 0.165 | 0.0216 |
| POLK | 51426 | -2.987 | 0.0256 |
| CD109 | 135228 | -3.977 | 0.0271 |
| PLEKHG1 | 57480 | -3.923 | 0.0277 |
| APOA2 | 336 | 0.275 | 0.0313 |
| KIT | 3815 | 2.891 | 0.0331 |
| PIGR | 5284 | 2.684 | 0.0406 |
| INTS6 | 26512 | 4.234 | 0.0406 |
| ARFGEF3 | 57221 | -2.540 | 0.0409 |
| SHBG | 6462 | 0.526 | 0.0421 |
| VPS8 | 23355 | 5.730 | 0.0425 |
| ARHGDIA | 396 | -3.201 | 0.0426 |
| CROCC | 9696 | 2.742 | 0.0431 |
| MASP1 | 5648 | -0.345 | 0.0444 |
| MST1 | 4485 | 0.275 | 0.0472 |
| SIGLEC5 | 8778 | -3.323 | 0.0473 |
| CASP4 | 837 | -3.082 | 0.0485 |
| CNTN3 | 5067 | -2.754 | 0.0494 |
| SELENBP1 | 8991 | -2.844 | 0.0506 |
| TBC1D30 | 23329 | 3.117 | 0.0516 |
| TCERG1 | 10915 | 4.960 | 0.0527 |
| SPARCL1 | 8404 | -0.446 | 0.0532 |
| HAUS1 | 115106 | -3.702 | 0.0563 |
| C1QTNF3 | 114899 | -2.706 | 0.0565 |
| UGT8 | 7368 | 2.727 | 0.0590 |
| MGP | 4256 | 3.060 | 0.0681 |
| CRTAC1 | 55118 | 1.164 | 0.0683 |
| CA2 | 760 | -4.939 | 0.0686 |
| HOMER2 | 9455 | 4.897 | 0.0693 |
| EYS | 346007 | 1.986 | 0.0758 |
| BCL10 | 8915 | -3.586 | 0.0784 |
| SLC4A10 | 57282 | -2.224 | 0.0813 |
| STIM1 | 6786 | 2.709 | 0.0816 |
| ADGRL4 | 64123 | -1.830 | 0.0824 |
| PAM | 5066 | -1.795 | 0.0827 |
| GC | 2638 | 0.198 | 0.0827 |
| PDZD3 | 79849 | -2.463 | 0.0827 |
| EXOSC8 | 11340 | -2.498 | 0.0829 |
| RUNDC3A | 10900 | 2.337 | 0.0830 |
| IGF2 | 3481 | 2.521 | 0.0830 |
| L1CAM | 3897 | -1.475 | 0.0831 |
| LOC101927506 | 101927506 | 2.643 | 0.0834 |
| RAD50 | 10111 | -1.642 | 0.0836 |
| UBE2V2 | 7336 | -1.932 | 0.0837 |
| FSTL1 | 11167 | -1.847 | 0.0838 |
| LZTS3 | 9762 | 2.568 | 0.0840 |
| NCOA7 | 135112 | -2.672 | 0.0857 |
| RUFY3 | 22902 | 2.185 | 0.0863 |
| PTER | 9317 | -3.768 | 0.0865 |
| PNRC1 | 10957 | -2.570 | 0.0866 |
| RASL11A | 387496 | 3.663 | 0.0867 |
| KIAA1217 | 56243 | 2.586 | 0.0876 |
| MYOC | 4653 | 2.029 | 0.0886 |
| ANKRD62 | 342850 | 2.901 | 0.0894 |
| DSC1 | 1823 | -1.943 | 0.0894 |
| HSPA1L | 3305 | 2.046 | 0.0909 |
| BPTF | 2186 | 1.620 | 0.0912 |
| C19orf54 | 284325 | 3.235 | 0.0923 |
| B3GAT3 | 26229 | 2.620 | 0.0941 |
| PCF11 | 51585 | -1.562 | 0.0945 |
| APOD | 347 | 0.187 | 0.0965 |
| PGK2 | 5232 | 2.873 | 0.0978 |
| MORC1 | 27136 | 2.463 | 0.0991 |
| MYH7B | 57644 | -1.023 | 0.0992 |
| HSPA6 | 3310 | 2.827 | 0.0998 |
| RMND1 | 55005 | -2.299 | 0.1013 |
| ICOSLG | 23308 | -2.083 | 0.1034 |
| LOC102723996 | 102723996 | -2.031 | 0.1041 |
| LDHA | 3939 | -3.024 | 0.1056 |
| ANG | 283 | -2.786 | 0.1080 |
| COL6A1 | 1291 | -2.823 | 0.1102 |
| CNTLN | 54875 | -2.431 | 0.1117 |
| SCLT1 | 132320 | -4.232 | 0.1117 |
| TMCO5A | 145942 | 2.196 | 0.1129 |
| CGNL1 | 84952 | 3.194 | 0.1136 |
| SLC22A6 | 9356 | -3.562 | 0.1142 |
| EFCAB6 | 64800 | -3.257 | 0.1142 |
| ANGPTL3 | 27329 | -2.804 | 0.1149 |
| SELP | 6403 | -2.376 | 0.1169 |
| LRIG1 | 26018 | 1.746 | 0.1188 |
| MYO18B | 84700 | -3.269 | 0.1194 |
| ITGA8 | 8516 | -2.990 | 0.1213 |
| CACNA2D1 | 781 | -1.599 | 0.1242 |
| ZHX2 | 22882 | -3.579 | 0.1256 |
| ING1 | 3621 | -2.614 | 0.1276 |
| DHX8 | 1659 | 3.380 | 0.1293 |
| LTF | 4057 | 1.743 | 0.1304 |
| PEPD | 5184 | -1.979 | 0.1317 |
| PLTP | 5360 | -0.242 | 0.1327 |
| QSOX1 | 5768 | -0.207 | 0.1340 |
| MED14 | 9282 | -1.822 | 0.1379 |
| BASP1 | 10409 | 2.450 | 0.1395 |
| TGM3 | 7053 | 2.412 | 0.1407 |
| ARFIP1 | 27236 | -5.005 | 0.1407 |
| ARHGAP22 | 58504 | 2.167 | 0.1411 |
| LOC107983983 | 107983983 | -1.665 | 0.1428 |
| LOC102724971 | 102724971 | -1.743 | 0.1437 |
| DSC3 | 1825 | -1.847 | 0.1457 |
| LTA4H | 4048 | -2.418 | 0.1476 |
| GPX3 | 2878 | -0.217 | 0.1515 |
| APOC4 | 346 | -0.284 | 0.1526 |
| DAG1 | 1605 | -3.000 | 0.1543 |
| C8B | 732 | -0.162 | 0.1548 |
| AMY1A | 276 | -1.903 | 0.1550 |
| AMY1B | 277 | -1.903 | 0.1550 |
| AMY1C | 278 | -1.903 | 0.1550 |
| AMY2A | 279 | -1.903 | 0.1550 |
| AMY2B | 280 | -1.903 | 0.1550 |
| FMN2 | 56776 | 3.212 | 0.1556 |
| IKBKB | 3551 | 3.598 | 0.1584 |
| FYCO1 | 79443 | -1.859 | 0.1611 |
| MYO5B | 4645 | 1.631 | 0.1620 |
| CPS1 | 1373 | -1.623 | 0.1642 |
| SORCS2 | 57537 | 1.641 | 0.1645 |
| OTUD6A | 139562 | -2.050 | 0.1648 |
| SLC5A9 | 200010 | 1.534 | 0.1648 |
| ACTG2 | 72 | -2.174 | 0.1648 |
| ACTA2 | 59 | -2.159 | 0.1648 |
| P4HTM | 54681 | 1.342 | 0.1648 |
| CALD1 | 800 | -1.179 | 0.1648 |
| RIMS2 | 9699 | 1.339 | 0.1648 |
| PRG2 | 5553 | -1.486 | 0.1648 |
| AOC2 | 314 | -1.101 | 0.1648 |
| LMO2 | 4005 | -2.149 | 0.1649 |
| VASP | 7408 | 1.289 | 0.1649 |
| SYTL2 | 54843 | -1.346 | 0.1649 |
| ZP4 | 57829 | 1.471 | 0.1649 |
| CARD8 | 22900 | 1.130 | 0.1649 |
| YWHAE | 7531 | -1.761 | 0.1649 |
| CAP1 | 10487 | -1.312 | 0.1649 |
| PSMA7 | 5688 | -1.475 | 0.1649 |
| GOT1 | 2805 | -1.482 | 0.1649 |
| STIP1 | 10963 | -1.149 | 0.1649 |
| TALDO1 | 6888 | -1.547 | 0.1649 |
| EME1 | 146956 | 1.487 | 0.1650 |
| E2F5 | 1875 | 1.735 | 0.1650 |
| PTPMT1 | 114971 | 1.577 | 0.1650 |
| CFL2 | 1073 | 1.467 | 0.1650 |
| KRT7 | 3855 | -1.848 | 0.1650 |
| CCL14 | 6358 | 1.987 | 0.1650 |
| FAM214A | 56204 | 1.140 | 0.1650 |
| TMEM131L | 23240 | -1.064 | 0.1650 |
| TNS4 | 84951 | 1.508 | 0.1650 |
| DIS3 | 22894 | 1.266 | 0.1651 |
| MYBL1 | 4603 | 1.717 | 0.1651 |
| CDHR5 | 53841 | 1.342 | 0.1651 |
| FOCAD | 54914 | -1.208 | 0.1651 |
| GALNT2 | 2590 | -1.517 | 0.1651 |
| CNTN4 | 152330 | -1.139 | 0.1651 |
| PPFIA3 | 8541 | 1.663 | 0.1651 |
| ADGRF4 | 221393 | 1.493 | 0.1651 |
| HSP90AB1 | 3326 | -1.269 | 0.1652 |
| ICK | 22858 | 1.802 | 0.1652 |
| SYT5 | 6861 | -1.450 | 0.1652 |
| TIAM2 | 26230 | 1.486 | 0.1654 |
| LDHB | 3945 | -0.375 | 0.1654 |
| SOD2 | 6648 | -1.571 | 0.1656 |
| CSAD | 51380 | 1.593 | 0.1657 |
| DSE | 29940 | -1.223 | 0.1659 |
| GDI1 | 2664 | -1.255 | 0.1659 |
| GDI2 | 2665 | -1.252 | 0.1659 |
| GSPT2 | 23708 | 2.109 | 0.1660 |
| APOA4 | 337 | -0.173 | 0.1660 |
| FCN1 | 2219 | -1.385 | 0.1660 |
| CNTNAP5 | 129684 | -1.547 | 0.1660 |
| FDFT1 | 2222 | 2.463 | 0.1663 |
| IL21 | 59067 | -2.176 | 0.1668 |
| TUBB2A | 7280 | 1.325 | 0.1672 |
| RUNX1T1 | 862 | 0.974 | 0.1673 |
| TUBB2B | 347733 | 1.307 | 0.1673 |
| CBFA2T2 | 9139 | 0.957 | 0.1673 |
| CBFA2T3 | 863 | 0.955 | 0.1674 |
| NCAM2 | 4685 | -1.104 | 0.1674 |
| F13B | 2165 | -1.392 | 0.1677 |
| LECT2 | 3950 | 1.783 | 0.1679 |
| ZNF479 | 90827 | -1.859 | 0.1680 |
| CDH8 | 1006 | 1.437 | 0.1682 |
| PLXDC1 | 57125 | -1.585 | 0.1683 |
| TUBB8 | 347688 | 1.316 | 0.1684 |
| TUBB4A | 10382 | 1.277 | 0.1687 |
| TUBB4B | 10383 | 1.277 | 0.1687 |
| NRCAM | 4897 | -1.523 | 0.1688 |
| INTS7 | 25896 | -1.445 | 0.1690 |
| CEP126 | 57562 | 1.434 | 0.1690 |
| SERPINB4 | 6318 | 1.508 | 0.1693 |
| SERPINB3 | 6317 | 1.495 | 0.1694 |
| MYO1A | 4640 | -1.721 | 0.1702 |
| SEL1L3 | 23231 | 1.578 | 0.1708 |
| KIAA0100 | 9703 | -0.965 | 0.1712 |
| TJP2 | 9414 | 1.937 | 0.1714 |
| ACY1 | 95 | 1.080 | 0.1714 |
| ICAM1 | 3383 | -2.213 | 0.1716 |
| NCF2 | 4688 | 1.679 | 0.1718 |
| ABHD14A-ACY1 | 100526760 | 1.009 | 0.1724 |
| ANXA1 | 301 | 1.384 | 0.1727 |
| ESF1 | 51575 | -1.508 | 0.1732 |
| CFAP58 | 159686 | 1.770 | 0.1735 |
| PECAM1 | 5175 | -1.572 | 0.1740 |
| HIST1H2BJ | 8970 | -1.246 | 0.1742 |
| H2BFS | 54145 | -1.246 | 0.1742 |
| HIST1H2BK | 85236 | -1.246 | 0.1742 |
| LOC102724334 | 102724334 | -1.246 | 0.1742 |
| TUBB | 203068 | 1.860 | 0.1748 |
| SAA1 | 6288 | -1.545 | 0.1754 |
| GPR173 | 54328 | -2.025 | 0.1793 |
| ITGA2 | 3673 | -1.033 | 0.1794 |
| CRISP2 | 7180 | -1.335 | 0.1816 |
| MYH14 | 79784 | -1.343 | 0.1822 |
| CUL1 | 8454 | 1.179 | 0.1824 |
| CASP14 | 23581 | 1.848 | 0.1836 |
| PLAC1 | 10761 | -3.632 | 0.1856 |
| CDH13 | 1012 | 0.198 | 0.1864 |
| DDAH1 | 23576 | 1.283 | 0.1867 |
| TUB | 7275 | 2.560 | 0.1873 |
| CKAP4 | 10970 | -0.907 | 0.1879 |
| MYH6 | 4624 | 1.639 | 0.1885 |
| RC3H2 | 54542 | -2.199 | 0.1900 |
| TREML1 | 340205 | -2.761 | 0.1927 |
| SELENOP | 6414 | -0.194 | 0.1933 |
| SLC4A9 | 83697 | 1.369 | 0.1951 |
| PLXNB2 | 23654 | -1.770 | 0.1976 |
| TMF1 | 7110 | 2.124 | 0.2007 |
| QSER1 | 79832 | 2.177 | 0.2040 |
| SNED1 | 25992 | -1.732 | 0.2062 |
| IARS | 3376 | 1.653 | 0.2085 |
| SLC27A5 | 10998 | 1.493 | 0.2086 |
| CGAS | 115004 | 2.395 | 0.2102 |
| TKT | 7086 | -1.997 | 0.2116 |
| ROCK2 | 9475 | 2.364 | 0.2120 |
| DPP4 | 1803 | -1.523 | 0.2135 |
| PPL | 5493 | 2.649 | 0.2136 |
| CRISP3 | 10321 | 2.287 | 0.2152 |
| LYZ | 4069 | 0.212 | 0.2154 |
| TRAM1 | 23471 | 2.116 | 0.2172 |
| CEP152 | 22995 | 2.843 | 0.2185 |
| ADCY10 | 55811 | 1.759 | 0.2188 |
| HLA-C | 3107 | -3.027 | 0.2193 |
| TNXB | 7148 | -0.460 | 0.2196 |
| DCDC2B | 149069 | -2.788 | 0.2198 |
| SYNM | 23336 | -3.051 | 0.2233 |
| TGOLN2 | 10618 | 1.722 | 0.2242 |
| TSNARE1 | 203062 | 2.932 | 0.2259 |
| LOC102723407 | 102723407 | -3.007 | 0.2288 |
| EXTL2 | 2135 | -2.211 | 0.2299 |
| ITGA2B | 3674 | 1.321 | 0.2310 |
| HMOX2 | 3163 | -3.054 | 0.2321 |
| MAN2A1 | 4124 | 1.845 | 0.2355 |
| SLC44A3 | 126969 | 0.974 | 0.2358 |
| S100A8 | 6279 | 0.414 | 0.2369 |
| ITGB3 | 3690 | -0.191 | 0.2373 |
| LBP | 3929 | -0.227 | 0.2376 |
| C1S | 716 | -0.122 | 0.2388 |
| TIMP2 | 7077 | -2.106 | 0.2389 |
| NEO1 | 4756 | -0.981 | 0.2399 |
| SERPINA11 | 256394 | 0.367 | 0.2402 |
| SERPING1 | 710 | 0.082 | 0.2424 |
| ZFYVE16 | 9765 | 2.868 | 0.2427 |
| PRR11 | 55771 | -0.346 | 0.2434 |
| PI16 | 221476 | 0.155 | 0.2434 |
| TPI1 | 7167 | -2.572 | 0.2444 |
| CD163 | 9332 | -1.764 | 0.2450 |
| MYO1F | 4542 | 2.847 | 0.2455 |
| DNAAF2 | 55172 | -1.620 | 0.2456 |
| CDH2 | 1000 | -2.225 | 0.2457 |
| BLVRB | 645 | -2.418 | 0.2477 |
| MICA | 100507436 | 2.257 | 0.2482 |
| LYVE1 | 10894 | -1.820 | 0.2486 |
| GBGT1 | 26301 | 2.495 | 0.2502 |
| ALDOB | 229 | -0.493 | 0.2548 |
| COL4A3BP | 10087 | -2.320 | 0.2550 |
| LRRC3 | 81543 | 1.439 | 0.2561 |
| NEDD8-MDP1 | 100528064 | 1.053 | 0.2571 |
| CCS | 9973 | -0.865 | 0.2582 |
| ALCAM | 214 | 2.524 | 0.2585 |
| PROZ | 8858 | -0.303 | 0.2590 |
| MDP1 | 145553 | 1.086 | 0.2594 |
| C1QA | 712 | -0.313 | 0.2616 |
| DLEC1 | 9940 | -2.454 | 0.2631 |
| RARRES2 | 5919 | 2.599 | 0.2633 |
| CHL1 | 10752 | -0.185 | 0.2639 |
| ECM1 | 1893 | 0.371 | 0.2639 |
| IFIT2 | 3433 | -2.413 | 0.2639 |
| MGAT1 | 4245 | 0.738 | 0.2650 |
| MMRN1 | 22915 | 0.967 | 0.2650 |
| CAT | 847 | -2.190 | 0.2674 |
| IFIT5 | 24138 | -2.262 | 0.2689 |
| COG4 | 25839 | 1.958 | 0.2699 |
| NFE2L2 | 4780 | 2.693 | 0.2703 |
| OLFM1 | 10439 | 1.095 | 0.2711 |
| IFIT1 | 3434 | -2.199 | 0.2711 |
| NIPSNAP1 | 8508 | -1.410 | 0.2713 |
| VTN | 7448 | -0.139 | 0.2717 |
| GSTO1 | 9446 | -2.029 | 0.2730 |
| CEP68 | 23177 | -2.422 | 0.2735 |
| HYI | 81888 | 0.359 | 0.2748 |
| ADAM30 | 11085 | -1.363 | 0.2748 |
| CD44 | 960 | -0.946 | 0.2762 |
| CENPF | 1063 | 2.624 | 0.2767 |
| ZNF544 | 27300 | -1.947 | 0.2787 |
| SAA2 | 6289 | -2.632 | 0.2806 |
| ART3 | 419 | 1.060 | 0.2822 |
| C8A | 731 | -0.114 | 0.2829 |
| CST3 | 1471 | 0.165 | 0.2855 |
| NAGLU | 4669 | 2.153 | 0.2871 |
| XRCC5 | 7520 | -0.911 | 0.2890 |
| KRT78 | 196374 | 1.710 | 0.2925 |
| TAF1 | 6872 | 1.520 | 0.2926 |
| DNAH6 | 1768 | 0.780 | 0.2928 |
| IGFBP6 | 3489 | -0.214 | 0.2949 |
| C8G | 733 | -0.093 | 0.2957 |
| PRELID2 | 153768 | -1.892 | 0.2960 |
| CFHR1 | 3078 | 3.136 | 0.2964 |
| RNH1 | 6050 | -2.465 | 0.3007 |
| CYP27B1 | 1594 | 0.852 | 0.3015 |
| CALR | 811 | -1.557 | 0.3035 |
| MYRF | 745 | -1.749 | 0.3046 |
| APOC2 | 344 | -0.349 | 0.3069 |
| FBLN1 | 2192 | -0.161 | 0.3096 |
| SERPINA3 | 12 | 0.093 | 0.3097 |
| PPIB | 5479 | -0.790 | 0.3121 |
| ZPR1 | 8882 | -1.885 | 0.3123 |
| EPHA4 | 2043 | 1.120 | 0.3131 |
| XIRP2 | 129446 | 2.704 | 0.3139 |
| LYSMD3 | 116068 | -2.555 | 0.3144 |
| FBXO41 | 150726 | -2.353 | 0.3146 |
| PPIA | 5478 | -2.493 | 0.3174 |
| HERC2 | 8924 | 1.424 | 0.3176 |
| RANBP2 | 5903 | -2.129 | 0.3179 |
| ARHGEF2 | 9181 | -2.632 | 0.3179 |
| CDHR2 | 54825 | 1.214 | 0.3198 |
| PKLR | 5313 | -1.416 | 0.3228 |
| VCL | 7414 | -1.962 | 0.3229 |
| LEKR1 | 389170 | -1.582 | 0.3230 |
| TMSB10 | 9168 | -0.975 | 0.3240 |
| PHF21A | 51317 | 1.148 | 0.3246 |
| SERPINF1 | 5176 | -0.102 | 0.3246 |
| KLHDC7A | 127707 | 1.295 | 0.3252 |
| VIT | 5212 | 1.025 | 0.3255 |
| CRP | 1401 | -1.253 | 0.3258 |
| PEBP4 | 157310 | -2.661 | 0.3269 |
| ARR3 | 407 | 2.204 | 0.3272 |
| ZNF425 | 155054 | 1.420 | 0.3276 |
| CTSC | 1075 | 0.698 | 0.3284 |
| PRAP1 | 118471 | -1.617 | 0.3289 |
| PDZD2 | 23037 | -2.634 | 0.3291 |
| DSG2 | 1829 | 1.611 | 0.3294 |
| PCSK9 | 255738 | -1.052 | 0.3312 |
| CHI3L1 | 1116 | 0.980 | 0.3315 |
| CA1 | 759 | -0.650 | 0.3319 |
| C5 | 727 | -0.088 | 0.3322 |
| PF4V1 | 5197 | 0.388 | 0.3340 |
| PF4 | 5196 | 0.388 | 0.3340 |
| SRPRA | 6734 | -1.925 | 0.3342 |
| GOPC | 57120 | -2.283 | 0.3343 |
| MERTK | 10461 | 1.374 | 0.3349 |
| KRT19 | 3880 | -1.430 | 0.3355 |
| SAA4 | 6291 | -0.007 | 0.3356 |
| HIPK1 | 204851 | 0.089 | 0.3356 |
| PHF21B | 112885 | 0.202 | 0.3356 |
| TACC1 | 6867 | -0.510 | 0.3356 |
| SARDH | 1757 | 0.549 | 0.3356 |
| HLA-DPB1 | 3115 | -0.488 | 0.3356 |
| MYH2 | 4620 | -0.367 | 0.3356 |
| SIRPB1 | 10326 | -0.536 | 0.3356 |
| MAST2 | 23139 | 0.615 | 0.3356 |
| SVEP1 | 79987 | 0.390 | 0.3356 |
| CTSG | 1511 | 0.664 | 0.3356 |
| ARHGAP35 | 2909 | -0.673 | 0.3356 |
| LDLR | 3949 | -0.534 | 0.3356 |
| TRPS1 | 7227 | 0.513 | 0.3356 |
| PARP2 | 10038 | 0.439 | 0.3356 |
| WDFY3 | 23001 | -0.375 | 0.3356 |
| C9orf40 | 55071 | -0.653 | 0.3356 |
| OTUD7A | 161725 | -0.559 | 0.3356 |
| PIWIL3 | 440822 | 0.843 | 0.3356 |
| PRKCSH | 5589 | -0.642 | 0.3356 |
| ABCB7 | 22 | -0.600 | 0.3356 |
| ACP1 | 52 | 0.701 | 0.3356 |
| RND3 | 390 | -0.636 | 0.3356 |
| EGFR | 1956 | -0.686 | 0.3356 |
| EXT2 | 2132 | 0.730 | 0.3356 |
| SFN | 2810 | 0.939 | 0.3356 |
| GPT | 2875 | -0.877 | 0.3356 |
| GTF2E1 | 2960 | 0.841 | 0.3356 |
| HIST1H1D | 3007 | 0.872 | 0.3356 |
| HIST1H1B | 3009 | 0.872 | 0.3356 |
| HLA-DQB1 | 3119 | -0.486 | 0.3356 |
| HLA-DRB1 | 3123 | -0.486 | 0.3356 |
| HLA-E | 3133 | -0.585 | 0.3356 |
| HLA-G | 3135 | -0.797 | 0.3356 |
| HSPA1A | 3303 | 0.511 | 0.3356 |
| HSPA1B | 3304 | 0.511 | 0.3356 |
| IDH3A | 3419 | 0.766 | 0.3356 |
| JAK1 | 3716 | -0.710 | 0.3356 |
| MAK | 4117 | -0.645 | 0.3356 |
| MYL6 | 4637 | -0.694 | 0.3356 |
| PPID | 5481 | 0.788 | 0.3356 |
| PRKAB2 | 5565 | 0.628 | 0.3356 |
| RELN | 5649 | -0.575 | 0.3356 |
| RPA1 | 6117 | 1.002 | 0.3356 |
| SHH | 6469 | -0.629 | 0.3356 |
| SLC2A3 | 6515 | 0.767 | 0.3356 |
| SMARCA4 | 6597 | 0.789 | 0.3356 |
| SREBF2 | 6721 | 1.039 | 0.3356 |
| THOP1 | 7064 | -0.572 | 0.3356 |
| SCGB1A1 | 7356 | 0.916 | 0.3356 |
| SYMPK | 8189 | 0.669 | 0.3356 |
| FKBP6 | 8468 | -0.806 | 0.3356 |
| MYH13 | 8735 | 0.579 | 0.3356 |
| ASAP2 | 8853 | -0.572 | 0.3356 |
| DGKI | 9162 | 0.796 | 0.3356 |
| STARD8 | 9754 | -0.730 | 0.3356 |
| SLC22A7 | 10864 | -0.561 | 0.3356 |
| C11orf58 | 10944 | 0.779 | 0.3356 |
| TMCC1 | 23023 | 0.658 | 0.3356 |
| KHNYN | 23351 | 0.702 | 0.3356 |
| DROSHA | 29102 | -0.614 | 0.3356 |
| LMCD1 | 29995 | -0.886 | 0.3356 |
| PI4K2A | 55361 | -0.847 | 0.3356 |
| PRDM9 | 56979 | 0.722 | 0.3356 |
| SLC12A5 | 57468 | 0.402 | 0.3356 |
| QTRT2 | 79691 | 1.111 | 0.3356 |
| PUS3 | 83480 | 0.771 | 0.3356 |
| NAA11 | 84779 | 0.847 | 0.3356 |
| N4BP2L1 | 90634 | -0.837 | 0.3356 |
| ZNF697 | 90874 | 0.692 | 0.3356 |
| TTC30A | 92104 | 0.682 | 0.3356 |
| SPATA17 | 128153 | -0.892 | 0.3356 |
| SIRPA | 140885 | -0.512 | 0.3356 |
| SPATA45 | 149643 | 0.773 | 0.3356 |
| RIBC1 | 158787 | -0.884 | 0.3356 |
| NOP9 | 161424 | -0.686 | 0.3356 |
| VMO1 | 284013 | -0.720 | 0.3356 |
| TTLL6 | 284076 | -0.797 | 0.3356 |
| NCCRP1 | 342897 | 0.680 | 0.3356 |
| AGRN | 375790 | 0.441 | 0.3356 |
| SERPINA2 | 390502 | -0.725 | 0.3356 |
| C6orf201 | 404220 | 0.909 | 0.3356 |
| POTEF | 728378 | -1.008 | 0.3356 |
| LOC105369914 | 105369914 | -0.887 | 0.3356 |
| LOC105371566 | 105371566 | 0.838 | 0.3356 |
| LOC105377021 | 105377021 | 0.823 | 0.3356 |
| LOC107987423 | 107987423 | -0.788 | 0.3356 |
| ALDH7A1 | 501 | 0.979 | 0.3356 |
| EN1 | 2019 | 1.131 | 0.3356 |
| MTOR | 2475 | -0.628 | 0.3356 |
| HIST1H1A | 3024 | 0.905 | 0.3356 |
| IDH1 | 3417 | -0.592 | 0.3356 |
| KIFC3 | 3801 | -0.929 | 0.3356 |
| MDH1 | 4190 | 0.831 | 0.3356 |
| MMP11 | 4320 | -0.715 | 0.3356 |
| PDGFRB | 5159 | -0.592 | 0.3356 |
| CTSA | 5476 | 0.761 | 0.3356 |
| PRKAR2B | 5577 | 0.844 | 0.3356 |
| S100A7 | 6278 | 0.906 | 0.3356 |
| SPP1 | 6696 | 0.910 | 0.3356 |
| TSTA3 | 7264 | 0.717 | 0.3356 |
| YWHAB | 7529 | 0.909 | 0.3356 |
| AXIN2 | 8313 | -0.995 | 0.3356 |
| CPNE1 | 8904 | 0.722 | 0.3356 |
| COX5A | 9377 | -0.911 | 0.3356 |
| AQR | 9716 | -0.538 | 0.3356 |
| C2CD5 | 9847 | -0.765 | 0.3356 |
| SLC12A6 | 9990 | 0.405 | 0.3356 |
| DNM1L | 10059 | 0.724 | 0.3356 |
| SEMA4F | 10505 | 0.779 | 0.3356 |
| PDLIM5 | 10611 | 0.927 | 0.3356 |
| HSPH1 | 10808 | 0.842 | 0.3356 |
| CLPX | 10845 | -0.804 | 0.3356 |
| CORO1C | 23603 | 0.738 | 0.3356 |
| NECTIN3 | 25945 | 0.925 | 0.3356 |
| FGFR1OP2 | 26127 | 0.863 | 0.3356 |
| AKAP8L | 26993 | 0.894 | 0.3356 |
| ST6GALNAC4 | 27090 | -1.070 | 0.3356 |
| GP6 | 51206 | -0.705 | 0.3356 |
| ELOA2 | 51224 | -0.794 | 0.3356 |
| CWC25 | 54883 | 0.830 | 0.3356 |
| ACSM5 | 54988 | 0.873 | 0.3356 |
| FANCI | 55215 | -0.771 | 0.3356 |
| FAR2 | 55711 | 0.993 | 0.3356 |
| CTNNBIP1 | 56998 | 1.221 | 0.3356 |
| PITPNM2 | 57605 | 0.685 | 0.3356 |
| CTAGE1 | 64693 | 0.809 | 0.3356 |
| ALG13 | 79868 | -0.981 | 0.3356 |
| NUDT18 | 79873 | -0.888 | 0.3356 |
| NUF2 | 83540 | 0.940 | 0.3356 |
| USP26 | 83844 | -0.930 | 0.3356 |
| TMEM117 | 84216 | -0.927 | 0.3356 |
| FIBCD1 | 84929 | -0.841 | 0.3356 |
| PHLDB2 | 90102 | -0.545 | 0.3356 |
| SPIC | 121599 | -0.939 | 0.3356 |
| PLD4 | 122618 | -0.899 | 0.3356 |
| SLC2A14 | 144195 | 0.764 | 0.3356 |
| RNF217 | 154214 | -1.082 | 0.3356 |
| ELOA3 | 162699 | -0.845 | 0.3356 |
| THAP8 | 199745 | -1.121 | 0.3356 |
| SLC44A5 | 204962 | -0.746 | 0.3356 |
| PHACTR1 | 221692 | 0.778 | 0.3356 |
| HIST2H2AB | 317772 | -0.794 | 0.3356 |
| S100A7A | 338324 | 0.906 | 0.3356 |
| SMCO2 | 341346 | -0.975 | 0.3356 |
| POTEE | 445582 | -1.001 | 0.3356 |
| PPIAL4G | 644591 | -0.647 | 0.3356 |
| RGPD3 | 653489 | -0.805 | 0.3356 |
| SFTPA1 | 653509 | -0.660 | 0.3356 |
| ELOA3B | 728929 | -0.845 | 0.3356 |
| SFTPA2 | 729238 | -0.655 | 0.3356 |
| CD300LD | 100131439 | 1.268 | 0.3356 |
| ELOA3D | 100506888 | -0.845 | 0.3356 |
| LOC105373381 | 105373381 | 1.175 | 0.3356 |
| ELOA3C | 107983955 | -0.845 | 0.3356 |
| ATP1B3 | 483 | -0.771 | 0.3356 |
| CD3E | 916 | -0.786 | 0.3356 |
| CDK9 | 1025 | -1.087 | 0.3356 |
| CHIT1 | 1118 | 0.732 | 0.3356 |
| COL1A2 | 1278 | -0.712 | 0.3356 |
| CR2 | 1380 | 0.591 | 0.3356 |
| CTSB | 1508 | -0.770 | 0.3356 |
| PHC2 | 1912 | 0.974 | 0.3356 |
| FLNC | 2318 | 0.470 | 0.3356 |
| FUT8 | 2530 | -0.534 | 0.3356 |
| HBE1 | 3046 | 0.923 | 0.3356 |
| HMGCS2 | 3158 | -0.958 | 0.3356 |
| ILK | 3611 | 0.698 | 0.3356 |
| INCENP | 3619 | -0.718 | 0.3356 |
| INPP5D | 3635 | -0.852 | 0.3356 |
| KRT31 | 3881 | 1.083 | 0.3356 |
| KRT33B | 3884 | 1.079 | 0.3356 |
| LMO7 | 4008 | -0.975 | 0.3356 |
| NCK1 | 4690 | -0.638 | 0.3356 |
| PAFAH1B2 | 5049 | 1.379 | 0.3356 |
| PGD | 5226 | -0.868 | 0.3356 |
| PODXL | 5420 | 0.862 | 0.3356 |
| RAD23A | 5886 | 0.703 | 0.3356 |
| RAD23B | 5887 | 0.721 | 0.3356 |
| RAN | 5901 | -0.928 | 0.3356 |
| RNASE2 | 6036 | 0.923 | 0.3356 |
| RNASEL | 6041 | 1.111 | 0.3356 |
| SDC1 | 6382 | -0.731 | 0.3356 |
| SLC12A4 | 6560 | 0.401 | 0.3356 |
| SPTB | 6710 | 0.648 | 0.3356 |
| MADCAM1 | 8174 | 0.784 | 0.3356 |
| HIST1H2BL | 8340 | -0.750 | 0.3356 |
| HIST1H2BM | 8342 | -0.750 | 0.3356 |
| CDK10 | 8558 | 0.912 | 0.3356 |
| RIPK1 | 8737 | 0.822 | 0.3356 |
| SYNJ2 | 8871 | 0.777 | 0.3356 |
| KRT75 | 9119 | -1.295 | 0.3356 |
| KIF14 | 9928 | -0.941 | 0.3356 |
| ABCB6 | 10058 | -0.589 | 0.3356 |
| TRAP1 | 10131 | 0.654 | 0.3356 |
| CPQ | 10404 | -0.663 | 0.3356 |
| SMR3B | 10879 | -0.969 | 0.3356 |
| YWHAQ | 10971 | 0.874 | 0.3356 |
| PRR4 | 11272 | 1.177 | 0.3356 |
| ENDOD1 | 23052 | -0.540 | 0.3356 |
| GCAT | 23464 | -0.940 | 0.3356 |
| TECPR1 | 25851 | 1.016 | 0.3356 |
| PNISR | 25957 | -0.770 | 0.3356 |
| OLA1 | 29789 | -1.005 | 0.3356 |
| EEF2K | 29904 | -0.951 | 0.3356 |
| XRN1 | 54464 | 0.529 | 0.3356 |
| GNB1L | 54584 | 0.969 | 0.3356 |
| MRM3 | 55178 | 1.182 | 0.3356 |
| SCYL2 | 55681 | -0.717 | 0.3356 |
| CD248 | 57124 | -0.635 | 0.3356 |
| LSM2 | 57819 | -0.809 | 0.3356 |
| NLRC4 | 58484 | 0.913 | 0.3356 |
| EPB41L4A | 64097 | -0.821 | 0.3356 |
| ZSCAN5A | 79149 | -0.957 | 0.3356 |
| CCDC121 | 79635 | -0.729 | 0.3356 |
| ATP8B4 | 79895 | 0.702 | 0.3356 |
| ABHD11 | 83451 | -1.065 | 0.3356 |
| ELMSAN1 | 91748 | 0.760 | 0.3356 |
| HELB | 92797 | 0.998 | 0.3356 |
| EGLN2 | 112398 | -0.942 | 0.3356 |
| CSMD2 | 114784 | 0.843 | 0.3356 |
| OR2T10 | 127069 | -0.944 | 0.3356 |
| HIST3H2BB | 128312 | -0.750 | 0.3356 |
| H1FOO | 132243 | -0.798 | 0.3356 |
| RIPOR3 | 140876 | 0.832 | 0.3356 |
| TET3 | 200424 | -0.814 | 0.3356 |
| C9orf43 | 257169 | -0.845 | 0.3356 |
| ACTBL2 | 345651 | 1.203 | 0.3356 |
| RTN4RL2 | 349667 | -0.744 | 0.3356 |
| OR5K4 | 403278 | -0.849 | 0.3356 |
| PPIAL4D | 645142 | -0.636 | 0.3356 |
| TMEM200C | 645369 | -0.920 | 0.3356 |
| PPIAL4A | 653505 | -0.636 | 0.3356 |
| PPIAL4C | 653598 | -0.636 | 0.3356 |
| PPIAL4F | 728945 | -0.636 | 0.3356 |
| PPIAL4E | 730262 | -0.636 | 0.3356 |
| PPIAL4H | 105371242 | -0.636 | 0.3356 |
| ABCC6 | 368 | -0.436 | 0.3356 |
| NUCB1 | 4924 | 0.802 | 0.3356 |
| PLEK | 5341 | 0.976 | 0.3356 |
| AP4B1 | 10717 | -1.332 | 0.3356 |
| NUP62 | 23636 | 0.985 | 0.3356 |
| NKIRAS1 | 28512 | -1.189 | 0.3356 |
| UBE3B | 89910 | 0.757 | 0.3356 |
| KRT3 | 3850 | -0.492 | 0.3356 |
| NEK4 | 6787 | 0.066 | 0.3356 |
| GBF1 | 8729 | 0.053 | 0.3356 |
| STAU2 | 27067 | 0.035 | 0.3356 |
| PALB2 | 79728 | -0.051 | 0.3356 |
| TTC25 | 83538 | -0.023 | 0.3356 |
| SPTBN2 | 6712 | -0.068 | 0.3356 |
| TREH | 11181 | -0.018 | 0.3356 |
| RNASE4 | 6038 | 1.020 | 0.3359 |
| TPM4 | 7171 | 1.663 | 0.3362 |
| SLC2A2 | 6514 | 2.368 | 0.3366 |
| FCN2 | 2220 | -0.179 | 0.3371 |
| THBS4 | 7060 | -0.923 | 0.3373 |
| RNF123 | 63891 | -2.404 | 0.3377 |
| FCN3 | 8547 | -0.113 | 0.3388 |
| SH3BGRL3 | 83442 | 1.763 | 0.3394 |
| FARP1 | 10160 | -1.186 | 0.3397 |
| CFB | 629 | -0.072 | 0.3426 |
| C4B | 721 | 1.660 | 0.3428 |
| PSMB6 | 5694 | 1.434 | 0.3429 |
| FCGR3A | 2214 | 1.054 | 0.3437 |
| CTSS | 1520 | 1.484 | 0.3443 |
| FRMPD1 | 22844 | 2.081 | 0.3444 |
| PVR | 5817 | 0.264 | 0.3446 |
| CD93 | 22918 | 0.764 | 0.3446 |
| SERPINB1 | 1992 | 0.616 | 0.3470 |
| SIVA1 | 10572 | 2.038 | 0.3491 |
| SGSM2 | 9905 | 1.465 | 0.3493 |
| SERPINA1 | 5265 | 0.076 | 0.3498 |
| C1orf56 | 54964 | 0.928 | 0.3505 |
| MSN | 4478 | -1.205 | 0.3507 |
| NID1 | 4811 | -1.742 | 0.3508 |
| HABP2 | 3026 | -0.101 | 0.3512 |
| MAP9 | 79884 | 1.766 | 0.3518 |
| RALGAPB | 57148 | -1.556 | 0.3535 |
| ASPSCR1 | 79058 | -2.104 | 0.3537 |
| TTC6 | 319089 | -1.437 | 0.3542 |
| SERPINE1 | 5054 | -1.581 | 0.3549 |
| GPLD1 | 2822 | 0.143 | 0.3560 |
| ZNF550 | 162972 | 1.856 | 0.3569 |
| PRKG2 | 5593 | -1.924 | 0.3575 |
| STOM | 2040 | -0.806 | 0.3578 |
| F12 | 2161 | -0.985 | 0.3585 |
| ACTA1 | 58 | 0.292 | 0.3597 |
| ACTC1 | 70 | 0.292 | 0.3597 |
| MMP9 | 4318 | -1.580 | 0.3607 |
| HPSE | 10855 | -1.223 | 0.3616 |
| ADIPOQ | 9370 | 1.329 | 0.3616 |
| ANKRD36 | 375248 | 0.770 | 0.3620 |
| CTSF | 8722 | 1.879 | 0.3632 |
| PKP1 | 5317 | -1.491 | 0.3647 |
| CSF1R | 1436 | -0.733 | 0.3652 |
| COPB1 | 1315 | 1.240 | 0.3663 |
| A1BG | 1 | 0.107 | 0.3670 |
| WWP1 | 11059 | -0.838 | 0.3684 |
| RSPH14 | 27156 | 1.715 | 0.3687 |
| RNF219 | 79596 | -1.822 | 0.3714 |
| BPGM | 669 | 1.420 | 0.3721 |
| CD5L | 922 | -0.971 | 0.3722 |
| KRT10 | 3858 | -0.259 | 0.3725 |
| INHBC | 3626 | -1.614 | 0.3732 |
| SH3D19 | 152503 | 2.548 | 0.3741 |
| LAMP1 | 3916 | -1.698 | 0.3743 |
| F11 | 2160 | 0.973 | 0.3777 |
| HPCAL4 | 51440 | 3.020 | 0.3805 |
| GSTM5 | 2949 | -0.259 | 0.3810 |
| SPECC1L | 23384 | 0.714 | 0.3825 |
| ALDOA | 226 | 1.209 | 0.3827 |
| MB | 4151 | 2.120 | 0.3831 |
| CDK5RAP2 | 55755 | -1.575 | 0.3846 |
| LAMC1 | 3915 | -0.719 | 0.3846 |
| B3GNT8 | 374907 | -0.549 | 0.3851 |
| CHD5 | 26038 | -1.006 | 0.3865 |
| HRG | 3273 | -0.104 | 0.3875 |
| ACE | 1636 | -1.832 | 0.3887 |
| CFHR5 | 81494 | 0.339 | 0.3901 |
| PCOLCE | 5118 | 1.413 | 0.3902 |
| ZNF93 | 81931 | -1.600 | 0.3921 |
| GLIPR2 | 152007 | 1.918 | 0.3924 |
| KRT13 | 3860 | -2.183 | 0.3934 |
| EFEMP1 | 2202 | -0.225 | 0.3953 |
| ADAMTS4 | 9507 | 1.594 | 0.3960 |
| APAF1 | 317 | -0.584 | 0.3963 |
| PATL1 | 219988 | -1.793 | 0.3979 |
| PRRC2C | 23215 | -1.019 | 0.3993 |
| ALB | 213 | 0.253 | 0.4038 |
| SYCE1 | 93426 | -1.470 | 0.4054 |
| PHACTR2 | 9749 | 1.223 | 0.4062 |
| CIT | 11113 | -1.058 | 0.4070 |
| LCP1 | 3936 | -0.275 | 0.4077 |
| C9 | 735 | -0.084 | 0.4107 |
| COLEC10 | 10584 | -2.261 | 0.4107 |
| C1R | 715 | -0.118 | 0.4112 |
| COL18A1 | 80781 | -0.718 | 0.4123 |
| POSTN | 10631 | 1.460 | 0.4131 |
| GALNT17 | 64409 | 0.554 | 0.4151 |
| DBH | 1621 | -1.461 | 0.4157 |
| LGALS7 | 3963 | -1.457 | 0.4160 |
| LGALS7B | 653499 | -1.457 | 0.4160 |
| BCHE | 590 | -0.145 | 0.4171 |
| CFHR2 | 3080 | -2.300 | 0.4177 |
| F13A1 | 2162 | 1.471 | 0.4180 |
| NRIP1 | 8204 | 1.778 | 0.4206 |
| FSTL4 | 23105 | -1.434 | 0.4209 |
| HIST1H4I | 8294 | -0.913 | 0.4210 |
| HIST1H4A | 8359 | -0.913 | 0.4210 |
| HIST1H4D | 8360 | -0.913 | 0.4210 |
| HIST1H4F | 8361 | -0.913 | 0.4210 |
| HIST1H4K | 8362 | -0.913 | 0.4210 |
| HIST1H4J | 8363 | -0.913 | 0.4210 |
| HIST1H4C | 8364 | -0.913 | 0.4210 |
| HIST1H4H | 8365 | -0.913 | 0.4210 |
| HIST1H4B | 8366 | -0.913 | 0.4210 |
| HIST1H4E | 8367 | -0.913 | 0.4210 |
| HIST1H4L | 8368 | -0.913 | 0.4210 |
| HIST2H4A | 8370 | -0.913 | 0.4210 |
| HIST4H4 | 121504 | -0.913 | 0.4210 |
| HIST2H4B | 554313 | -0.913 | 0.4210 |
| DOCK11 | 139818 | 0.775 | 0.4263 |
| PDK1 | 5163 | -1.809 | 0.4267 |
| KRT77 | 374454 | -1.085 | 0.4271 |
| ERAP2 | 64167 | 0.548 | 0.4275 |
| CTCFL | 140690 | -1.457 | 0.4305 |
| ZNF280D | 54816 | 1.899 | 0.4311 |
| HP | 3240 | 0.222 | 0.4372 |
| C4B_2 | 100293534 | -0.080 | 0.4375 |
| APOF | 319 | -0.153 | 0.4379 |
| CRYBG3 | 131544 | -0.871 | 0.4381 |
| DNAH1 | 25981 | -1.067 | 0.4401 |
| EPPK1 | 83481 | 1.019 | 0.4428 |
| POLR1A | 25885 | -0.900 | 0.4429 |
| ITPRID2 | 6744 | -1.371 | 0.4439 |
| HLA-B | 3106 | -1.907 | 0.4464 |
| FABP5 | 2171 | 1.846 | 0.4472 |
| SHPRH | 257218 | 1.500 | 0.4476 |
| RDX | 5962 | -0.862 | 0.4477 |
| ANKRD36C | 400986 | -0.975 | 0.4481 |
| MAFG | 4097 | 2.136 | 0.4524 |
| C7 | 730 | -0.103 | 0.4541 |
| SLC38A10 | 124565 | 1.141 | 0.4546 |
| GP5 | 2814 | 1.085 | 0.4563 |
| ITIH3 | 3699 | 0.079 | 0.4567 |
| CTBS | 1486 | -0.183 | 0.4583 |
| ANKRD29 | 147463 | 0.703 | 0.4595 |
| NRP1 | 8829 | 0.586 | 0.4603 |
| NFKB1 | 4790 | -1.183 | 0.4603 |
| EIF3G | 8666 | -1.686 | 0.4619 |
| C1orf112 | 55732 | 1.760 | 0.4630 |
| PRCP | 5547 | -0.874 | 0.4647 |
| KMO | 8564 | -1.676 | 0.4675 |
| MYH15 | 22989 | -0.899 | 0.4680 |
| TGS1 | 96764 | -1.847 | 0.4680 |
| APOB | 338 | -0.071 | 0.4692 |
| SERPIND1 | 3053 | -0.065 | 0.4706 |
| CCDC7 | 79741 | -0.907 | 0.4709 |
| NES | 10763 | 1.123 | 0.4719 |
| FLT4 | 2324 | -1.104 | 0.4733 |
| PROCR | 10544 | -0.774 | 0.4735 |
| ORM2 | 5005 | -0.135 | 0.4739 |
| PKM | 5315 | -1.350 | 0.4744 |
| VWF | 7450 | 0.227 | 0.4745 |
| LAMB1 | 3912 | -1.499 | 0.4746 |
| KRT16 | 3868 | -0.457 | 0.4747 |
| KRT6A | 3853 | 1.789 | 0.4753 |
| RPS6KA3 | 6197 | 1.815 | 0.4761 |
| CFL1 | 1072 | 1.508 | 0.4765 |
| PRDX6 | 9588 | -1.506 | 0.4783 |
| PLEKHA6 | 22874 | -1.079 | 0.4807 |
| KRT85 | 3891 | -1.016 | 0.4810 |
| DHX30 | 22907 | 1.636 | 0.4812 |
| ANKRD36B | 57730 | -1.244 | 0.4817 |
| PHF10 | 55274 | -1.134 | 0.4822 |
| CTSD | 1509 | 0.887 | 0.4831 |
| KRT81 | 3887 | -0.988 | 0.4845 |
| PON3 | 5446 | 0.101 | 0.4860 |
| CFAP57 | 149465 | -0.655 | 0.4861 |
| KRT83 | 3889 | -0.975 | 0.4861 |
| CNDP1 | 84735 | -0.147 | 0.4867 |
| KRT86 | 3892 | -0.969 | 0.4869 |
| F10 | 2159 | 0.096 | 0.4874 |
| PFN1 | 5216 | 1.176 | 0.4879 |
| NUP98 | 4928 | 1.305 | 0.4881 |
| SLC3A2 | 6520 | 1.097 | 0.4890 |
| APOH | 350 | 0.072 | 0.4899 |
| LARP1B | 55132 | -1.492 | 0.4911 |
| TTN | 7273 | 1.229 | 0.4915 |
| GAPDH | 2597 | 0.737 | 0.4917 |
| TENM3 | 55714 | -0.782 | 0.4918 |
| CFHR4 | 10877 | -1.237 | 0.4933 |
| NAPEPLD | 222236 | -1.214 | 0.4936 |
| GFAP | 2670 | -1.257 | 0.4938 |
| KRT8 | 3856 | 2.622 | 0.4948 |
| COLEC11 | 78989 | -1.215 | 0.4956 |
| LCORL | 254251 | -0.671 | 0.4961 |
| HEG1 | 57493 | 1.049 | 0.5018 |
| IGFBP3 | 3486 | 0.260 | 0.5030 |
| APMAP | 57136 | -0.223 | 0.5044 |
| SPP2 | 6694 | 0.314 | 0.5045 |
| ADA2 | 51816 | 1.111 | 0.5063 |
| APEH | 327 | -0.873 | 0.5064 |
| CTSH | 1512 | -0.903 | 0.5092 |
| RANBP17 | 64901 | -0.921 | 0.5103 |
| NECTIN2 | 5819 | -1.661 | 0.5118 |
| SCEL | 8796 | 0.968 | 0.5125 |
| APOE | 348 | 0.096 | 0.5129 |
| TNC | 3371 | -0.895 | 0.5133 |
| DNAH11 | 8701 | 0.319 | 0.5134 |
| UGP2 | 7360 | 0.840 | 0.5137 |
| C4BPA | 722 | -0.083 | 0.5155 |
| SUMO4 | 387082 | 0.975 | 0.5163 |
| SUV39H1 | 6839 | -0.769 | 0.5171 |
| ANKAR | 150709 | -1.155 | 0.5172 |
| SLC4A1 | 6521 | 1.128 | 0.5184 |
| LTBP1 | 4052 | -0.674 | 0.5185 |
| CDH5 | 1003 | -0.069 | 0.5204 |
| MCOLN3 | 55283 | -1.314 | 0.5207 |
| NUP214 | 8021 | 1.126 | 0.5219 |
| HIST1H2AE | 3012 | -0.791 | 0.5219 |
| HIST1H2AD | 3013 | -0.791 | 0.5219 |
| HIST1H2AI | 8329 | -0.791 | 0.5219 |
| HIST1H2AK | 8330 | -0.791 | 0.5219 |
| HIST1H2AJ | 8331 | -0.791 | 0.5219 |
| HIST1H2AL | 8332 | -0.791 | 0.5219 |
| HIST1H2AC | 8334 | -0.791 | 0.5219 |
| HIST1H2AB | 8335 | -0.791 | 0.5219 |
| HIST1H2AM | 8336 | -0.791 | 0.5219 |
| HIST2H2AA3 | 8337 | -0.791 | 0.5219 |
| HIST2H2AC | 8338 | -0.791 | 0.5219 |
| HIST1H2AG | 8969 | -0.791 | 0.5219 |
| H2AFJ | 55766 | -0.791 | 0.5219 |
| HIST1H2AH | 85235 | -0.791 | 0.5219 |
| HIST3H2A | 92815 | -0.791 | 0.5219 |
| HIST2H2AA4 | 723790 | -0.791 | 0.5219 |
| GGH | 8836 | -0.159 | 0.5221 |
| 43720 | 124404 | 0.977 | 0.5240 |
| EPB41L2 | 2037 | -1.858 | 0.5244 |
| URB1 | 9875 | -1.195 | 0.5266 |
| VNN1 | 8876 | -1.001 | 0.5271 |
| BTD | 686 | -0.098 | 0.5297 |
| CROCC2 | 728763 | 1.935 | 0.5307 |
| RGL4 | 266747 | 1.529 | 0.5328 |
| PLA2G7 | 7941 | -0.658 | 0.5361 |
| CCDC126 | 90693 | 0.918 | 0.5361 |
| HRNR | 388697 | -0.212 | 0.5361 |
| RHOXF1 | 158800 | 1.555 | 0.5385 |
| HYOU1 | 10525 | -1.195 | 0.5389 |
| CTTNBP2 | 83992 | -0.851 | 0.5398 |
| ICAM2 | 3384 | -0.570 | 0.5398 |
| FBXL22 | 283807 | 1.219 | 0.5411 |
| VPS18 | 57617 | 0.872 | 0.5417 |
| ATP6V0A4 | 50617 | -0.461 | 0.5424 |
| TXN | 7295 | -0.945 | 0.5424 |
| ALPI | 248 | 0.992 | 0.5460 |
| COMP | 1311 | -0.195 | 0.5468 |
| PRKDC | 5591 | -0.607 | 0.5479 |
| PAF1 | 54623 | -0.900 | 0.5482 |
| LRG1 | 116844 | 0.048 | 0.5513 |
| YWHAG | 7532 | -1.360 | 0.5524 |
| PDIA3 | 2923 | -1.453 | 0.5526 |
| ADAMTS13 | 11093 | 0.780 | 0.5531 |
| KRT17 | 3872 | -1.497 | 0.5544 |
| TRIM4 | 89122 | 1.417 | 0.5550 |
| KNG1 | 3827 | 0.043 | 0.5572 |
| RGS11 | 8786 | 0.925 | 0.5586 |
| TEPSIN | 146705 | -0.787 | 0.5595 |
| ADAMTSL4 | 54507 | 0.610 | 0.5599 |
| SELL | 6402 | -0.079 | 0.5601 |
| PPFIA2 | 8499 | -1.070 | 0.5606 |
| MCAM | 4162 | 0.459 | 0.5614 |
| RNF133 | 168433 | 0.970 | 0.5615 |
| CDK18 | 5129 | 0.784 | 0.5620 |
| MYO15A | 51168 | 0.726 | 0.5623 |
| GSR | 2936 | -0.780 | 0.5631 |
| ALAD | 210 | 0.831 | 0.5643 |
| SERPINF2 | 5345 | -0.066 | 0.5643 |
| FAH | 2184 | -1.257 | 0.5651 |
| SMCHD1 | 23347 | -0.820 | 0.5653 |
| TMEM201 | 199953 | -1.549 | 0.5666 |
| ERBB4 | 2066 | 1.029 | 0.5668 |
| GSN | 2934 | -0.053 | 0.5686 |
| GOLGA6L7 | 728310 | -1.150 | 0.5697 |
| MBL2 | 4153 | -0.106 | 0.5708 |
| MYH8 | 4626 | -0.657 | 0.5731 |
| FKBP1A | 2280 | 0.897 | 0.5735 |
| TTLL7 | 79739 | 0.936 | 0.5748 |
| DDT | 1652 | -0.833 | 0.5755 |
| MAN1A1 | 4121 | -0.125 | 0.5761 |
| DNAH5 | 1767 | 1.107 | 0.5762 |
| KIAA1549L | 25758 | -0.562 | 0.5764 |
| TPR | 7175 | 1.128 | 0.5768 |
| OAF | 220323 | -0.872 | 0.5770 |
| PHLDB1 | 23187 | 0.741 | 0.5774 |
| HAUS3 | 79441 | -1.624 | 0.5774 |
| PEX1 | 5189 | 0.803 | 0.5779 |
| DNAJB11 | 51726 | -1.587 | 0.5786 |
| RPRD2 | 23248 | -1.339 | 0.5796 |
| APC | 324 | 0.548 | 0.5797 |
| WAC | 51322 | -0.945 | 0.5808 |
| PTPRF | 5792 | -0.718 | 0.5815 |
| NME2 | 4831 | 0.874 | 0.5821 |
| NME1 | 4830 | 0.851 | 0.5821 |
| NME1-NME2 | 654364 | 0.785 | 0.5822 |
| KRT20 | 54474 | -0.906 | 0.5824 |
| TRIM37 | 4591 | -0.850 | 0.5831 |
| DNHD1 | 144132 | -0.733 | 0.5832 |
| RASSF10 | 644943 | -1.106 | 0.5837 |
| PGLYRP2 | 114770 | 0.067 | 0.5849 |
| CHN1 | 1123 | 1.312 | 0.5856 |
| GP1BA | 2811 | 0.136 | 0.5863 |
| YWHAH | 7533 | 0.791 | 0.5870 |
| HPD | 3242 | 0.040 | 0.5875 |
| PLCH1 | 23007 | 0.708 | 0.5892 |
| PTPRS | 5802 | 0.868 | 0.5892 |
| C6 | 729 | -0.047 | 0.5906 |
| TGFBI | 7045 | -0.073 | 0.5916 |
| KRT4 | 3851 | -1.191 | 0.5922 |
| ERBB2 | 2064 | 0.985 | 0.5924 |
| ENG | 2022 | 0.763 | 0.5927 |
| ITPR2 | 3709 | -1.153 | 0.5940 |
| BCORL1 | 63035 | -0.760 | 0.5941 |
| HPR | 3250 | 0.182 | 0.5952 |
| DRICH1 | 51233 | -0.838 | 0.5952 |
| BRWD1 | 54014 | 0.887 | 0.5953 |
| PDE7A | 5150 | 0.822 | 0.5955 |
| SERPINA4 | 5267 | -0.070 | 0.5961 |
| RBBP8 | 5932 | 1.120 | 0.5963 |
| RBP4 | 5950 | -0.057 | 0.5966 |
| SCG3 | 29106 | -1.099 | 0.5972 |
| FASTKD3 | 79072 | 0.988 | 0.5976 |
| PAK1IP1 | 55003 | 0.847 | 0.5985 |
| TAF7L | 54457 | -0.821 | 0.6017 |
| F5 | 2153 | -0.078 | 0.6023 |
| TPM2 | 7169 | 0.512 | 0.6024 |
| IGFBP2 | 3485 | 0.935 | 0.6034 |
| CPN2 | 1370 | -0.038 | 0.6038 |
| MX2 | 4600 | -0.736 | 0.6038 |
| VCAM1 | 7412 | -0.114 | 0.6063 |
| ADAMTSL3 | 57188 | 0.932 | 0.6078 |
| BNIP2 | 663 | 0.572 | 0.6091 |
| NARS | 4677 | -1.034 | 0.6109 |
| UNC5B | 219699 | -0.744 | 0.6121 |
| NIN | 51199 | -0.726 | 0.6136 |
| ITGB1 | 3688 | 0.648 | 0.6144 |
| SMCO1 | 255798 | 0.732 | 0.6156 |
| FER1L6 | 654463 | -0.658 | 0.6166 |
| PRDX2 | 7001 | -0.445 | 0.6168 |
| ASXL3 | 80816 | -0.528 | 0.6191 |
| REV1 | 51455 | -1.317 | 0.6197 |
| GOLM1 | 51280 | -1.135 | 0.6212 |
| TF | 7018 | 0.079 | 0.6214 |
| LILRA3 | 11026 | -0.746 | 0.6220 |
| TRAK2 | 66008 | -0.877 | 0.6240 |
| HSFX4 | 101927685 | 0.686 | 0.6247 |
| HSFX3 | 101928917 | 0.686 | 0.6247 |
| DKK3 | 27122 | 0.720 | 0.6249 |
| ACIN1 | 22985 | 1.396 | 0.6250 |
| LPA | 4018 | -0.634 | 0.6278 |
| PLXNB1 | 5364 | -0.960 | 0.6300 |
| ORM1 | 5004 | -0.127 | 0.6300 |
| SP9 | 100131390 | -1.631 | 0.6306 |
| PROC | 5624 | 0.115 | 0.6310 |
| ARG1 | 383 | -0.695 | 0.6319 |
| CPN1 | 1369 | -0.056 | 0.6322 |
| SP8 | 221833 | -1.656 | 0.6324 |
| FSD1 | 79187 | 1.218 | 0.6334 |
| CGN | 57530 | -0.755 | 0.6344 |
| KRT15 | 3866 | 1.370 | 0.6346 |
| SLMAP | 7871 | 0.889 | 0.6401 |
| C4BPB | 725 | -0.835 | 0.6425 |
| FGB | 2244 | -0.852 | 0.6427 |
| KRT14 | 3861 | -0.241 | 0.6427 |
| AK1 | 203 | 0.763 | 0.6428 |
| PARD3 | 56288 | 0.629 | 0.6439 |
| CFI | 3426 | -0.044 | 0.6454 |
| HBB | 3043 | -0.253 | 0.6470 |
| SPIN1 | 10927 | 0.831 | 0.6481 |
| RBM6 | 10180 | 1.391 | 0.6490 |
| SERPINA7 | 6906 | -0.052 | 0.6508 |
| PLEKHD1 | 400224 | -0.908 | 0.6509 |
| ENO1 | 2023 | -0.793 | 0.6514 |
| SPOCD1 | 90853 | -0.620 | 0.6527 |
| TTR | 7276 | -0.055 | 0.6553 |
| IGFALS | 3483 | -0.095 | 0.6573 |
| MPO | 4353 | 0.422 | 0.6588 |
| CBLN4 | 140689 | -0.726 | 0.6592 |
| BLOC1S6 | 26258 | -0.734 | 0.6629 |
| RAP1B | 5908 | 0.628 | 0.6629 |
| RAP1A | 5906 | 0.612 | 0.6646 |
| C1QB | 713 | 0.089 | 0.6655 |
| ASCC3 | 10973 | -0.582 | 0.6671 |
| SPARC | 6678 | 0.221 | 0.6680 |
| CTSZ | 1522 | 0.632 | 0.6682 |
| ANKRD24 | 170961 | -0.875 | 0.6683 |
| MEGF8 | 1954 | -0.819 | 0.6686 |
| ARHGEF40 | 55701 | 1.084 | 0.6693 |
| THBS3 | 7059 | 1.032 | 0.6695 |
| MUC16 | 94025 | -0.487 | 0.6703 |
| FCGBP | 8857 | -0.547 | 0.6704 |
| ITIH1 | 3697 | -0.042 | 0.6711 |
| ERVW-1 | 30816 | 0.824 | 0.6717 |
| CDC23 | 8697 | 0.819 | 0.6723 |
| SERPINA5 | 5104 | -0.052 | 0.6723 |
| TSR1 | 55720 | -0.647 | 0.6731 |
| DNAH8 | 1769 | -0.298 | 0.6739 |
| CYP27C1 | 339761 | -0.472 | 0.6765 |
| CHMP4A | 29082 | -0.052 | 0.6765 |
| ACO1 | 48 | 0.651 | 0.6785 |
| NTN4 | 59277 | -0.824 | 0.6800 |
| CEACAM20 | 125931 | 1.064 | 0.6805 |
| CIAPIN1 | 57019 | -0.481 | 0.6819 |
| ANKRD28 | 23243 | 0.620 | 0.6822 |
| MMP2 | 4313 | -0.618 | 0.6833 |
| HAUS5 | 23354 | 1.031 | 0.6849 |
| FUCA2 | 2519 | 0.576 | 0.6851 |
| IGF2R | 3482 | -0.478 | 0.6871 |
| KRT6B | 3854 | 0.880 | 0.6878 |
| MYH3 | 4621 | -0.382 | 0.6895 |
| NRG2 | 9542 | 0.923 | 0.6908 |
| B4GAT1 | 11041 | -0.592 | 0.6908 |
| HSP90B1 | 7184 | -0.607 | 0.6918 |
| IL1RAP | 3556 | 0.658 | 0.6925 |
| TPM1 | 7168 | -0.423 | 0.6927 |
| KIF27 | 55582 | -0.634 | 0.6943 |
| SYNDIG1 | 79953 | -0.580 | 0.6944 |
| DCD | 117159 | 0.198 | 0.6951 |
| TRIM9 | 114088 | 0.671 | 0.6956 |
| F9 | 2158 | 0.037 | 0.6965 |
| FETUB | 26998 | -0.080 | 0.6966 |
| FERMT3 | 83706 | -0.615 | 0.6973 |
| PTPRJ | 5795 | -0.584 | 0.6975 |
| ACTB | 60 | 0.112 | 0.6977 |
| ACTG1 | 71 | 0.112 | 0.6977 |
| KRT72 | 140807 | -0.925 | 0.6985 |
| MASP2 | 10747 | -0.051 | 0.6990 |
| APOL1 | 8542 | -0.046 | 0.7000 |
| SBNO2 | 22904 | -0.794 | 0.7021 |
| OPTN | 10133 | 0.505 | 0.7031 |
| H6PD | 9563 | -0.703 | 0.7036 |
| FGG | 2266 | -0.475 | 0.7045 |
| CFHR3 | 10878 | 0.878 | 0.7047 |
| MYH7 | 4625 | -0.382 | 0.7051 |
| MTPN | 136319 | -0.559 | 0.7093 |
| PTGDS | 5730 | -0.070 | 0.7093 |
| CETP | 1071 | 0.319 | 0.7101 |
| HIST1H2BD | 3017 | 0.602 | 0.7104 |
| UBE2V1 | 7335 | -0.475 | 0.7108 |
| PM20D1 | 148811 | 0.505 | 0.7124 |
| NUP153 | 9972 | -0.805 | 0.7125 |
| TFRC | 7037 | 0.576 | 0.7126 |
| PTGFRN | 5738 | 0.855 | 0.7147 |
| TRIP13 | 9319 | 0.567 | 0.7150 |
| MYO1E | 4643 | 0.957 | 0.7152 |
| MYO9B | 4650 | 0.855 | 0.7154 |
| HBA1 | 3039 | -0.214 | 0.7155 |
| HBA2 | 3040 | -0.214 | 0.7155 |
| FLG | 2312 | 0.499 | 0.7160 |
| LAMP2 | 3920 | -0.786 | 0.7171 |
| SSC5D | 284297 | -0.597 | 0.7174 |
| PRSS1 | 5644 | 0.062 | 0.7182 |
| MYO9A | 4649 | -0.487 | 0.7184 |
| APCS | 325 | -0.036 | 0.7193 |
| CCDC38 | 120935 | 0.656 | 0.7218 |
| MYH4 | 4622 | -0.331 | 0.7230 |
| CDH1 | 999 | -0.268 | 0.7240 |
| TGFB1 | 7040 | -0.752 | 0.7257 |
| PPBP | 5473 | -0.075 | 0.7272 |
| FAM3C | 10447 | 0.555 | 0.7275 |
| PNLDC1 | 154197 | -0.521 | 0.7281 |
| TPM3 | 7170 | 0.561 | 0.7298 |
| F7 | 2155 | 0.492 | 0.7302 |
| CDH6 | 1004 | -0.681 | 0.7304 |
| PCDH15 | 65217 | -1.113 | 0.7311 |
| CP | 1356 | -0.029 | 0.7313 |
| TMEM189-UBE2V1 | 387522 | -0.382 | 0.7315 |
| LRRC39 | 127495 | 0.834 | 0.7333 |
| HIST1H2BB | 3018 | 0.532 | 0.7343 |
| HIST1H2BN | 8341 | 0.532 | 0.7343 |
| SAA2-SAA4 | 100528017 | -0.038 | 0.7391 |
| B2M | 567 | 0.089 | 0.7393 |
| C2 | 717 | -0.026 | 0.7396 |
| MRC1 | 4360 | 0.320 | 0.7397 |
| HSPA5 | 3309 | -0.077 | 0.7407 |
| KRT9 | 3857 | -0.078 | 0.7409 |
| KIF19 | 124602 | -0.859 | 0.7427 |
| HIST2H2BF | 440689 | 0.496 | 0.7449 |
| HIST1H2BH | 8345 | 0.496 | 0.7465 |
| HIST1H2BO | 8348 | 0.496 | 0.7465 |
| C7orf25 | 79020 | 0.810 | 0.7471 |
| MET | 4233 | 0.487 | 0.7507 |
| DSG1 | 1828 | -0.743 | 0.7514 |
| CSPG4 | 1464 | 0.286 | 0.7527 |
| DNAH9 | 1770 | 0.633 | 0.7533 |
| HSPA2 | 3306 | -0.488 | 0.7538 |
| KRT73 | 319101 | -0.895 | 0.7549 |
| SPINK5 | 11005 | -0.486 | 0.7555 |
| MINPP1 | 9562 | -0.286 | 0.7555 |
| NCAPH | 23397 | 0.549 | 0.7556 |
| ANPEP | 290 | 0.089 | 0.7557 |
| MACF1 | 23499 | -0.546 | 0.7581 |
| SMARCC1 | 6599 | -0.338 | 0.7585 |
| ROBO4 | 54538 | -0.626 | 0.7585 |
| VPS4A | 27183 | -0.686 | 0.7603 |
| HLA-A | 3105 | 0.636 | 0.7608 |
| SERPINA6 | 866 | -0.030 | 0.7610 |
| CCDC88A | 55704 | -0.520 | 0.7622 |
| CLEC3B | 7123 | -0.028 | 0.7642 |
| IQUB | 154865 | 0.793 | 0.7642 |
| DSP | 1832 | -0.446 | 0.7657 |
| HBG1 | 3047 | -0.704 | 0.7674 |
| DMKN | 93099 | -0.613 | 0.7682 |
| C4orf54 | 285556 | -0.501 | 0.7683 |
| BARD1 | 580 | 0.836 | 0.7687 |
| GOLGA8M | 653720 | -0.474 | 0.7706 |
| SPDYA | 245711 | -0.598 | 0.7707 |
| S100A6 | 6277 | 0.677 | 0.7727 |
| SBSN | 374897 | 0.131 | 0.7728 |
| AOC3 | 8639 | -0.497 | 0.7763 |
| AFM | 173 | 0.053 | 0.7794 |
| KMT2D | 8085 | -0.231 | 0.7796 |
| OGN | 4969 | -0.516 | 0.7807 |
| JUP | 3728 | -0.457 | 0.7849 |
| CCDC144A | 9720 | -0.332 | 0.7856 |
| CRY1 | 1407 | -0.650 | 0.7871 |
| CUL4B | 8450 | 0.379 | 0.7896 |
| XRN2 | 22803 | -0.300 | 0.7904 |
| APP | 351 | 0.428 | 0.7912 |
| KRT80 | 144501 | 0.615 | 0.7914 |
| KRT74 | 121391 | -0.724 | 0.7947 |
| TFR2 | 7036 | -0.380 | 0.7950 |
| ATP6V0A1 | 535 | -0.287 | 0.7972 |
| CLU | 1191 | 0.025 | 0.7999 |
| TOM1L2 | 146691 | -0.625 | 0.8015 |
| CD99 | 4267 | 0.618 | 0.8033 |
| KRT32 | 3882 | 0.754 | 0.8042 |
| ATRN | 8455 | 0.023 | 0.8045 |
| KLHL42 | 57542 | 0.540 | 0.8054 |
| LUM | 4060 | -0.019 | 0.8072 |
| CNGB1 | 1258 | -0.372 | 0.8079 |
| PLXDC2 | 84898 | 0.367 | 0.8111 |
| TUT4 | 23318 | -0.260 | 0.8111 |
| UBC | 7316 | -0.242 | 0.8127 |
| GNPTG | 84572 | 0.570 | 0.8142 |
| PON1 | 5444 | -0.032 | 0.8154 |
| L3MBTL4 | 91133 | -0.438 | 0.8157 |
| NCAM1 | 4684 | -0.073 | 0.8167 |
| MON2 | 23041 | 0.268 | 0.8171 |
| ATXN2L | 11273 | 0.393 | 0.8172 |
| AMBP | 259 | 0.030 | 0.8188 |
| MYH9 | 4627 | -0.143 | 0.8192 |
| AK7 | 122481 | 0.647 | 0.8193 |
| CD14 | 929 | 0.035 | 0.8200 |
| IGFBP4 | 3487 | 0.580 | 0.8211 |
| NRP2 | 8828 | 0.269 | 0.8212 |
| PCSK1N | 27344 | -0.263 | 0.8231 |
| FLG2 | 388698 | 0.350 | 0.8235 |
| FAM186B | 84070 | -0.455 | 0.8255 |
| LILRB1 | 10859 | 0.328 | 0.8275 |
| ITPR3 | 3710 | -0.318 | 0.8279 |
| NOTCH1 | 4851 | 0.280 | 0.8281 |
| LILRA1 | 11024 | 0.376 | 0.8283 |
| EIF2AK4 | 440275 | -0.485 | 0.8284 |
| AHSG | 197 | -0.020 | 0.8298 |
| AFDN | 4301 | -0.332 | 0.8308 |
| KLKB1 | 3818 | -0.024 | 0.8338 |
| TAF1L | 138474 | -0.160 | 0.8340 |
| PLG | 5340 | 0.021 | 0.8351 |
| ODF2L | 57489 | -0.272 | 0.8376 |
| SPTBN5 | 51332 | -0.382 | 0.8378 |
| KTN1 | 3895 | 0.343 | 0.8391 |
| UBB | 7314 | -0.242 | 0.8392 |
| PIP4K2C | 79837 | -0.530 | 0.8400 |
| WFDC3 | 140686 | 0.487 | 0.8408 |
| FAM20C | 56975 | -0.278 | 0.8429 |
| KRT1 | 3848 | -0.048 | 0.8441 |
| CFP | 5199 | -0.371 | 0.8448 |
| ERICH1 | 157697 | 0.392 | 0.8451 |
| MARCO | 8685 | 0.268 | 0.8461 |
| CALML5 | 51806 | -0.506 | 0.8461 |
| HIST1H1C | 3006 | 0.225 | 0.8461 |
| PRG4 | 10216 | 0.050 | 0.8466 |
| F2 | 2147 | 0.028 | 0.8479 |
| HSPA8 | 3312 | 0.350 | 0.8480 |
| HPX | 3263 | 0.016 | 0.8489 |
| CLIC3 | 9022 | 0.339 | 0.8501 |
| ITIH2 | 3698 | 0.014 | 0.8510 |
| UBA52 | 7311 | -0.242 | 0.8520 |
| RPS27A | 6233 | -0.242 | 0.8546 |
| IL6ST | 3572 | 0.261 | 0.8546 |
| TCHP | 84260 | 0.265 | 0.8549 |
| CHGA | 1113 | 0.207 | 0.8556 |
| SUPT6H | 6830 | 0.215 | 0.8557 |
| S100A9 | 6280 | 0.049 | 0.8561 |
| FANCC | 2176 | -0.393 | 0.8571 |
| MMRN2 | 79812 | 0.236 | 0.8575 |
| CLCN6 | 1185 | -0.349 | 0.8577 |
| TSC1 | 7248 | 0.384 | 0.8585 |
| PTPRG | 5793 | -0.338 | 0.8592 |
| H2AFX | 3014 | -0.178 | 0.8602 |
| CFD | 1675 | 0.026 | 0.8604 |
| TRIM71 | 131405 | -0.318 | 0.8612 |
| NUP188 | 23511 | -0.342 | 0.8615 |
| JCHAIN | 3512 | -0.378 | 0.8617 |
| FLNA | 2316 | 0.243 | 0.8619 |
| ISLR | 3671 | 0.387 | 0.8627 |
| HIST1H2AA | 221613 | -0.178 | 0.8633 |
| ANXA6 | 309 | 0.328 | 0.8641 |
| CADPS2 | 93664 | -0.201 | 0.8650 |
| H2AFZ | 3015 | -0.178 | 0.8669 |
| KANSL1 | 284058 | -0.322 | 0.8671 |
| IGF1 | 3479 | -0.237 | 0.8680 |
| CA3 | 761 | -0.216 | 0.8696 |
| LRP1 | 4035 | -0.178 | 0.8702 |
| CES1 | 1066 | 0.205 | 0.8705 |
| AFF4 | 27125 | 0.243 | 0.8709 |
| H2AFV | 94239 | -0.178 | 0.8710 |
| CCDC151 | 115948 | 0.383 | 0.8715 |
| DIAPH1 | 1729 | -0.265 | 0.8715 |
| PYGM | 5837 | 0.213 | 0.8720 |
| GFRA1 | 2674 | -0.554 | 0.8728 |
| DPEP2 | 64174 | -0.383 | 0.8732 |
| PIP | 5304 | 0.207 | 0.8743 |
| KRT5 | 3852 | -0.062 | 0.8771 |
| APOA1 | 335 | -0.013 | 0.8797 |
| ENPP2 | 5168 | -0.175 | 0.8815 |
| HGFAC | 3083 | 0.023 | 0.8818 |
| PGK1 | 5230 | 0.177 | 0.8820 |
| P4HB | 5034 | -0.283 | 0.8828 |
| CNTN1 | 1272 | 0.297 | 0.8832 |
| ADO | 84890 | -0.458 | 0.8855 |
| ADGRF3 | 165082 | 0.145 | 0.8861 |
| DST | 667 | 0.170 | 0.8862 |
| CPB2 | 1361 | -0.018 | 0.8867 |
| APOM | 55937 | 0.014 | 0.8867 |
| LCAT | 3931 | -0.020 | 0.8877 |
| TRHDE | 29953 | 0.137 | 0.8879 |
| F8 | 2157 | 0.176 | 0.8882 |
| ANKRD30B | 374860 | 0.216 | 0.8882 |
| MAEA | 10296 | 0.260 | 0.8896 |
| A2M | 2 | -0.033 | 0.8896 |
| FGA | 2243 | -0.061 | 0.8901 |
| CORO1A | 11151 | 0.193 | 0.8902 |
| C1QC | 714 | -0.012 | 0.8910 |
| ND5 | 4540 | 0.212 | 0.8929 |
| MYO5A | 4644 | -0.118 | 0.8930 |
| EZR | 7430 | 0.286 | 0.8931 |
| TAGLN2 | 8407 | 0.279 | 0.8968 |
| SPATS2L | 26010 | -0.257 | 0.8970 |
| NT5M | 56953 | 0.158 | 0.8981 |
| PAPLN | 89932 | -0.097 | 0.9013 |
| C4A | 720 | -0.002 | 0.9018 |
| TIMP1 | 7076 | 0.296 | 0.9019 |
| VCP | 7415 | -0.208 | 0.9026 |
| PCSK5 | 5125 | -0.125 | 0.9029 |
| N6AMT1 | 29104 | 0.164 | 0.9042 |
| AGTPBP1 | 23287 | -0.132 | 0.9054 |
| HAPLN4 | 404037 | 0.136 | 0.9062 |
| FLOT1 | 10211 | -0.122 | 0.9072 |
| PKP4 | 8502 | 0.116 | 0.9075 |
| APOC3 | 345 | -0.031 | 0.9087 |
| MYH1 | 4619 | -0.091 | 0.9087 |
| KRT2 | 3849 | 0.042 | 0.9101 |
| MTM1 | 4534 | -0.198 | 0.9105 |
| COL6A3 | 1293 | 0.115 | 0.9118 |
| ANXA2 | 302 | 0.127 | 0.9125 |
| MAATS1 | 89876 | -0.105 | 0.9126 |
| FTL | 2512 | -0.251 | 0.9127 |
| LGALS3BP | 3959 | 0.031 | 0.9133 |
| AGT | 183 | -0.013 | 0.9136 |
| NEIL2 | 252969 | 0.121 | 0.9143 |
| KRT71 | 112802 | 0.279 | 0.9144 |
| CEP164 | 22897 | -0.180 | 0.9147 |
| RBM10 | 8241 | -0.158 | 0.9151 |
| PEBP1 | 5037 | 0.135 | 0.9159 |
| EVPL | 2125 | 0.205 | 0.9174 |
| FUCA1 | 2517 | 0.223 | 0.9179 |
| AXL | 558 | -0.160 | 0.9184 |
| ADGRG6 | 57211 | 0.102 | 0.9189 |
| CARD9 | 64170 | -0.117 | 0.9192 |
| BST1 | 683 | 0.205 | 0.9204 |
| TLN1 | 7094 | 0.130 | 0.9206 |
| THBS1 | 7057 | -0.050 | 0.9211 |
| GOLGA6L1 | 283767 | -0.171 | 0.9227 |
| LOC102723623 | 102723623 | -0.171 | 0.9227 |
| PKD1 | 5310 | -0.081 | 0.9228 |
| HSPB1 | 3315 | -0.135 | 0.9238 |
| COG5 | 10466 | 0.193 | 0.9243 |
| APOC1 | 341 | 0.016 | 0.9249 |
| LOC110384692 | 110384692 | -0.008 | 0.9261 |
| RGS6 | 9628 | 0.098 | 0.9274 |
| CST6 | 1474 | 0.164 | 0.9275 |
| OSBPL2 | 9885 | 0.090 | 0.9288 |
| SH2B3 | 10019 | 0.145 | 0.9296 |
| CTDSPL2 | 51496 | -0.172 | 0.9303 |
| TCEA1 | 6917 | 0.113 | 0.9327 |
| TDRD7 | 23424 | 0.102 | 0.9337 |
| PPARD | 5467 | -0.109 | 0.9345 |
| FAM160B1 | 57700 | -0.223 | 0.9347 |
| CEP83 | 51134 | -0.090 | 0.9362 |
| EXOC5 | 10640 | 0.201 | 0.9365 |
| CCDC68 | 80323 | -0.202 | 0.9373 |
| C1RL | 51279 | 0.015 | 0.9382 |
| ABI3BP | 25890 | -0.085 | 0.9383 |
| IGFBP7 | 3490 | 0.094 | 0.9385 |
| CKM | 1158 | -0.172 | 0.9385 |
| S100A4 | 6275 | 0.109 | 0.9394 |
| TULP2 | 7288 | 0.112 | 0.9396 |
| HBG2 | 3048 | 0.206 | 0.9400 |
| PDE6B | 5158 | -0.098 | 0.9400 |
| IGLL5 | 100423062 | -0.019 | 0.9404 |
| LRFN2 | 57497 | 0.139 | 0.9406 |
| TRIB3 | 57761 | 0.140 | 0.9409 |
| PRDX1 | 5052 | -0.131 | 0.9411 |
| DSC2 | 1824 | 0.086 | 0.9434 |
| PCYOX1 | 51449 | 0.019 | 0.9441 |
| LILRA2 | 11027 | -0.124 | 0.9445 |
| NXPE2 | 120406 | 0.096 | 0.9451 |
| HBD | 3045 | 0.059 | 0.9458 |
| VASN | 114990 | 0.056 | 0.9463 |
| PARK7 | 11315 | -0.082 | 0.9467 |
| AZGP1 | 563 | -0.007 | 0.9471 |
| LIMA1 | 51474 | -0.088 | 0.9478 |
| FRA10AC1 | 118924 | -0.080 | 0.9481 |
| BLMH | 642 | -0.191 | 0.9481 |
| TMSB4X | 7114 | 0.047 | 0.9482 |
| CASR | 846 | 0.113 | 0.9488 |
| PZP | 5858 | 0.054 | 0.9511 |
| TEX15 | 56154 | 0.066 | 0.9518 |
| OMD | 4958 | -0.051 | 0.9518 |
| ZNF292 | 23036 | 0.076 | 0.9522 |
| SLC9A3R1 | 9368 | 0.097 | 0.9547 |
| RIOK3 | 8780 | 0.086 | 0.9572 |
| UTS2 | 10911 | -0.077 | 0.9591 |
| SERPINA10 | 51156 | -0.007 | 0.9599 |
| KPRP | 448834 | 0.094 | 0.9600 |
| KRT6C | 286887 | -0.169 | 0.9603 |
| GCN1 | 10985 | -0.082 | 0.9604 |
| NUP205 | 23165 | -0.088 | 0.9620 |
| CNKSR2 | 22866 | 0.055 | 0.9632 |
| CTNND2 | 1501 | 0.044 | 0.9632 |
| ACTN2 | 88 | 0.061 | 0.9633 |
| SMC4 | 10051 | 0.048 | 0.9646 |
| TNFSF18 | 8995 | 0.073 | 0.9650 |
| HSPG2 | 3339 | 0.019 | 0.9660 |
| KIAA2026 | 158358 | -0.109 | 0.9661 |
| LCN2 | 3934 | -0.096 | 0.9664 |
| HIST1H2BG | 8339 | -0.066 | 0.9667 |
| HIST1H2BF | 8343 | -0.066 | 0.9667 |
| HIST1H2BE | 8344 | -0.066 | 0.9667 |
| HIST1H2BI | 8346 | -0.066 | 0.9667 |
| HIST1H2BC | 8347 | -0.066 | 0.9667 |
| HIST2H2BE | 8349 | -0.066 | 0.9667 |
| ATP9A | 10079 | -0.033 | 0.9668 |
| LRBA | 987 | 0.073 | 0.9669 |
| LZIC | 84328 | -0.050 | 0.9679 |
| MEA1 | 4201 | 0.058 | 0.9695 |
| ITPRIP | 85450 | 0.028 | 0.9701 |
| IGLL1 | 3543 | -0.046 | 0.9714 |
| MANSC4 | 100287284 | 0.087 | 0.9737 |
| FASTK | 10922 | -0.057 | 0.9738 |
| SETD4 | 54093 | -0.058 | 0.9740 |
| MYH11 | 4629 | 0.040 | 0.9742 |
| LMAN2 | 10960 | 0.065 | 0.9746 |
| CALML3 | 810 | 0.036 | 0.9767 |
| ARHGAP42 | 143872 | -0.042 | 0.9770 |
| SNCA | 6622 | 0.037 | 0.9789 |
| SH3RF1 | 57630 | -0.022 | 0.9801 |
| LRIG3 | 121227 | -0.039 | 0.9826 |
| ADAMDEC1 | 27299 | -0.022 | 0.9832 |
| LSAMP | 4045 | 0.026 | 0.9839 |
| PROS1 | 5627 | 0.001 | 0.9850 |
| YWHAZ | 7534 | 0.042 | 0.9859 |
| IGFBP1 | 3484 | -0.022 | 0.9860 |
| LRRC36 | 55282 | 0.020 | 0.9864 |
| ACTN1 | 87 | -0.014 | 0.9868 |
| HIST1H1E | 3008 | 0.021 | 0.9873 |
| IL32 | 9235 | 0.022 | 0.9890 |
| RIMS4 | 140730 | -0.017 | 0.9896 |
| SOD3 | 6649 | 0.023 | 0.9902 |
| UBE2Q2 | 92912 | -0.023 | 0.9904 |
| FGD4 | 121512 | -0.011 | 0.9911 |
| CCDC30 | 728621 | -0.017 | 0.9912 |
| GSS | 2937 | 0.010 | 0.9922 |
| FCGR3B | 2215 | 0.014 | 0.9928 |
| APOA5 | 116519 | -0.010 | 0.9929 |
| SERPINC1 | 462 | 0.001 | 0.9935 |
| LRCH1 | 23143 | 0.007 | 0.9954 |
| SEMA4B | 10509 | 0.005 | 0.9961 |
| C3 | 718 | 0.000 | 0.9969 |
| B4GALT5 | 9334 | 0.004 | 0.9975 |
| UACA | 55075 | 0.003 | 0.9976 |
| B4GALT1 | 2683 | 0.004 | 0.9976 |
| CRYGS | 1427 | -0.006 | 0.9978 |
| NME9 | 347736 | 0.003 | 0.9982 |
| RSF1 | 51773 | 0.003 | 0.9984 |
| MXD4 | 10608 | 0.003 | 0.9988 |
