# Supplementary material for: Intermittent fasting from dawn to sunset for four consecutive weeks induces anticancer serum proteome response and improves metabolic syndrome
Source: Sci Rep. 2020 Oct 27;10:18341. doi: 10.1038/s41598-020-73767-w (PMC7592042; doi:10.1038/s41598-020-73767-w)
Supplement: Supplementary file 4 — Supplementary Table 3. [file 41598_2020_73767_MOESM4_ESM.docx]

**Intermittent Fasting from Dawn to Sunset for Four Consecutive Weeks Induces Anticancer Serum Proteome Response and Improves Metabolic Syndrome**

Ayse L. Mindikoglu, M.D., M.P.H.^1, 2^; Mustafa M. Abdulsada, M.B.Ch.B.^1^; Antrix Jain, M.S.^3^; Prasun K. Jalal, M.D.^1, 2^; Sridevi Devaraj, Ph.D.^4^; Zoe R. Wilhelm, B.S.^1^, Antone R. Opekun, M.S., P.A.-C ^1, 5^, Sung Yun Jung, Ph.D.^3, 6^

**Institutions:**

1. Margaret M. and Albert B. Alkek Department of Medicine, Section of Gastroenterology and Hepatology, Baylor College of Medicine, Houston, TX
2. Michael E. DeBakey Department of Surgery, Division of Abdominal Transplantation, Baylor College of Medicine, Houston, TX
3. Advanced Technology Core, Mass Spectrometry Proteomics Core, Baylor College of Medicine, Houston, TX
4. Clinical Chemistry and Point of Care Technology, Texas Children’s Hospital and Health Centers, Department of Pathology and Immunology, Baylor College of Medicine, Houston, TX
5. Department of Pediatrics, Section of Gastroenterology, Nutrition and Hepatology, Baylor College of Medicine, Houston, TX
6. Department of Molecular & Cellular Biology, Baylor College of Medicine, Houston, TX

| **Table S3. The Levels of the Gene Protein Products (GP)s that Are Up- or Downregulated One Week after 4-Week Intermittent Fasting (V3) Compared with the Levels at the End of 4th Week During 4-Week Intermittent Fasting (V2)** | | | |
| --- | --- | --- | --- |
| **Gene Symbol** | **Gene ID** | **Average Paired Log2 Fold Change (V3/V2)** | **Paired P Value** |
| AP5Z1 | 9907 | -5.971 | 0.073 |
| CALR | 811 | 5.515 | 0.010 |
| CALU | 813 | 0.892 | 0.540 |
| CAMP | 820 | 1.277 | 0.559 |
| CD109 | 135228 | 2.900 | 0.028 |
| CROCC | 9696 | -1.170 | 0.467 |
| HIST1H2BA | 255626 | 2.565 | 0.020 |
| HIST1H2BB | 3018 | 2.124 | 0.163 |
| HIST1H2BD | 3017 | 2.019 | 0.193 |
| IGFBP4 | 3487 | 3.148 | 0.096 |
| IGFBP5 | 3488 | -1.704 | 0.183 |
| INTS6 | 26512 | -2.529 | 0.247 |
| KIT | 3815 | -4.040 | 0.011 |
| NIFK | 84365 | 5.334 | 0.019 |
| PIGR | 5284 | -3.432 | 0.007 |
| PLAC1 | 10761 | -3.439 | 0.082 |
| POLK | 51426 | 2.156 | 0.302 |
| POLRMT | 5442 | -3.355 | 0.020 |
| PRKCSH | 5589 | 6.834 | 0.012 |
| SEMA4B | 10509 | 3.381 | 0.019 |
| SRGN | 5552 | 1.725 | 0.619 |
| VPS8 | 23355 | -4.106 | 0.107 |
| FUCA2 | 2519 | -3.426 | 0.024 |
| TBC1D30 | 23329 | -3.601 | 0.037 |
| STIM1 | 6786 | -3.609 | 0.041 |
| ENPP2 | 5168 | -3.906 | 0.015 |
| TRAM1 | 23471 | -4.097 | 0.043 |
| LRRC3 | 81543 | -5.202 | 0.007 |
| COL4A3BP | 10087 | 5.934 | 0.023 |
| FAM217A | 222826 | 5.411 | 0.012 |
| CASP4 | 837 | 5.068 | 0.011 |
| BCL10 | 8915 | 4.716 | 0.009 |
| MEA1 | 4201 | 4.146 | 0.040 |
| SPECC1L | 23384 | 3.969 | 0.022 |
| SERPINE1 | 5054 | 3.936 | 0.037 |
| TGFB1 | 7040 | 3.933 | 0.021 |
| LDHA | 3939 | 3.721 | 0.026 |
| HYOU1 | 10525 | 3.627 | 0.037 |
| CCDC151 | 115948 | 3.624 | 0.045 |
| COL6A1 | 1291 | 3.486 | 0.021 |
| HIST1H2BL | 8340 | 3.406 | 0.010 |
| HIST1H2BM | 8342 | 3.406 | 0.010 |
| HIST1H2BJ | 8970 | 3.406 | 0.010 |
| HIST1H2BK | 85236 | 3.406 | 0.010 |
| MYH7 | 4625 | 3.385 | 0.017 |
| PKLR | 5313 | 3.358 | 0.019 |
| ENO1 | 2023 | 2.971 | 0.050 |
| TTLL6 | 284076 | 2.967 | 0.042 |
| AOC2 | 314 | 2.961 | 0.019 |
| PSMA7 | 5688 | 2.848 | 0.040 |
| HIST3H2BB | 128312 | 2.634 | 0.020 |
| LDLR | 3949 | 2.361 | 0.040 |
| H2BFS | 54145 | 3.406 | 0.010 |
| LOC102724334 | 102724334 | 3.406 | 0.010 |
| A1BG | 1 | 0.071 | 0.280 |
| CTNND2 | 1501 | 0.000 | 1.000 |
| POLR1A | 25885 | -0.001 | 0.999 |
| LCN2 | 3934 | 0.002 | 0.999 |
| LIMA1 | 51474 | -0.004 | 0.998 |
| ARHGEF40 | 55701 | -0.007 | 0.998 |
| APP | 351 | 0.004 | 0.997 |
| CFHR1 | 3078 | 0.015 | 0.997 |
| RNF219 | 79596 | -0.008 | 0.996 |
| WAC | 51322 | -0.008 | 0.996 |
| FSD1 | 79187 | 0.016 | 0.995 |
| SAA4 | 6291 | -0.020 | 0.993 |
| KIAA2026 | 158358 | 0.015 | 0.992 |
| KRT1 | 3848 | -0.003 | 0.992 |
| NAA11 | 84779 | -0.013 | 0.992 |
| SYNDIG1 | 79953 | 0.015 | 0.991 |
| C4orf54 | 285556 | 0.019 | 0.991 |
| SMARCA4 | 6597 | 0.015 | 0.990 |
| RIPK1 | 8737 | 0.016 | 0.990 |
| CCDC126 | 90693 | -0.024 | 0.989 |
| ZFYVE16 | 9765 | 0.020 | 0.989 |
| SP8 | 221833 | 0.042 | 0.987 |
| MANSC4 | 100287284 | -0.038 | 0.987 |
| SP9 | 100131390 | 0.042 | 0.987 |
| PHC2 | 1912 | 0.031 | 0.983 |
| FERMT3 | 83706 | 0.030 | 0.983 |
| B3GNT8 | 374907 | -0.023 | 0.983 |
| LCORL | 254251 | -0.023 | 0.982 |
| CPB2 | 1361 | -0.002 | 0.980 |
| UBE3B | 89910 | -0.030 | 0.979 |
| SNCA | 6622 | 0.040 | 0.978 |
| ACSM5 | 54988 | -0.036 | 0.978 |
| FCGR3A | 2214 | 0.041 | 0.978 |
| PHLDB1 | 23187 | -0.046 | 0.976 |
| KRT33B | 3884 | 0.052 | 0.975 |
| C8G | 733 | 0.003 | 0.975 |
| FYCO1 | 79443 | -0.038 | 0.975 |
| CFHR4 | 10877 | -0.061 | 0.975 |
| XRN2 | 22803 | -0.028 | 0.974 |
| NAGLU | 4669 | 0.039 | 0.974 |
| HSPB1 | 3315 | 0.044 | 0.974 |
| EIF3G | 8666 | -0.078 | 0.973 |
| ND5 | 4540 | -0.058 | 0.973 |
| TMSB10 | 9168 | -0.048 | 0.973 |
| RGS11 | 8786 | 0.047 | 0.972 |
| F13A1 | 2162 | 0.066 | 0.972 |
| RPS6KA3 | 6197 | 0.075 | 0.972 |
| IGFBP7 | 3490 | -0.063 | 0.971 |
| XRN1 | 54464 | -0.028 | 0.971 |
| CTSF | 8722 | 0.056 | 0.971 |
| MERTK | 10461 | 0.068 | 0.970 |
| DNAJB11 | 51726 | 0.118 | 0.970 |
| ARFIP1 | 27236 | 0.113 | 0.969 |
| ZNF697 | 90874 | 0.043 | 0.968 |
| LYSMD3 | 116068 | 0.103 | 0.967 |
| UNC5B | 219699 | -0.052 | 0.966 |
| LOC102724971 | 102724971 | 0.045 | 0.966 |
| HAPLN4 | 404037 | 0.053 | 0.965 |
| PYGM | 5837 | -0.064 | 0.964 |
| LOC107983983 | 107983983 | 0.045 | 0.964 |
| KRT6B | 3854 | -0.081 | 0.963 |
| ADAMTSL4 | 54507 | -0.072 | 0.963 |
| FMN2 | 56776 | 0.127 | 0.962 |
| TCEA1 | 6917 | -0.065 | 0.962 |
| KRT71 | 112802 | -0.127 | 0.962 |
| KRT73 | 319101 | -0.127 | 0.962 |
| KRT74 | 121391 | -0.127 | 0.962 |
| TIAM2 | 26230 | 0.080 | 0.961 |
| KRT9 | 3857 | -0.014 | 0.960 |
| H2AFV | 94239 | -0.046 | 0.960 |
| TTC25 | 83538 | 0.063 | 0.959 |
| H2AFZ | 3015 | -0.046 | 0.959 |
| NRP2 | 8828 | 0.046 | 0.958 |
| GSTO1 | 9446 | -0.089 | 0.957 |
| HIST1H2AA | 221613 | -0.046 | 0.957 |
| PIWIL3 | 440822 | -0.066 | 0.957 |
| LOC105377021 | 105377021 | -0.065 | 0.957 |
| H2AFX | 3014 | -0.046 | 0.956 |
| HSPH1 | 10808 | 0.074 | 0.955 |
| ADGRF3 | 165082 | -0.061 | 0.954 |
| CEP68 | 23177 | 0.061 | 0.954 |
| SPTBN5 | 51332 | -0.137 | 0.954 |
| PITPNM2 | 57605 | -0.057 | 0.954 |
| C4BPB | 725 | -0.058 | 0.954 |
| CCDC7 | 79741 | 0.053 | 0.953 |
| N6AMT1 | 29104 | 0.093 | 0.952 |
| C1orf112 | 55732 | -0.104 | 0.952 |
| TGS1 | 96764 | -0.151 | 0.950 |
| LAMP2 | 3920 | 0.136 | 0.948 |
| LMAN2 | 10960 | -0.110 | 0.947 |
| KRT13 | 3860 | 0.151 | 0.946 |
| ACTN1 | 87 | -0.051 | 0.946 |
| CSAD | 51380 | 0.091 | 0.946 |
| SERPINA6 | 866 | 0.006 | 0.944 |
| ITPRID2 | 6744 | 0.130 | 0.944 |
| PAF1 | 54623 | 0.090 | 0.942 |
| PIP | 5304 | 0.113 | 0.942 |
| TNS4 | 84951 | -0.112 | 0.942 |
| DNAH5 | 1767 | 0.119 | 0.941 |
| ZP4 | 57829 | -0.078 | 0.941 |
| CALML3 | 810 | -0.090 | 0.940 |
| MYO5A | 4644 | 0.065 | 0.938 |
| S100A7 | 6278 | 0.112 | 0.938 |
| NIPSNAP1 | 8508 | 0.152 | 0.937 |
| ANKRD36B | 57730 | -0.121 | 0.937 |
| CORO1A | 11151 | -0.129 | 0.936 |
| AGT | 183 | 0.010 | 0.935 |
| KHNYN | 23351 | 0.093 | 0.934 |
| HSPA8 | 3312 | -0.163 | 0.933 |
| PNLDC1 | 154197 | 0.096 | 0.933 |
| LGALS3BP | 3959 | 0.006 | 0.932 |
| PDLIM5 | 10611 | 0.127 | 0.932 |
| CST3 | 1471 | -0.017 | 0.931 |
| TECPR1 | 25851 | 0.140 | 0.931 |
| CTDSPL2 | 51496 | 0.213 | 0.931 |
| FUCA1 | 2517 | -0.112 | 0.931 |
| AHSG | 197 | -0.009 | 0.930 |
| NXPE2 | 120406 | -0.121 | 0.930 |
| SSC5D | 284297 | 0.124 | 0.928 |
| NCAM1 | 4684 | -0.025 | 0.926 |
| TRIB3 | 57761 | -0.256 | 0.923 |
| CCDC144A | 9720 | -0.112 | 0.922 |
| ROBO4 | 54538 | 0.201 | 0.921 |
| TMSB4X | 7114 | 0.063 | 0.919 |
| PCSK5 | 5125 | 0.104 | 0.917 |
| TRIP13 | 9319 | 0.181 | 0.917 |
| GFAP | 2670 | 0.150 | 0.917 |
| RANBP17 | 64901 | 0.205 | 0.916 |
| ANKAR | 150709 | 0.157 | 0.915 |
| NAPEPLD | 222236 | 0.151 | 0.914 |
| CDK10 | 8558 | -0.138 | 0.913 |
| CD14 | 929 | 0.013 | 0.912 |
| TCHP | 84260 | -0.172 | 0.909 |
| DSP | 1832 | -0.205 | 0.909 |
| PM20D1 | 148811 | 0.131 | 0.909 |
| ADAMTS4 | 9507 | -0.206 | 0.907 |
| CACNA2D1 | 781 | 0.152 | 0.905 |
| ELMSAN1 | 91748 | 0.149 | 0.905 |
| STAU2 | 27067 | -0.151 | 0.905 |
| RPRD2 | 23248 | -0.157 | 0.902 |
| FLG | 2312 | 0.148 | 0.902 |
| FANCC | 2176 | -0.192 | 0.899 |
| LRRC39 | 127495 | 0.292 | 0.897 |
| PALB2 | 79728 | 0.118 | 0.896 |
| MADCAM1 | 8174 | -0.140 | 0.896 |
| MYH8 | 4626 | 0.130 | 0.893 |
| EXOC5 | 10640 | 0.319 | 0.893 |
| DNM1L | 10059 | 0.163 | 0.893 |
| TOM1L2 | 146691 | 0.314 | 0.892 |
| SPTB | 6710 | -0.120 | 0.892 |
| MYO9B | 4650 | 0.265 | 0.892 |
| CNGB1 | 1258 | -0.155 | 0.890 |
| CFHR2 | 3080 | 0.410 | 0.890 |
| CRYGS | 1427 | -0.400 | 0.888 |
| FGA | 2243 | 0.056 | 0.888 |
| F11 | 2160 | 0.031 | 0.887 |
| TRHDE | 29953 | -0.138 | 0.887 |
| NEIL2 | 252969 | -0.156 | 0.887 |
| EME1 | 146956 | -0.246 | 0.886 |
| SH3D19 | 152503 | 0.345 | 0.886 |
| SLMAP | 7871 | -0.232 | 0.885 |
| KRT19 | 3880 | -0.236 | 0.885 |
| S100A8 | 6279 | 0.051 | 0.883 |
| KLHL42 | 57542 | 0.416 | 0.881 |
| LAMB1 | 3912 | 0.140 | 0.880 |
| SLC22A6 | 9356 | 0.312 | 0.880 |
| PRCP | 5547 | 0.141 | 0.879 |
| HAUS3 | 79441 | 0.345 | 0.876 |
| COG5 | 10466 | 0.320 | 0.876 |
| SPOCD1 | 90853 | 0.220 | 0.875 |
| LYZ | 4069 | -0.027 | 0.875 |
| CDH13 | 1012 | -0.037 | 0.872 |
| LILRB1 | 10859 | 0.269 | 0.872 |
| LRP1 | 4035 | -0.174 | 0.870 |
| ZNF544 | 27300 | 0.207 | 0.869 |
| RDX | 5962 | 0.143 | 0.868 |
| FABP5 | 2171 | 0.247 | 0.868 |
| KRT10 | 3858 | 0.064 | 0.865 |
| TUBB2A | 7280 | 0.168 | 0.865 |
| TUBB2B | 347733 | 0.168 | 0.863 |
| B4GALT1 | 2683 | -0.178 | 0.863 |
| ACTB | 60 | 0.044 | 0.860 |
| ACTG1 | 71 | 0.044 | 0.860 |
| B4GALT5 | 9334 | -0.178 | 0.860 |
| YWHAZ | 7534 | -0.375 | 0.860 |
| LILRA1 | 11024 | 0.341 | 0.859 |
| ALDOA | 226 | -0.156 | 0.858 |
| GNB1L | 54584 | -0.233 | 0.856 |
| PTPRG | 5793 | -0.307 | 0.853 |
| HBD | 3045 | -0.178 | 0.852 |
| RBM10 | 8241 | 0.288 | 0.851 |
| GOLGA6L1 | 283767 | 0.351 | 0.851 |
| LOC102723623 | 102723623 | 0.351 | 0.851 |
| CGN | 57530 | 0.350 | 0.849 |
| MDP1 | 145553 | 0.332 | 0.848 |
| LILRA2 | 11027 | 0.266 | 0.847 |
| KRT2 | 3849 | 0.079 | 0.845 |
| MYH6 | 4624 | -0.321 | 0.843 |
| NEDD8-MDP1 | 100528064 | 0.332 | 0.843 |
| CFI | 3426 | 0.013 | 0.843 |
| HYI | 81888 | -0.035 | 0.843 |
| TUBB8 | 347688 | 0.198 | 0.841 |
| GOLGA6L7 | 728310 | -0.466 | 0.838 |
| B2M | 567 | 0.068 | 0.837 |
| PSMB6 | 5694 | -0.032 | 0.836 |
| ASXL3 | 80816 | 0.215 | 0.836 |
| TUBB4A | 10382 | 0.198 | 0.836 |
| TUBB4B | 10383 | 0.198 | 0.836 |
| PDE6B | 5158 | 0.304 | 0.836 |
| RMND1 | 55005 | -0.297 | 0.835 |
| NES | 10763 | 0.434 | 0.832 |
| SERPINB3 | 6317 | 0.381 | 0.832 |
| MYH9 | 4627 | 0.131 | 0.831 |
| SYCE1 | 93426 | 0.277 | 0.826 |
| WFDC3 | 140686 | -0.766 | 0.818 |
| ANKRD62 | 342850 | 0.597 | 0.818 |
| CD163 | 9332 | -0.362 | 0.817 |
| SPIN1 | 10927 | 0.562 | 0.813 |
| CTBS | 1486 | 0.044 | 0.812 |
| PROZ | 8858 | -0.054 | 0.812 |
| KRT78 | 196374 | -0.596 | 0.807 |
| FAM20C | 56975 | -0.294 | 0.807 |
| SMCO1 | 255798 | -0.415 | 0.806 |
| HRNR | 388697 | 0.091 | 0.804 |
| ARR3 | 407 | -0.748 | 0.803 |
| ING1 | 3621 | 0.307 | 0.803 |
| DSG2 | 1829 | -0.452 | 0.801 |
| TFR2 | 7036 | 0.358 | 0.801 |
| PPIA | 5478 | 0.569 | 0.800 |
| ITPR2 | 3709 | -0.462 | 0.798 |
| DLEC1 | 9940 | 0.413 | 0.797 |
| TNC | 3371 | 0.345 | 0.796 |
| TF | 7018 | 0.017 | 0.795 |
| EIF2AK4 | 440275 | 0.486 | 0.795 |
| SERPINA3 | 12 | 0.021 | 0.795 |
| CDH8 | 1006 | 0.473 | 0.794 |
| PCSK9 | 255738 | 0.345 | 0.792 |
| C7 | 730 | 0.025 | 0.790 |
| MORC1 | 27136 | -0.548 | 0.790 |
| GSTM5 | 2949 | -0.089 | 0.787 |
| UBE2Q2 | 92912 | -0.433 | 0.783 |
| TUBB | 203068 | 0.466 | 0.779 |
| LILRA3 | 11026 | -0.452 | 0.778 |
| F5 | 2153 | 0.035 | 0.775 |
| SERPINA10 | 51156 | 0.029 | 0.771 |
| DHX30 | 22907 | 0.734 | 0.771 |
| L3MBTL4 | 91133 | 0.753 | 0.769 |
| ACTN2 | 88 | -0.541 | 0.768 |
| CFP | 5199 | 0.642 | 0.765 |
| ACO1 | 48 | 0.581 | 0.763 |
| KRT4 | 3851 | -0.958 | 0.762 |
| NRP1 | 8829 | 0.094 | 0.760 |
| CUL4B | 8450 | -0.428 | 0.759 |
| CROCC2 | 728763 | 0.704 | 0.759 |
| P4HB | 5034 | 0.598 | 0.758 |
| GPLD1 | 2822 | 0.040 | 0.755 |
| SCEL | 8796 | -0.488 | 0.755 |
| CP | 1356 | 0.018 | 0.753 |
| FLNA | 2316 | 0.496 | 0.752 |
| RANBP2 | 5903 | 0.347 | 0.751 |
| ANG | 283 | 0.740 | 0.750 |
| FETUB | 26998 | -0.049 | 0.748 |
| CDHR2 | 54825 | -0.317 | 0.747 |
| DDAH1 | 23576 | 0.545 | 0.745 |
| CHN1 | 1123 | -0.793 | 0.741 |
| KRT6C | 286887 | 0.931 | 0.740 |
| CTSH | 1512 | 0.679 | 0.737 |
| ALCAM | 214 | -0.688 | 0.736 |
| ADAMTSL3 | 57188 | -0.790 | 0.733 |
| TMF1 | 7110 | -0.614 | 0.729 |
| MYH15 | 22989 | -0.368 | 0.728 |
| F8 | 2157 | -0.392 | 0.727 |
| LTF | 4057 | -0.412 | 0.727 |
| HPCAL4 | 51440 | 1.213 | 0.727 |
| DPEP2 | 64174 | 0.517 | 0.725 |
| ADIPOQ | 9370 | -0.372 | 0.723 |
| KIF27 | 55582 | 0.622 | 0.717 |
| MPO | 4353 | -0.404 | 0.716 |
| HSP90B1 | 7184 | 0.383 | 0.715 |
| RBM6 | 10180 | 1.091 | 0.714 |
| RNH1 | 6050 | 0.811 | 0.713 |
| PRR4 | 11272 | 0.690 | 0.711 |
| BST1 | 683 | 0.719 | 0.711 |
| TEPSIN | 146705 | -0.728 | 0.710 |
| SH2B3 | 10019 | -0.601 | 0.710 |
| CFB | 629 | 0.027 | 0.710 |
| SERPINB4 | 6318 | -0.565 | 0.708 |
| PRSS1 | 5644 | -0.128 | 0.705 |
| HLA-A | 3105 | -0.703 | 0.704 |
| POSTN | 10631 | -0.622 | 0.703 |
| FRMPD1 | 22844 | -0.749 | 0.702 |
| TTLL7 | 79739 | -0.867 | 0.702 |
| EXT2 | 2132 | 0.452 | 0.699 |
| HBG1 | 3047 | 0.990 | 0.699 |
| FSTL4 | 23105 | 0.610 | 0.698 |
| KRT80 | 144501 | 0.963 | 0.698 |
| IGF2R | 3482 | 0.560 | 0.696 |
| ACE | 1636 | 0.483 | 0.695 |
| CFL1 | 1072 | -0.712 | 0.694 |
| BCHE | 590 | 0.049 | 0.692 |
| S100A9 | 6280 | 0.128 | 0.692 |
| NFKB1 | 4790 | -0.483 | 0.692 |
| MACF1 | 23499 | 0.583 | 0.691 |
| KPRP | 448834 | -0.705 | 0.691 |
| MB | 4151 | -0.630 | 0.685 |
| C1QTNF3 | 114899 | 0.730 | 0.684 |
| TRIM71 | 131405 | -1.054 | 0.684 |
| HBG2 | 3048 | 1.042 | 0.683 |
| ZNF93 | 81931 | -0.858 | 0.682 |
| C9 | 735 | 0.032 | 0.682 |
| MINPP1 | 9562 | 0.629 | 0.678 |
| FASTKD3 | 79072 | -0.920 | 0.678 |
| PROS1 | 5627 | 0.031 | 0.677 |
| KRT5 | 3852 | -0.161 | 0.677 |
| SERPINA7 | 6906 | 0.035 | 0.675 |
| CFAP58 | 159686 | 0.718 | 0.674 |
| URB1 | 9875 | 0.846 | 0.673 |
| TUB | 7275 | -0.837 | 0.673 |
| SH3BGRL3 | 83442 | -0.879 | 0.673 |
| E2F5 | 1875 | 0.655 | 0.672 |
| CTSD | 1509 | 0.504 | 0.671 |
| VCAM1 | 7412 | 0.114 | 0.670 |
| CETP | 1071 | -0.424 | 0.670 |
| GLIPR2 | 152007 | 0.928 | 0.669 |
| KRT6A | 3853 | -0.836 | 0.669 |
| RASSF10 | 644943 | 1.141 | 0.668 |
| KRT27 | 342574 | -1.688 | 0.668 |
| ZHX2 | 22882 | 0.745 | 0.666 |
| ITGA8 | 8516 | 0.592 | 0.665 |
| DPP4 | 1803 | 0.753 | 0.663 |
| JUP | 3728 | 0.519 | 0.663 |
| FCGBP | 8857 | 0.456 | 0.662 |
| APOA2 | 336 | -0.056 | 0.661 |
| HEG1 | 57493 | 0.449 | 0.659 |
| C1orf56 | 54964 | 0.135 | 0.658 |
| GGH | 8836 | 0.095 | 0.657 |
| SCG3 | 29106 | -0.709 | 0.657 |
| PRRC2C | 23215 | 0.510 | 0.656 |
| RGS6 | 9628 | -0.994 | 0.653 |
| ARHGEF2 | 9181 | 0.878 | 0.650 |
| MYOC | 4653 | 0.881 | 0.650 |
| KRT15 | 3866 | -1.106 | 0.648 |
| ANXA6 | 309 | -0.980 | 0.648 |
| NID1 | 4811 | 0.802 | 0.647 |
| HELB | 92797 | 0.782 | 0.647 |
| LECT2 | 3950 | -0.955 | 0.647 |
| HPX | 3263 | 0.043 | 0.645 |
| HMOX2 | 3163 | 1.219 | 0.644 |
| ANKRD36 | 375248 | -0.470 | 0.643 |
| SPARCL1 | 8404 | 0.153 | 0.643 |
| FBLN1 | 2192 | -0.093 | 0.642 |
| APOA5 | 116519 | 0.602 | 0.642 |
| THBS3 | 7059 | 0.514 | 0.642 |
| KRT83 | 3889 | 0.653 | 0.641 |
| TPM3 | 7170 | 0.795 | 0.640 |
| PGLYRP2 | 114770 | 0.049 | 0.640 |
| KRT85 | 3891 | 0.674 | 0.640 |
| VASN | 114990 | -0.405 | 0.639 |
| KIAA1549L | 25758 | 0.560 | 0.639 |
| CTNNBIP1 | 56998 | 0.998 | 0.635 |
| HIST1H1D | 3007 | -0.479 | 0.634 |
| NUP153 | 9972 | 1.188 | 0.634 |
| CA2 | 760 | 1.147 | 0.631 |
| ANXA2 | 302 | -0.764 | 0.630 |
| NUP98 | 4928 | -0.623 | 0.628 |
| INHBC | 3626 | -1.028 | 0.627 |
| MAN2A1 | 4124 | -0.740 | 0.627 |
| CDH5 | 1003 | -0.058 | 0.627 |
| PTGFRN | 5738 | -0.878 | 0.626 |
| PLEKHG1 | 57480 | 1.257 | 0.626 |
| DNHD1 | 144132 | 0.815 | 0.626 |
| DNAH6 | 1768 | -0.746 | 0.625 |
| ADO | 84890 | -0.876 | 0.624 |
| CEP126 | 57562 | -0.857 | 0.623 |
| FAM160B1 | 57700 | -1.334 | 0.623 |
| ODF2L | 57489 | 0.758 | 0.621 |
| AMY1A | 276 | 0.591 | 0.619 |
| AMY1B | 277 | 0.591 | 0.619 |
| AMY1C | 278 | 0.591 | 0.619 |
| AMY2A | 279 | 0.591 | 0.619 |
| AMY2B | 280 | 0.591 | 0.619 |
| ZNF280D | 54816 | -1.076 | 0.618 |
| TAF1L | 138474 | 0.466 | 0.617 |
| UACA | 55075 | 0.652 | 0.617 |
| HERC2 | 8924 | -0.806 | 0.616 |
| MXD4 | 10608 | 1.289 | 0.616 |
| EXTL2 | 2135 | 0.892 | 0.615 |
| PPL | 5493 | -0.713 | 0.614 |
| NTN4 | 59277 | -0.941 | 0.613 |
| ATRN | 8455 | 0.038 | 0.612 |
| HSPA5 | 3309 | 0.090 | 0.612 |
| CRYBG3 | 131544 | 0.668 | 0.611 |
| TPI1 | 7167 | 0.642 | 0.611 |
| PATL1 | 219988 | 1.235 | 0.611 |
| TTN | 7273 | 0.743 | 0.611 |
| PRDM9 | 56979 | 0.648 | 0.610 |
| DIAPH1 | 1729 | 0.922 | 0.610 |
| SPDYA | 245711 | -1.082 | 0.609 |
| CFH | 3075 | 0.040 | 0.609 |
| PROC | 5624 | -0.095 | 0.607 |
| RALGAPB | 57148 | 0.762 | 0.606 |
| CST6 | 1474 | -0.853 | 0.605 |
| PON3 | 5446 | -0.039 | 0.605 |
| ADA2 | 51816 | -0.922 | 0.603 |
| PODXL | 5420 | 0.798 | 0.603 |
| LRRC36 | 55282 | 0.730 | 0.602 |
| ASPSCR1 | 79058 | -1.045 | 0.602 |
| GALNT17 | 64409 | 0.142 | 0.601 |
| NUP188 | 23511 | 0.985 | 0.600 |
| KRT31 | 3881 | 1.016 | 0.599 |
| AXL | 558 | 0.403 | 0.599 |
| CCDC68 | 80323 | 1.140 | 0.598 |
| CFHR3 | 10878 | 1.229 | 0.598 |
| HOMER2 | 9455 | -1.189 | 0.598 |
| APOD | 347 | -0.060 | 0.597 |
| FN1 | 2335 | -0.097 | 0.597 |
| RNASE4 | 6038 | -0.947 | 0.596 |
| DBH | 1621 | 0.572 | 0.593 |
| CFL2 | 1073 | -0.722 | 0.591 |
| MMRN2 | 79812 | -0.848 | 0.591 |
| ANXA1 | 301 | -0.499 | 0.591 |
| PRR11 | 55771 | -0.127 | 0.590 |
| PRELID2 | 153768 | -0.844 | 0.589 |
| KRT72 | 140807 | -1.283 | 0.588 |
| C1RL | 51279 | -0.060 | 0.588 |
| KNG1 | 3827 | 0.037 | 0.587 |
| MTPN | 136319 | -0.895 | 0.587 |
| MASP1 | 5648 | -0.100 | 0.586 |
| TGFBI | 7045 | 0.056 | 0.585 |
| GOPC | 57120 | 1.128 | 0.585 |
| CNTLN | 54875 | -0.794 | 0.584 |
| NIN | 51199 | -0.745 | 0.584 |
| C1QC | 714 | 0.066 | 0.582 |
| CCDC88A | 55704 | -0.762 | 0.581 |
| AMBP | 259 | 0.053 | 0.581 |
| CASR | 846 | -0.860 | 0.580 |
| PTGDS | 5730 | 0.095 | 0.579 |
| AFM | 173 | 0.066 | 0.579 |
| ALDOB | 229 | -0.416 | 0.579 |
| PLEKHD1 | 400224 | -1.065 | 0.576 |
| ORM2 | 5005 | 0.072 | 0.574 |
| TDRD7 | 23424 | -0.898 | 0.574 |
| GOLGA8M | 653720 | -1.083 | 0.573 |
| IGF1 | 3479 | -0.989 | 0.570 |
| PRAP1 | 118471 | -1.158 | 0.569 |
| C19orf54 | 284325 | -1.285 | 0.569 |
| H6PD | 9563 | -0.815 | 0.567 |
| PHF10 | 55274 | 1.520 | 0.567 |
| RAP1B | 5908 | -0.776 | 0.563 |
| EVPL | 2125 | -1.158 | 0.563 |
| RAP1A | 5906 | -0.760 | 0.563 |
| MEGF8 | 1954 | 0.945 | 0.562 |
| BNIP2 | 663 | -0.628 | 0.562 |
| LARP1B | 55132 | 0.763 | 0.562 |
| GBF1 | 8729 | 0.841 | 0.561 |
| FKBP1A | 2280 | -0.918 | 0.561 |
| LRBA | 987 | -0.886 | 0.559 |
| OSBPL2 | 9885 | 0.791 | 0.558 |
| ANKRD24 | 170961 | 0.957 | 0.557 |
| FBXL22 | 283807 | 0.024 | 0.557 |
| OPTN | 10133 | -0.709 | 0.555 |
| FAM214A | 56204 | -0.606 | 0.551 |
| CLIC3 | 9022 | 0.805 | 0.550 |
| LZTS3 | 9762 | -0.514 | 0.550 |
| MTM1 | 4534 | 0.916 | 0.547 |
| CCDC30 | 728621 | 1.134 | 0.547 |
| ARFGEF3 | 57221 | 0.785 | 0.544 |
| MAFG | 4097 | -1.607 | 0.544 |
| PIP4K2C | 79837 | 1.685 | 0.543 |
| IGLL5 | 100423062 | -0.123 | 0.543 |
| NFE2L2 | 4780 | -1.742 | 0.542 |
| SPTBN2 | 6712 | 0.859 | 0.540 |
| HLA-B | 3106 | -1.287 | 0.539 |
| CKAP4 | 10970 | 0.802 | 0.539 |
| CPN2 | 1370 | 0.042 | 0.539 |
| C4BPA | 722 | 0.064 | 0.538 |
| CRISP2 | 7180 | 0.794 | 0.537 |
| ITPR3 | 3710 | 0.684 | 0.536 |
| ATXN2L | 11273 | -0.784 | 0.536 |
| ICAM2 | 3384 | -1.065 | 0.536 |
| SYNJ2 | 8871 | 0.973 | 0.535 |
| ICK | 22858 | -0.986 | 0.535 |
| PTPRS | 5802 | 1.007 | 0.535 |
| CA1 | 759 | -0.403 | 0.534 |
| UTS2 | 10911 | 1.134 | 0.534 |
| SPINK5 | 11005 | -0.666 | 0.533 |
| ADGRF4 | 221393 | -0.819 | 0.533 |
| ERBB4 | 2066 | -1.068 | 0.532 |
| RGL4 | 266747 | 1.392 | 0.532 |
| MRC1 | 4360 | -0.299 | 0.532 |
| MET | 4233 | -0.716 | 0.531 |
| SHPRH | 257218 | 1.438 | 0.530 |
| SAA2-SAA4 | 100528017 | 0.077 | 0.530 |
| RBBP8 | 5932 | 1.751 | 0.524 |
| SLC44A3 | 126969 | -0.980 | 0.524 |
| FCN3 | 8547 | 0.042 | 0.523 |
| KMO | 8564 | -1.021 | 0.523 |
| ANKRD29 | 147463 | 0.905 | 0.523 |
| CIT | 11113 | 0.648 | 0.522 |
| ASCC3 | 10973 | 1.101 | 0.521 |
| SEMA4F | 10505 | 1.044 | 0.519 |
| DRICH1 | 51233 | -1.003 | 0.518 |
| SYNM | 23336 | 1.634 | 0.518 |
| GNPTG | 84572 | -1.368 | 0.517 |
| F9 | 2158 | 0.050 | 0.516 |
| LDHB | 3945 | 0.119 | 0.514 |
| TSNARE1 | 203062 | -1.366 | 0.514 |
| ADAMDEC1 | 27299 | 0.547 | 0.512 |
| PRKG2 | 5593 | 1.818 | 0.511 |
| ITIH3 | 3699 | -0.044 | 0.510 |
| SLC38A10 | 124565 | -0.378 | 0.510 |
| ADAM30 | 11085 | 0.686 | 0.509 |
| ORM1 | 5004 | -0.145 | 0.508 |
| THBS4 | 7060 | -0.916 | 0.508 |
| RHOXF1 | 158800 | -1.770 | 0.505 |
| PZP | 5858 | -0.541 | 0.503 |
| SLC27A5 | 10998 | -1.223 | 0.501 |
| LBP | 3929 | 0.083 | 0.500 |
| CAT | 847 | 1.226 | 0.498 |
| GC | 2638 | -0.058 | 0.496 |
| CSF1R | 1436 | -0.237 | 0.493 |
| GFRA1 | 2674 | 2.223 | 0.487 |
| GP1BA | 2811 | 0.164 | 0.486 |
| DCD | 117159 | -0.321 | 0.483 |
| BRWD1 | 54014 | -0.823 | 0.483 |
| CASP14 | 23581 | -1.412 | 0.483 |
| CD99 | 4267 | 1.815 | 0.481 |
| PAFAH1B2 | 5049 | 1.084 | 0.481 |
| APC | 324 | -0.845 | 0.480 |
| OGN | 4969 | 1.433 | 0.478 |
| MYO1E | 4643 | -1.505 | 0.477 |
| PKD1 | 5310 | 0.796 | 0.476 |
| PRDX1 | 5052 | -1.354 | 0.475 |
| PGK1 | 5230 | -0.928 | 0.475 |
| MYO15A | 51168 | -0.848 | 0.474 |
| GAPDH | 2597 | -0.859 | 0.473 |
| RNF123 | 63891 | 1.486 | 0.473 |
| MBL2 | 4153 | -0.718 | 0.472 |
| SLC9A3R1 | 9368 | 1.105 | 0.472 |
| DST | 667 | 0.626 | 0.471 |
| ENG | 2022 | -0.555 | 0.471 |
| CHMP4A | 29082 | 0.121 | 0.471 |
| TGM3 | 7053 | -1.606 | 0.470 |
| MYO1F | 4542 | -1.497 | 0.470 |
| PI16 | 221476 | -0.066 | 0.470 |
| SLC2A2 | 6514 | -1.961 | 0.470 |
| XRCC5 | 7520 | 1.137 | 0.469 |
| BTD | 686 | 0.092 | 0.467 |
| RUFY3 | 22902 | -1.219 | 0.467 |
| C1R | 715 | 0.069 | 0.467 |
| ABHD14A-ACY1 | 100526760 | -0.468 | 0.464 |
| LTBP1 | 4052 | 1.307 | 0.463 |
| TAGLN2 | 8407 | -1.714 | 0.461 |
| MASP2 | 10747 | 0.086 | 0.460 |
| MAEA | 10296 | -1.176 | 0.460 |
| PTPRF | 5792 | 1.114 | 0.460 |
| ECM1 | 1893 | -0.171 | 0.457 |
| ACY1 | 95 | -0.503 | 0.456 |
| APOH | 350 | 0.105 | 0.451 |
| SERPINF2 | 5345 | 0.077 | 0.449 |
| APEH | 327 | 1.100 | 0.449 |
| KANSL1 | 284058 | 1.403 | 0.447 |
| YWHAG | 7532 | 1.201 | 0.446 |
| DHX8 | 1659 | -2.489 | 0.443 |
| LRG1 | 116844 | -0.080 | 0.442 |
| VCP | 7415 | -0.962 | 0.438 |
| CCDC38 | 120935 | 1.231 | 0.437 |
| CRISP3 | 10321 | -1.217 | 0.435 |
| DMKN | 93099 | -1.794 | 0.434 |
| HBA1 | 3039 | -0.371 | 0.434 |
| HBA2 | 3040 | -0.371 | 0.434 |
| TENM3 | 55714 | -0.898 | 0.433 |
| SERPINC1 | 462 | 0.071 | 0.433 |
| HABP2 | 3026 | 0.104 | 0.431 |
| HBB | 3043 | -0.355 | 0.430 |
| VCL | 7414 | 1.176 | 0.428 |
| ERICH1 | 157697 | -1.348 | 0.428 |
| FLG2 | 388698 | -1.257 | 0.428 |
| PROCR | 10544 | 0.834 | 0.426 |
| TFRC | 7037 | 1.375 | 0.426 |
| PEPD | 5184 | 1.194 | 0.425 |
| CALML5 | 51806 | 1.705 | 0.421 |
| CD44 | 960 | 0.709 | 0.418 |
| C1S | 716 | 0.057 | 0.418 |
| CENPF | 1063 | -2.150 | 0.418 |
| TIMP1 | 7076 | 1.985 | 0.416 |
| MYO9A | 4649 | 0.576 | 0.416 |
| SUV39H1 | 6839 | 0.916 | 0.415 |
| GOLM1 | 51280 | -1.539 | 0.415 |
| ALB | 213 | -0.250 | 0.413 |
| MARCO | 8685 | -0.985 | 0.410 |
| AOC3 | 8639 | 1.668 | 0.405 |
| CEP164 | 22897 | 1.648 | 0.402 |
| ADCY10 | 55811 | 1.560 | 0.402 |
| F2 | 2147 | 0.061 | 0.401 |
| SELENOP | 6414 | 0.126 | 0.400 |
| IGFBP2 | 3485 | 0.287 | 0.399 |
| COMP | 1311 | 0.136 | 0.399 |
| CBFA2T3 | 863 | -0.378 | 0.399 |
| CBFA2T2 | 9139 | -0.379 | 0.398 |
| ANPEP | 290 | -0.179 | 0.398 |
| RUNDC3A | 10900 | -0.687 | 0.398 |
| RUNX1T1 | 862 | -0.388 | 0.397 |
| ROCK2 | 9475 | -1.607 | 0.397 |
| ADGRG6 | 57211 | 1.144 | 0.396 |
| PPFIA3 | 8541 | -0.768 | 0.396 |
| EPPK1 | 83481 | 1.136 | 0.394 |
| CTSS | 1520 | -1.394 | 0.392 |
| SGSM2 | 9905 | -1.384 | 0.391 |
| IL1RAP | 3556 | 0.793 | 0.391 |
| KTN1 | 3895 | 1.206 | 0.390 |
| FLOT1 | 10211 | -0.693 | 0.390 |
| KMT2D | 8085 | 1.085 | 0.389 |
| ISLR | 3671 | -0.653 | 0.388 |
| PTER | 9317 | 2.351 | 0.387 |
| BASP1 | 10409 | -1.412 | 0.385 |
| KLKB1 | 3818 | 0.080 | 0.384 |
| CFAP57 | 149465 | 1.482 | 0.383 |
| LOC102723407 | 102723407 | 2.064 | 0.383 |
| TRPS1 | 7227 | 1.199 | 0.383 |
| VPS4A | 27183 | 3.560 | 0.382 |
| IL6ST | 3572 | -0.688 | 0.381 |
| CBLN4 | 140689 | 1.673 | 0.379 |
| MGP | 4256 | -0.979 | 0.378 |
| IQUB | 154865 | -1.777 | 0.378 |
| NRG2 | 9542 | -1.794 | 0.377 |
| PTPMT1 | 114971 | -0.711 | 0.376 |
| HIPK1 | 204851 | 0.555 | 0.376 |
| C8B | 732 | 0.080 | 0.376 |
| VPS18 | 57617 | -0.811 | 0.376 |
| PRG4 | 10216 | 0.191 | 0.374 |
| CFHR5 | 81494 | 0.223 | 0.371 |
| CNTN1 | 1272 | 1.340 | 0.371 |
| FLNC | 2318 | 1.175 | 0.369 |
| CDK18 | 5129 | -1.382 | 0.367 |
| TPM2 | 7169 | -0.881 | 0.367 |
| TRIM4 | 89122 | -2.081 | 0.367 |
| EZR | 7430 | -1.792 | 0.367 |
| SLC4A1 | 6521 | -1.440 | 0.364 |
| GPX3 | 2878 | 0.110 | 0.363 |
| TMEM201 | 199953 | 2.649 | 0.362 |
| WWP1 | 11059 | 1.588 | 0.362 |
| PHF21A | 51317 | 0.110 | 0.362 |
| ATP6V0A4 | 50617 | 1.497 | 0.361 |
| HAUS5 | 23354 | 2.083 | 0.360 |
| APMAP | 57136 | 0.273 | 0.359 |
| PEBP4 | 157310 | 1.769 | 0.359 |
| CDC23 | 8697 | -1.596 | 0.357 |
| CEACAM20 | 125931 | -1.772 | 0.357 |
| C7orf25 | 79020 | 2.076 | 0.355 |
| FARP1 | 10160 | 1.144 | 0.354 |
| LOC110384692 | 110384692 | -1.688 | 0.353 |
| ZNF550 | 162972 | -1.012 | 0.352 |
| RSPH14 | 27156 | -1.758 | 0.350 |
| CGNL1 | 84952 | -2.202 | 0.349 |
| PVR | 5817 | -0.207 | 0.349 |
| HBE1 | 3046 | 1.411 | 0.348 |
| QSER1 | 79832 | -1.792 | 0.348 |
| CR2 | 1380 | 1.137 | 0.346 |
| HSFX4 | 101927685 | 1.407 | 0.345 |
| HSFX3 | 101928917 | 1.407 | 0.345 |
| CTTNBP2 | 83992 | 1.423 | 0.345 |
| KRT86 | 3892 | 1.610 | 0.344 |
| SLC12A5 | 57468 | 1.155 | 0.344 |
| KRT81 | 3887 | 1.629 | 0.344 |
| NRIP1 | 8204 | 0.797 | 0.344 |
| PLXNB1 | 5364 | 1.265 | 0.343 |
| LOC101927506 | 101927506 | -1.749 | 0.342 |
| GSPT2 | 23708 | 1.098 | 0.342 |
| CD5L | 922 | -1.005 | 0.337 |
| KRT16 | 3868 | -1.263 | 0.337 |
| TPR | 7175 | -1.794 | 0.336 |
| IARS | 3376 | -1.175 | 0.336 |
| SLC4A10 | 57282 | 0.001 | 0.336 |
| TNFSF18 | 8995 | -0.064 | 0.336 |
| QTRT2 | 79691 | -0.044 | 0.336 |
| ZPR1 | 8882 | 0.058 | 0.336 |
| IGFBP1 | 3484 | 0.165 | 0.336 |
| TPM1 | 7168 | 0.056 | 0.336 |
| NOTCH1 | 4851 | -0.142 | 0.336 |
| FGG | 2266 | -0.430 | 0.336 |
| MYH3 | 4621 | 0.265 | 0.336 |
| ITPRIP | 85450 | 0.361 | 0.336 |
| ACTG2 | 72 | 1.004 | 0.336 |
| CES1 | 1066 | -0.938 | 0.336 |
| GALNT2 | 2590 | 0.652 | 0.336 |
| NUCB1 | 4924 | -0.802 | 0.336 |
| PLEK | 5341 | -0.976 | 0.336 |
| NUP205 | 23165 | -1.188 | 0.336 |
| NUP62 | 23636 | -0.985 | 0.336 |
| OLA1 | 29789 | 0.954 | 0.336 |
| FRYL | 285527 | 0.651 | 0.336 |
| TMEM189-UBE2V1 | 387522 | -0.713 | 0.336 |
| ABCB7 | 22 | 0.804 | 0.336 |
| ACTA2 | 59 | 0.996 | 0.336 |
| ARG1 | 383 | -0.844 | 0.336 |
| CALCB | 797 | 1.060 | 0.336 |
| CHIT1 | 1118 | -0.732 | 0.336 |
| CTSB | 1508 | 0.793 | 0.336 |
| FCN1 | 2219 | 1.079 | 0.336 |
| GDI2 | 2665 | 0.687 | 0.336 |
| GLUL | 2752 | 0.738 | 0.336 |
| GSS | 2937 | -0.668 | 0.336 |
| L1CAM | 3897 | 0.591 | 0.336 |
| LMO2 | 4005 | 0.803 | 0.336 |
| MAK | 4117 | 0.554 | 0.336 |
| MTNR1A | 4543 | 1.237 | 0.336 |
| MYO1A | 4640 | 0.487 | 0.336 |
| NOTCH3 | 4854 | 0.808 | 0.336 |
| CNTN3 | 5067 | 0.674 | 0.336 |
| PDK1 | 5163 | -1.263 | 0.336 |
| PRG2 | 5553 | 0.761 | 0.336 |
| RAD23A | 5886 | -0.703 | 0.336 |
| RAD23B | 5887 | -0.721 | 0.336 |
| RNASE2 | 6036 | -0.923 | 0.336 |
| RNASEL | 6041 | -1.111 | 0.336 |
| S100A4 | 6275 | -1.012 | 0.336 |
| SLC12A4 | 6560 | -0.401 | 0.336 |
| NEK4 | 6787 | 0.066 | 0.336 |
| YWHAE | 7531 | 1.030 | 0.336 |
| RIOK3 | 8780 | -1.108 | 0.336 |
| KRT75 | 9119 | 1.140 | 0.336 |
| COX5A | 9377 | 0.832 | 0.336 |
| ATP9A | 10079 | -0.514 | 0.336 |
| TRAP1 | 10131 | -0.654 | 0.336 |
| CPQ | 10404 | 0.699 | 0.336 |
| YWHAQ | 10971 | -0.874 | 0.336 |
| B4GAT1 | 11041 | -0.920 | 0.336 |
| PCSK1N | 27344 | -0.645 | 0.336 |
| GP6 | 51206 | 0.781 | 0.336 |
| SETD4 | 54093 | -1.165 | 0.336 |
| KRT20 | 54474 | -0.902 | 0.336 |
| MRM3 | 55178 | -1.182 | 0.336 |
| SCYL2 | 55681 | 1.048 | 0.336 |
| RNF17 | 56163 | 0.672 | 0.336 |
| CIAPIN1 | 57019 | -0.509 | 0.336 |
| METTL14 | 57721 | 0.590 | 0.336 |
| NLRC4 | 58484 | -0.913 | 0.336 |
| IL21 | 59067 | 1.051 | 0.336 |
| ATP8B4 | 79895 | -0.702 | 0.336 |
| CSMD2 | 114784 | -0.843 | 0.336 |
| SDR9C7 | 121214 | 0.940 | 0.336 |
| CNTNAP5 | 129684 | 0.874 | 0.336 |
| CTCFL | 140690 | -1.029 | 0.336 |
| RIPOR3 | 140876 | -0.832 | 0.336 |
| CCDC13 | 152206 | 0.813 | 0.336 |
| RNF217 | 154214 | 0.951 | 0.336 |
| SMCO2 | 341346 | 0.798 | 0.336 |
| ACTBL2 | 345651 | -1.203 | 0.336 |
| ANKRD36C | 400986 | -0.546 | 0.336 |
| RGPD3 | 653489 | 0.606 | 0.336 |
| LOC107987462 | 107987462 | 0.460 | 0.336 |
| ACHE | 43 | 0.618 | 0.336 |
| ALDH7A1 | 501 | -0.979 | 0.336 |
| ATP6V0A1 | 535 | -0.755 | 0.336 |
| COL1A2 | 1278 | 0.474 | 0.336 |
| DDT | 1652 | -0.798 | 0.336 |
| EN1 | 2019 | -1.131 | 0.336 |
| F13B | 2165 | 0.861 | 0.336 |
| MTOR | 2475 | 0.714 | 0.336 |
| GSR | 2936 | -0.721 | 0.336 |
| HIST1H1C | 3006 | -0.882 | 0.336 |
| HIST1H1E | 3008 | -0.882 | 0.336 |
| HIST1H1A | 3024 | -0.905 | 0.336 |
| IGLL1 | 3543 | -0.839 | 0.336 |
| MDH1 | 4190 | -0.831 | 0.336 |
| MMP11 | 4320 | 0.736 | 0.336 |
| MX2 | 4600 | -0.754 | 0.336 |
| MYH4 | 4622 | -0.587 | 0.336 |
| MYL1 | 4632 | 0.765 | 0.336 |
| OMD | 4958 | -0.541 | 0.336 |
| CTSA | 5476 | -0.761 | 0.336 |
| PRKAR2B | 5577 | -0.844 | 0.336 |
| PTPN4 | 5775 | 0.635 | 0.336 |
| SHH | 6469 | 0.701 | 0.336 |
| SPP1 | 6696 | -0.910 | 0.336 |
| TSTA3 | 7264 | -0.717 | 0.336 |
| TULP2 | 7288 | -1.039 | 0.336 |
| YWHAB | 7529 | -0.909 | 0.336 |
| CPNE1 | 8904 | -0.722 | 0.336 |
| IQCB1 | 9657 | 0.775 | 0.336 |
| DOCK4 | 9732 | 0.653 | 0.336 |
| STARD8 | 9754 | 0.763 | 0.336 |
| CCS | 9973 | -0.747 | 0.336 |
| SLC12A6 | 9990 | -0.405 | 0.336 |
| LILRB2 | 10288 | 1.003 | 0.336 |
| LILRB3 | 11025 | 0.457 | 0.336 |
| AP4S1 | 11154 | 0.823 | 0.336 |
| PARK7 | 11315 | -0.779 | 0.336 |
| CNKSR2 | 22866 | -0.827 | 0.336 |
| MON2 | 23041 | -0.897 | 0.336 |
| CORO1C | 23603 | -0.738 | 0.336 |
| NECTIN3 | 25945 | -0.925 | 0.336 |
| FGFR1OP2 | 26127 | -0.863 | 0.336 |
| AKAP8L | 26993 | -0.894 | 0.336 |
| AFF4 | 27125 | -1.114 | 0.336 |
| ELOA2 | 51224 | 0.802 | 0.336 |
| KRT76 | 51350 | 1.201 | 0.336 |
| RSF1 | 51773 | -1.101 | 0.336 |
| C21orf58 | 54058 | 1.003 | 0.336 |
| RC3H2 | 54542 | 0.639 | 0.336 |
| CWC25 | 54883 | -0.830 | 0.336 |
| PI4K2A | 55361 | 0.808 | 0.336 |
| FAR2 | 55711 | -0.993 | 0.336 |
| NT5M | 56953 | -0.900 | 0.336 |
| LSM2 | 57819 | 0.736 | 0.336 |
| NEUROD6 | 63974 | 0.915 | 0.336 |
| EPB41L4A | 64097 | 0.782 | 0.336 |
| CTAGE1 | 64693 | -0.809 | 0.336 |
| APOL3 | 80833 | 0.667 | 0.336 |
| APH1B | 83464 | 1.098 | 0.336 |
| DDX59 | 83479 | 0.489 | 0.336 |
| NUF2 | 83540 | -0.940 | 0.336 |
| N4BP2L1 | 90634 | 1.204 | 0.336 |
| PHACTR1 | 221692 | -0.778 | 0.336 |
| C9orf43 | 257169 | 0.887 | 0.336 |
| VMO1 | 284013 | 0.901 | 0.336 |
| TTC6 | 319089 | -0.766 | 0.336 |
| S100A7A | 338324 | -0.906 | 0.336 |
| OR5K4 | 403278 | 0.962 | 0.336 |
| FER1L6 | 654463 | -0.750 | 0.336 |
| CD300LD | 100131439 | -1.268 | 0.336 |
| LOC105373381 | 105373381 | -1.175 | 0.336 |
| ACP1 | 52 | -0.701 | 0.336 |
| ABCC6 | 368 | -0.775 | 0.336 |
| ATP1B3 | 483 | 0.783 | 0.336 |
| BLVRA | 644 | 0.827 | 0.336 |
| CNTFR | 1271 | 0.704 | 0.336 |
| COL1A1 | 1277 | 0.608 | 0.336 |
| CSPG4 | 1464 | -0.735 | 0.336 |
| FUT8 | 2530 | 1.015 | 0.336 |
| SFN | 2810 | -0.939 | 0.336 |
| GTF2E1 | 2960 | -0.841 | 0.336 |
| HIST1H1B | 3009 | -0.872 | 0.336 |
| HSPA1A | 3303 | -0.511 | 0.336 |
| HSPA1B | 3304 | -0.511 | 0.336 |
| IDH3A | 3419 | -0.766 | 0.336 |
| KRT3 | 3850 | -0.616 | 0.336 |
| LGALS7 | 3963 | -0.978 | 0.336 |
| LIPC | 3990 | 0.488 | 0.336 |
| MYH1 | 4619 | -0.579 | 0.336 |
| NEO1 | 4756 | -0.631 | 0.336 |
| PEBP1 | 5037 | -0.919 | 0.336 |
| PDGFRB | 5159 | 0.608 | 0.336 |
| PECAM1 | 5175 | 0.549 | 0.336 |
| PGD | 5226 | 0.690 | 0.336 |
| PPID | 5481 | -0.788 | 0.336 |
| PRKAB2 | 5565 | -0.628 | 0.336 |
| PXN | 5829 | 0.901 | 0.336 |
| RPA1 | 6117 | -1.002 | 0.336 |
| RPL6 | 6128 | 0.795 | 0.336 |
| SMTN | 6525 | 0.840 | 0.336 |
| SMARCC1 | 6599 | -0.548 | 0.336 |
| SREBF2 | 6721 | -1.039 | 0.336 |
| SUPT6H | 6830 | -0.892 | 0.336 |
| SYT5 | 6861 | 0.863 | 0.336 |
| UBE2V1 | 7335 | -0.806 | 0.336 |
| SCGB1A1 | 7356 | -0.916 | 0.336 |
| PKP4 | 8502 | -0.721 | 0.336 |
| MYH13 | 8735 | -0.579 | 0.336 |
| SIGLEC5 | 8778 | -0.834 | 0.336 |
| DGKI | 9162 | -0.796 | 0.336 |
| RAD50 | 10111 | 0.602 | 0.336 |
| C11orf58 | 10944 | -0.779 | 0.336 |
| WDFY3 | 23001 | 0.360 | 0.336 |
| TMCC1 | 23023 | -0.658 | 0.336 |
| AGTPBP1 | 23287 | -0.675 | 0.336 |
| DNAH1 | 25981 | -0.773 | 0.336 |
| DNAAF2 | 55172 | -0.534 | 0.336 |
| CD248 | 57124 | 0.607 | 0.336 |
| SH3RF1 | 57630 | -0.589 | 0.336 |
| DMRTA1 | 63951 | 0.891 | 0.336 |
| ADGRL4 | 64123 | 0.631 | 0.336 |
| CARD9 | 64170 | -0.710 | 0.336 |
| TSPAN14 | 81619 | 0.742 | 0.336 |
| PUS3 | 83480 | -0.771 | 0.336 |
| INSM2 | 84684 | 0.587 | 0.336 |
| PAPLN | 89932 | -0.473 | 0.336 |
| TGIF2LY | 90655 | 0.642 | 0.336 |
| TTC30A | 92104 | -0.682 | 0.336 |
| BPIFB1 | 92747 | 0.863 | 0.336 |
| EGLN2 | 112398 | 0.637 | 0.336 |
| SPATA45 | 149643 | -0.773 | 0.336 |
| ASPRV1 | 151516 | 0.718 | 0.336 |
| RIBC1 | 158787 | 0.696 | 0.336 |
| MPZL3 | 196264 | 0.938 | 0.336 |
| SUN3 | 256979 | 0.804 | 0.336 |
| HIST2H2AB | 317772 | 0.570 | 0.336 |
| AGRN | 375790 | -0.441 | 0.336 |
| C6orf201 | 404220 | -0.909 | 0.336 |
| LGALS7B | 653499 | -0.978 | 0.336 |
| RGPD2 | 729857 | 0.505 | 0.336 |
| ELOA3D | 100506888 | 0.853 | 0.336 |
| LOC102723996 | 102723996 | -0.204 | 0.336 |
| LOC102725035 | 102725035 | 0.464 | 0.336 |
| LOC105371566 | 105371566 | -0.838 | 0.336 |
| ELOA3C | 107983955 | 0.853 | 0.336 |
| LOC107987425 | 107987425 | 0.457 | 0.336 |
| HSP90AB1 | 3326 | 0.570 | 0.336 |
| TACC1 | 6867 | 0.512 | 0.336 |
| PARP2 | 10038 | -0.439 | 0.336 |
| QPCT | 25797 | 0.595 | 0.336 |
| CILP2 | 148113 | 0.490 | 0.336 |
| RGPD1 | 400966 | 0.509 | 0.336 |
| SARDH | 1757 | -0.549 | 0.336 |
| SIRPB1 | 10326 | 0.491 | 0.336 |
| MAST2 | 23139 | -0.615 | 0.336 |
| SVEP1 | 79987 | -0.390 | 0.336 |
| MAATS1 | 89876 | -0.587 | 0.336 |
| ICOSLG | 23308 | -0.204 | 0.336 |
| PHF21B | 112885 | -0.202 | 0.336 |
| LAMC1 | 3915 | -0.071 | 0.336 |
| SLC2A3 | 6515 | -0.071 | 0.336 |
| TEX15 | 56154 | -0.142 | 0.336 |
| TREML1 | 340205 | -0.062 | 0.336 |
| SLC2A14 | 144195 | -0.071 | 0.336 |
| CTSG | 1511 | 0.018 | 0.336 |
| SMC4 | 10051 | -0.019 | 0.336 |
| NCCRP1 | 342897 | 0.002 | 0.336 |
| DSC3 | 1825 | -0.006 | 0.336 |
| BLVRB | 645 | -2.581 | 0.335 |
| LSAMP | 4045 | 1.780 | 0.334 |
| C6 | 729 | 0.077 | 0.334 |
| CADPS2 | 93664 | 0.813 | 0.333 |
| ZNF425 | 155054 | -1.410 | 0.333 |
| CA3 | 761 | -0.793 | 0.333 |
| DNAH11 | 8701 | 0.316 | 0.333 |
| HP | 3240 | -0.109 | 0.330 |
| NME9 | 347736 | 1.009 | 0.330 |
| TMCO5A | 145942 | -1.968 | 0.329 |
| PPIB | 5479 | 1.418 | 0.329 |
| DSC2 | 1824 | -1.173 | 0.329 |
| EPB41L2 | 2037 | 2.183 | 0.328 |
| ODF2 | 4957 | 2.198 | 0.328 |
| TRIM37 | 4591 | 1.754 | 0.327 |
| EFCAB6 | 64800 | 1.848 | 0.323 |
| UBC | 7316 | -0.615 | 0.322 |
| APOF | 319 | -0.266 | 0.322 |
| FDFT1 | 2222 | -1.893 | 0.321 |
| KRT14 | 3861 | -0.515 | 0.321 |
| PGK2 | 5232 | 3.383 | 0.319 |
| UBB | 7314 | -0.733 | 0.318 |
| KRT8 | 3856 | -2.485 | 0.318 |
| PKM | 5315 | 1.662 | 0.318 |
| FRA10AC1 | 118924 | 0.827 | 0.318 |
| UBA52 | 7311 | -0.804 | 0.318 |
| RPS27A | 6233 | -0.820 | 0.317 |
| DIS3 | 22894 | 1.349 | 0.316 |
| PLXDC2 | 84898 | -2.022 | 0.316 |
| DKK3 | 27122 | -0.753 | 0.315 |
| AK7 | 122481 | -2.752 | 0.312 |
| TUT4 | 23318 | -0.688 | 0.310 |
| CRP | 1401 | 0.321 | 0.307 |
| MYRF | 745 | 1.742 | 0.303 |
| F7 | 2155 | 1.544 | 0.303 |
| HPSE | 10855 | 0.655 | 0.302 |
| LZIC | 84328 | 0.944 | 0.301 |
| BLMH | 642 | 3.288 | 0.296 |
| FAM3C | 10447 | -0.759 | 0.292 |
| TIMP2 | 7077 | 2.105 | 0.292 |
| MYBL1 | 4603 | -0.910 | 0.292 |
| MCOLN3 | 55283 | 2.080 | 0.290 |
| PRKDC | 5591 | 1.088 | 0.290 |
| CEP83 | 51134 | 0.726 | 0.289 |
| LCP1 | 3936 | 0.164 | 0.286 |
| ABI3BP | 25890 | 0.774 | 0.286 |
| ANKRD28 | 23243 | -2.350 | 0.286 |
| OLFM1 | 10439 | -0.884 | 0.285 |
| PDIA3 | 2923 | 2.385 | 0.285 |
| PHACTR2 | 9749 | -1.881 | 0.284 |
| PAK1IP1 | 55003 | 1.957 | 0.282 |
| TKT | 7086 | 1.478 | 0.281 |
| ARHGAP42 | 143872 | 0.798 | 0.281 |
| MUC16 | 94025 | 1.452 | 0.280 |
| BPTF | 2186 | -1.640 | 0.279 |
| FLT4 | 2324 | -1.461 | 0.277 |
| CDH2 | 1000 | 2.402 | 0.277 |
| LEKR1 | 389170 | 2.260 | 0.276 |
| KRT77 | 374454 | 1.394 | 0.272 |
| SBSN | 374897 | 0.599 | 0.272 |
| DNAH9 | 1770 | 1.217 | 0.271 |
| COL18A1 | 80781 | 0.874 | 0.267 |
| FGD4 | 121512 | 0.940 | 0.267 |
| KIF19 | 124602 | 2.587 | 0.267 |
| SBNO2 | 22904 | -1.459 | 0.266 |
| MST1 | 4485 | -0.153 | 0.264 |
| ADAMTS13 | 11093 | -1.693 | 0.262 |
| SLC3A2 | 6520 | 1.167 | 0.259 |
| CHI3L1 | 1116 | 0.901 | 0.259 |
| PCDH15 | 65217 | 2.952 | 0.258 |
| ARHGAP22 | 58504 | -2.139 | 0.256 |
| F12 | 2161 | 1.213 | 0.254 |
| CHD5 | 26038 | 1.397 | 0.253 |
| TREH | 11181 | 0.717 | 0.252 |
| VWF | 7450 | 0.142 | 0.251 |
| JCHAIN | 3512 | 3.664 | 0.251 |
| APAF1 | 317 | 2.441 | 0.251 |
| PRDX2 | 7001 | -1.969 | 0.249 |
| APOM | 55937 | 0.112 | 0.245 |
| C1QA | 712 | 0.233 | 0.243 |
| HAUS1 | 115106 | 3.719 | 0.243 |
| ICAM1 | 3383 | 1.686 | 0.243 |
| IL32 | 9235 | -2.811 | 0.239 |
| IGFBP3 | 3486 | -0.187 | 0.238 |
| ILK | 3611 | 2.382 | 0.238 |
| SPARC | 6678 | 0.538 | 0.237 |
| GCN1 | 10985 | -0.980 | 0.236 |
| CARD8 | 22900 | 1.641 | 0.233 |
| SAA1 | 6288 | 1.383 | 0.228 |
| GP5 | 2814 | 0.714 | 0.228 |
| NUP214 | 8021 | -1.835 | 0.226 |
| VNN1 | 8876 | 1.452 | 0.226 |
| DSG1 | 1828 | 2.388 | 0.224 |
| NCF2 | 4688 | -0.908 | 0.223 |
| SERPING1 | 710 | 0.073 | 0.220 |
| COLEC11 | 78989 | 1.878 | 0.220 |
| FCGR3B | 2215 | 1.784 | 0.219 |
| SRPRA | 6734 | 2.476 | 0.219 |
| LUM | 4060 | 0.077 | 0.218 |
| CGAS | 115004 | -3.688 | 0.216 |
| MCAM | 4162 | 0.392 | 0.214 |
| DNTT | 1791 | -2.163 | 0.214 |
| CPN1 | 1369 | 0.072 | 0.209 |
| CLU | 1191 | 0.092 | 0.209 |
| EFEMP1 | 2202 | -0.433 | 0.205 |
| TLN1 | 7094 | 2.164 | 0.204 |
| ITIH1 | 3697 | 0.079 | 0.204 |
| PTPRJ | 5795 | 1.609 | 0.199 |
| LRIG1 | 26018 | -2.322 | 0.199 |
| MYH11 | 4629 | -1.146 | 0.198 |
| STBD1 | 8987 | 2.524 | 0.198 |
| TRAK2 | 66008 | 1.874 | 0.196 |
| MICA | 100507436 | -2.887 | 0.196 |
| PPARD | 5467 | -2.461 | 0.195 |
| PLCH1 | 23007 | 2.021 | 0.194 |
| TSR1 | 55720 | -1.659 | 0.194 |
| CUL1 | 8454 | -0.421 | 0.194 |
| PLTP | 5360 | 0.193 | 0.191 |
| CTSZ | 1522 | 3.057 | 0.191 |
| HPR | 3250 | -0.527 | 0.191 |
| PEX1 | 5189 | -1.547 | 0.190 |
| ITIH4 | 3700 | -0.069 | 0.189 |
| PLA2G7 | 7941 | 2.131 | 0.189 |
| STOM | 2040 | 1.309 | 0.187 |
| KRT32 | 3882 | -3.148 | 0.187 |
| C1QB | 713 | 0.116 | 0.187 |
| SERPINA1 | 5265 | -0.080 | 0.183 |
| EPHA4 | 2043 | -1.634 | 0.183 |
| LAMP1 | 3916 | 2.315 | 0.183 |
| ASAP2 | 8853 | 1.866 | 0.183 |
| PDZD2 | 23037 | 3.939 | 0.182 |
| NCAM2 | 4685 | 1.022 | 0.181 |
| HSPG2 | 3339 | -0.399 | 0.179 |
| ERVW-1 | 30816 | -2.642 | 0.177 |
| KIAA0100 | 9703 | 1.156 | 0.177 |
| NCAPH | 23397 | -1.685 | 0.177 |
| HLA-C | 3107 | 1.654 | 0.176 |
| FCN2 | 2220 | 0.169 | 0.175 |
| SYMPK | 8189 | 1.358 | 0.174 |
| SIRPA | 140885 | 1.165 | 0.174 |
| MMP9 | 4318 | 1.835 | 0.173 |
| FANCI | 55215 | 1.726 | 0.173 |
| ACTA1 | 58 | -2.263 | 0.173 |
| ACTC1 | 70 | -2.263 | 0.173 |
| CDK9 | 1025 | 1.839 | 0.171 |
| SEL1L3 | 23231 | -1.578 | 0.171 |
| HSPA2 | 3306 | -1.204 | 0.171 |
| ZNF479 | 90827 | 2.013 | 0.171 |
| C4B_2 | 100293534 | -3.341 | 0.171 |
| ZNF292 | 23036 | -1.617 | 0.170 |
| ZNF804B | 219578 | 1.663 | 0.170 |
| UGP2 | 7360 | -1.470 | 0.170 |
| NME1-NME2 | 654364 | -1.560 | 0.170 |
| NRCAM | 4897 | 1.480 | 0.169 |
| KRT17 | 3872 | 2.095 | 0.169 |
| NME1 | 4830 | -1.692 | 0.169 |
| NME2 | 4831 | -1.738 | 0.169 |
| RELN | 5649 | 0.753 | 0.168 |
| PRDX6 | 9588 | -1.720 | 0.168 |
| MYH2 | 4620 | 1.374 | 0.168 |
| TMEM200C | 645369 | 1.845 | 0.168 |
| COG4 | 25839 | -1.735 | 0.168 |
| STAU1 | 6780 | 1.704 | 0.168 |
| ART3 | 419 | -1.712 | 0.167 |
| EEF2K | 29904 | 1.628 | 0.167 |
| SLC44A5 | 204962 | 1.403 | 0.167 |
| CHGA | 1113 | 1.349 | 0.167 |
| ALAD | 210 | -1.599 | 0.167 |
| CLCN6 | 1185 | -1.841 | 0.167 |
| SERPINA2 | 390502 | 2.808 | 0.167 |
| MGAT1 | 4245 | -1.379 | 0.166 |
| ITGA2 | 3673 | 1.118 | 0.166 |
| YWHAH | 7533 | -1.592 | 0.166 |
| NCOA7 | 135112 | 1.449 | 0.166 |
| AK1 | 203 | -1.731 | 0.166 |
| KLHDC7A | 127707 | -2.364 | 0.166 |
| CNTN4 | 152330 | 1.166 | 0.166 |
| 12-Sep | 124404 | -1.750 | 0.166 |
| NENF | 29937 | 1.423 | 0.166 |
| TALDO1 | 6888 | 1.358 | 0.166 |
| HLA-G | 3135 | 1.868 | 0.166 |
| TRIM9 | 114088 | 1.269 | 0.166 |
| SERPINB1 | 1992 | -1.333 | 0.166 |
| ABHD11 | 83451 | 1.985 | 0.165 |
| C5 | 727 | 0.107 | 0.165 |
| PKP1 | 5317 | -1.182 | 0.165 |
| ERAP2 | 64167 | -1.243 | 0.165 |
| LRCH1 | 23143 | -1.595 | 0.165 |
| VIT | 5212 | -1.920 | 0.165 |
| PARVB | 29780 | 1.310 | 0.165 |
| RNF133 | 168433 | -1.861 | 0.165 |
| FASTK | 10922 | -1.612 | 0.165 |
| RTN4RL2 | 349667 | 1.330 | 0.165 |
| CDHR5 | 53841 | -1.342 | 0.165 |
| SOD2 | 6648 | 1.363 | 0.165 |
| RND3 | 390 | 1.908 | 0.165 |
| KIFC3 | 3801 | 1.869 | 0.165 |
| GOT1 | 2805 | 1.248 | 0.165 |
| CCL14 | 6358 | -1.987 | 0.165 |
| PARD3 | 56288 | -1.431 | 0.165 |
| ELOA3 | 162699 | 1.666 | 0.165 |
| ELOA3B | 728929 | 1.666 | 0.165 |
| CTSC | 1075 | -1.399 | 0.165 |
| PDZD3 | 79849 | 1.706 | 0.165 |
| NELFA | 7469 | 1.884 | 0.165 |
| GGCT | 79017 | 1.886 | 0.165 |
| VASP | 7408 | -1.289 | 0.165 |
| PIK3R5 | 23533 | 1.530 | 0.165 |
| AP4B1 | 10717 | 1.460 | 0.165 |
| TET3 | 200424 | 1.802 | 0.165 |
| RIMS2 | 9699 | -1.339 | 0.165 |
| FAM186B | 84070 | -1.697 | 0.165 |
| KRT7 | 3855 | 2.198 | 0.165 |
| P4HTM | 54681 | -1.342 | 0.165 |
| SLC5A9 | 200010 | -1.534 | 0.165 |
| PLEKHA6 | 22874 | 2.591 | 0.165 |
| HPD | 3242 | -1.445 | 0.165 |
| COPB1 | 1315 | -2.634 | 0.165 |
| HSP90AA1 | 3320 | 1.345 | 0.165 |
| KIAA1217 | 56243 | -2.213 | 0.164 |
| TAF7L | 54457 | 1.840 | 0.164 |
| SELENBP1 | 8991 | 1.845 | 0.163 |
| PDE7A | 5150 | -1.638 | 0.163 |
| HIST1H2BN | 8341 | 2.124 | 0.163 |
| MYO5B | 4645 | -1.271 | 0.161 |
| CKM | 1158 | 2.694 | 0.161 |
| TAF1 | 6872 | -1.337 | 0.159 |
| ITGB1 | 3688 | 1.770 | 0.159 |
| FTL | 2512 | -1.478 | 0.158 |
| ITGB3 | 3690 | 1.123 | 0.158 |
| SOD3 | 6649 | 1.902 | 0.158 |
| TCERG1 | 10915 | -2.974 | 0.157 |
| TSC1 | 7248 | -1.784 | 0.156 |
| DCDC2B | 149069 | 2.717 | 0.155 |
| SCLT1 | 132320 | 4.154 | 0.154 |
| HIST2H2BF | 440689 | 2.124 | 0.153 |
| BPGM | 669 | -2.453 | 0.152 |
| CPS1 | 1373 | 1.229 | 0.151 |
| IGFBP6 | 3489 | 0.244 | 0.151 |
| HIST1H2BH | 8345 | 2.160 | 0.150 |
| HIST1H2BO | 8348 | 2.160 | 0.150 |
| RASL11A | 387496 | -2.403 | 0.148 |
| MSN | 4478 | 2.183 | 0.148 |
| PLG | 5340 | 0.110 | 0.148 |
| UGT8 | 7368 | -1.813 | 0.146 |
| SMCHD1 | 23347 | 1.776 | 0.146 |
| ACIN1 | 22985 | -2.677 | 0.146 |
| LRFN2 | 57497 | -2.744 | 0.145 |
| S100A6 | 6277 | -3.565 | 0.144 |
| IFIT1 | 3434 | 2.444 | 0.144 |
| ALPI | 248 | -2.877 | 0.143 |
| IFIT5 | 24138 | 2.508 | 0.142 |
| MYO18B | 84700 | 2.791 | 0.142 |
| NARS | 4677 | 3.806 | 0.141 |
| HIST1H4I | 8294 | 1.998 | 0.141 |
| HIST1H4A | 8359 | 1.998 | 0.141 |
| HIST1H4D | 8360 | 1.998 | 0.141 |
| HIST1H4F | 8361 | 1.998 | 0.141 |
| HIST1H4K | 8362 | 1.998 | 0.141 |
| HIST1H4J | 8363 | 1.998 | 0.141 |
| HIST1H4C | 8364 | 1.998 | 0.141 |
| HIST1H4H | 8365 | 1.998 | 0.141 |
| HIST1H4B | 8366 | 1.998 | 0.141 |
| HIST1H4E | 8367 | 1.998 | 0.141 |
| HIST1H4L | 8368 | 1.998 | 0.141 |
| HIST2H4A | 8370 | 1.998 | 0.141 |
| HIST4H4 | 121504 | 1.998 | 0.141 |
| HIST2H4B | 554313 | 1.998 | 0.141 |
| APCS | 325 | 0.120 | 0.141 |
| REV1 | 51455 | -4.337 | 0.140 |
| SORCS2 | 57537 | 1.812 | 0.139 |
| ITGA2B | 3674 | 2.548 | 0.139 |
| IFIT2 | 3433 | 2.659 | 0.138 |
| IKBKB | 3551 | -3.601 | 0.137 |
| TPM4 | 7171 | -1.997 | 0.136 |
| LYVE1 | 10894 | 2.597 | 0.135 |
| PFN1 | 5216 | 2.379 | 0.133 |
| C2 | 717 | 0.079 | 0.132 |
| FBXO41 | 150726 | 3.345 | 0.132 |
| TGOLN2 | 10618 | -1.760 | 0.131 |
| LCAT | 3931 | 0.133 | 0.130 |
| SELP | 6403 | 2.377 | 0.129 |
| MAN1A1 | 4121 | 0.342 | 0.129 |
| HIST1H2BG | 8339 | 2.225 | 0.128 |
| HIST1H2BF | 8343 | 2.225 | 0.128 |
| HIST1H2BE | 8344 | 2.225 | 0.128 |
| HIST1H2BI | 8346 | 2.225 | 0.128 |
| HIST1H2BC | 8347 | 2.225 | 0.128 |
| HIST2H2BE | 8349 | 2.225 | 0.128 |
| COLEC10 | 10584 | 3.831 | 0.128 |
| C3 | 718 | 0.086 | 0.125 |
| AZGP1 | 563 | 0.097 | 0.125 |
| C4B | 721 | 0.189 | 0.125 |
| MMRN1 | 22915 | 0.359 | 0.124 |
| CD93 | 22918 | -2.746 | 0.124 |
| A2M | 2 | 0.109 | 0.123 |
| TXN | 7295 | 1.724 | 0.122 |
| AFDN | 4301 | 2.417 | 0.120 |
| SUMO4 | 387082 | 0.728 | 0.119 |
| CLSTN1 | 22883 | 2.317 | 0.118 |
| PCOLCE | 5118 | 2.774 | 0.117 |
| PPFIA2 | 8499 | -1.724 | 0.114 |
| DOCK11 | 139818 | 0.306 | 0.113 |
| GBGT1 | 26301 | -3.154 | 0.110 |
| SERPINA5 | 5104 | 0.147 | 0.108 |
| DSC1 | 1823 | 2.956 | 0.108 |
| THBS1 | 7057 | 0.697 | 0.108 |
| FAH | 2184 | 2.040 | 0.108 |
| TNXB | 7148 | 0.543 | 0.107 |
| DNAH8 | 1769 | 2.461 | 0.107 |
| ITIH2 | 3698 | 0.104 | 0.106 |
| CDH6 | 1004 | 3.353 | 0.105 |
| HIST1H2AE | 3012 | 2.532 | 0.104 |
| HIST1H2AD | 3013 | 2.532 | 0.104 |
| HIST1H2AI | 8329 | 2.532 | 0.104 |
| HIST1H2AK | 8330 | 2.532 | 0.104 |
| HIST1H2AJ | 8331 | 2.532 | 0.104 |
| HIST1H2AL | 8332 | 2.532 | 0.104 |
| HIST1H2AC | 8334 | 2.532 | 0.104 |
| HIST1H2AB | 8335 | 2.532 | 0.104 |
| HIST1H2AM | 8336 | 2.532 | 0.104 |
| HIST2H2AA3 | 8337 | 2.532 | 0.104 |
| HIST2H2AC | 8338 | 2.532 | 0.104 |
| HIST1H2AG | 8969 | 2.532 | 0.104 |
| H2AFJ | 55766 | 2.532 | 0.104 |
| HIST1H2AH | 85235 | 2.532 | 0.104 |
| HIST3H2A | 92815 | 2.532 | 0.104 |
| HIST2H2AA4 | 723790 | 2.532 | 0.104 |
| BARD1 | 580 | -4.042 | 0.102 |
| BLOC1S6 | 26258 | 2.269 | 0.099 |
| OAF | 220323 | 2.720 | 0.096 |
| STIP1 | 10963 | 2.210 | 0.096 |
| FGB | 2244 | -2.919 | 0.095 |
| MAP9 | 79884 | -2.660 | 0.095 |
| B3GAT3 | 26229 | -2.620 | 0.094 |
| RRP12 | 23223 | 1.981 | 0.093 |
| LRIG3 | 121227 | -2.120 | 0.093 |
| HSPA1L | 3305 | -2.046 | 0.091 |
| XIRP2 | 129446 | -3.947 | 0.091 |
| SERPINA11 | 256394 | -0.652 | 0.090 |
| PCF11 | 51585 | 2.155 | 0.090 |
| APOC1 | 341 | 0.227 | 0.089 |
| PF4V1 | 5197 | 0.457 | 0.088 |
| PF4 | 5196 | 0.457 | 0.088 |
| TMEM131L | 23240 | 1.684 | 0.088 |
| TJP2 | 9414 | -2.643 | 0.087 |
| SERPINA4 | 5267 | 0.153 | 0.087 |
| CYP27C1 | 339761 | 1.469 | 0.086 |
| NPAT | 4863 | 2.859 | 0.085 |
| NOP9 | 161424 | 2.612 | 0.085 |
| CALD1 | 800 | 2.023 | 0.085 |
| EYS | 346007 | -2.119 | 0.084 |
| C4A | 720 | -0.027 | 0.084 |
| RARRES2 | 5919 | -2.457 | 0.084 |
| BCORL1 | 63035 | 2.261 | 0.084 |
| SLC4A9 | 83697 | -2.242 | 0.083 |
| SIVA1 | 10572 | -3.109 | 0.083 |
| IGF2 | 3481 | -2.521 | 0.083 |
| SPATA17 | 128153 | 2.641 | 0.083 |
| SFTPA2 | 729238 | 1.985 | 0.083 |
| SFTPA1 | 653509 | 1.998 | 0.083 |
| QSOX1 | 5768 | 0.193 | 0.082 |
| PNRC1 | 10957 | 2.767 | 0.082 |
| CRY1 | 1407 | -3.311 | 0.082 |
| ALG13 | 79868 | 3.028 | 0.082 |
| CYP27B1 | 1594 | 2.506 | 0.081 |
| CDK5RAP2 | 55755 | 2.645 | 0.081 |
| HSPA6 | 3310 | -2.320 | 0.081 |
| RIMS4 | 140730 | 2.755 | 0.080 |
| ERBB2 | 2064 | -4.140 | 0.078 |
| CHL1 | 10752 | 0.285 | 0.077 |
| SAA2 | 6289 | 3.318 | 0.077 |
| MED14 | 9282 | 2.277 | 0.075 |
| PCYOX1 | 51449 | 0.266 | 0.075 |
| GSN | 2934 | 0.116 | 0.073 |
| ANKRD30B | 374860 | -2.640 | 0.072 |
| CDH1 | 999 | 3.146 | 0.071 |
| MMP2 | 4313 | 2.978 | 0.069 |
| SERPIND1 | 3053 | 0.109 | 0.069 |
| APOC2 | 344 | 0.361 | 0.068 |
| DAG1 | 1605 | 4.138 | 0.068 |
| CRTAC1 | 55118 | -1.182 | 0.066 |
| SPATS2L | 26010 | 3.085 | 0.065 |
| LTA4H | 4048 | 2.961 | 0.064 |
| VTN | 7448 | 0.116 | 0.064 |
| COL6A3 | 1293 | 2.669 | 0.061 |
| APOB | 338 | 0.147 | 0.060 |
| SELL | 6402 | 0.165 | 0.059 |
| NECTIN2 | 5819 | -4.572 | 0.059 |
| IGFALS | 3483 | 0.135 | 0.059 |
| SHBG | 6462 | -0.483 | 0.055 |
| CEP152 | 22995 | -5.239 | 0.054 |
| ANGPTL3 | 27329 | 3.894 | 0.053 |
| LPA | 4018 | 0.871 | 0.011 |
| APOC3 | 345 | 0.509 | 0.005 |
| PPBP | 5473 | 0.471 | 0.049 |
| SPP2 | 6694 | 0.470 | 0.042 |
| APOC4 | 346 | 0.405 | 0.019 |
| CNDP1 | 84735 | 0.330 | 0.003 |
| APOE | 348 | 0.278 | 0.032 |
| APOA4 | 337 | 0.273 | 0.022 |
| HGFAC | 3083 | 0.228 | 0.026 |
| APOL1 | 8542 | 0.215 | 0.027 |
| RBP4 | 5950 | 0.212 | 0.004 |
| HRG | 3273 | 0.182 | 0.014 |
| F10 | 2159 | 0.177 | 0.049 |
| SERPINF1 | 5176 | 0.176 | 0.040 |
| C8A | 731 | 0.173 | 0.036 |
| CFD | 1675 | 0.163 | 0.025 |
| TTR | 7276 | 0.156 | 0.007 |
| PON1 | 5444 | 0.156 | 0.025 |
| APOA1 | 335 | 0.150 | 0.042 |
| CLEC3B | 7123 | 0.112 | 0.019 |
