# Supplementary material for: Intermittent fasting from dawn to sunset for four consecutive weeks induces anticancer serum proteome response and improves metabolic syndrome
Source: Sci Rep. 2020 Oct 27;10:18341. doi: 10.1038/s41598-020-73767-w (PMC7592042; doi:10.1038/s41598-020-73767-w)
Supplement: Supplementary file 5 — Supplementary Table 4. [file 41598_2020_73767_MOESM5_ESM.docx]

**Intermittent Fasting from Dawn to Sunset for Four Consecutive Weeks Induces Anticancer Serum Proteome Response and Improves Metabolic Syndrome**

Ayse L. Mindikoglu, M.D., M.P.H.^1, 2^; Mustafa M. Abdulsada, M.B.Ch.B.^1^; Antrix Jain, M.S.^3^; Prasun K. Jalal, M.D.^1, 2^; Sridevi Devaraj, Ph.D.^4^; Zoe R. Wilhelm, B.S.^1^, Antone R. Opekun, M.S., P.A.-C ^1, 5^, Sung Yun Jung, Ph.D.^3, 6^

**Institutions:**

1. Margaret M. and Albert B. Alkek Department of Medicine, Section of Gastroenterology and Hepatology, Baylor College of Medicine, Houston, TX
2. Michael E. DeBakey Department of Surgery, Division of Abdominal Transplantation, Baylor College of Medicine, Houston, TX
3. Advanced Technology Core, Mass Spectrometry Proteomics Core, Baylor College of Medicine, Houston, TX
4. Clinical Chemistry and Point of Care Technology, Texas Children’s Hospital and Health Centers, Department of Pathology and Immunology, Baylor College of Medicine, Houston, TX
5. Department of Pediatrics, Section of Gastroenterology, Nutrition and Hepatology, Baylor College of Medicine, Houston, TX
6. Department of Molecular & Cellular Biology, Baylor College of Medicine, Houston, TX
